# Unpacking food environment policy landscapes for healthier diets in “emerging” countries: the case of Viet Nam

**DOI:** 10.3389/fpubh.2025.1548956

**Published:** 2025-04-22

**Authors:** Brice Even, Trang Thi Thu Truong, Hang Thi Minh Thai, Huong Thi Mai Pham, Duong Thu Nguyen, Anh Thi Viet Bui, Christophe Béné

**Affiliations:** ^1^Food Environment and Consumer Behaviour, International Center for Tropical Agriculture, Hanoi, Vietnam; ^2^Center for Agricultural Policy, Institute of Policy and Strategy for Agriculture and Rural Development, Hanoi, Vietnam; ^3^School of Environment, Faculty of Science, The University of Auckland, Auckland, New Zealand; ^4^Food Environment and Consumer Behaviour, International Center for Tropical Agriculture, Cali, Colombia; ^5^Wageningen Economic Group, Wageningen University, Wageningen, Netherlands

**Keywords:** food system, food environment, healthy diet, food policy, public health, LMIC

## Abstract

**Objective:**

Food systems and food environments are evolving rapidly in Viet Nam, concurrently with significant shifts in dietary patterns and health outcomes. This study aims to identify critical gaps in the national regulatory framework governing food environment in Viet Nam and to propose actionable recommendations to overcome these gaps.

**Results:**

Using the Food Environment Policy Index from the INFORMAS network, we mobilized a transdisciplinary panel of 18 experts to co-analyze and assess policy evidence, as well as co-develop policy recommendations. The assessment, encompassing 35 indicators across six food environment domains, revealed substantial gaps: 74% of indicators scored low or very low, while only 26% scored medium or high. Key gaps were identified in food composition standards, marketing, labeling, and financial incentives. Recommendations from the experts focused on strengthening food composition standards, enhancing consumer education, and fostering inter-sectoral policy integration.

**Implications:**

This study provides a comprehensive evaluation of Viet Nam’s food environment policies and offers actionable recommendations to foster food environments conducive of healthier diets. Drawing on Viet Nam as a case study representative of challenges in other low- and middle-income countries, our findings highlight the importance of strong political commitment to prioritize public health over industry interests in order to create healthier, more equitable food environments and food systems.

## 1 Introduction

Unhealthy diets are a major contributor to the global burden of non-communicable diseases (NCDs), with poor diets accounting for approximately 11 million deaths annually (1). Over recent decades, global dietary patterns have shifted toward increased consumption of ultra-processed foods (UPF) loaded with sugars, refined grains, fats, and salt. This shift has coincided with a worrying rise in diet-related chronic conditions, including overweight and obesity, which affected 2.5 billion and 890 million adults, respectively, in 2022 (2). Type 2 diabetes prevalence has also risen sharply, affecting 537 million people in 2021 (3). Notably, in the past 40 years, no country has reported a decline in obesity or diabetes prevalence (4).

Viet Nam has not been exempted from these trends. While undernutrition problems persist (including micronutrient deficiencies, stunting and wasting), overweight and obesity rates have surged, especially among children and adolescents, with prevalence rising from 8.5 and 2.5% in 2010 to 19 and 8.1% in 2020, respectively; equivalent to a stunning annual growth rate of 8.4% (5). This triple burden of malnutrition highlights the urgent need to better understand and address the evolving food environments –the interface where consumers interact with the food system and make dietary choices (6, 7). Unhealthy diets have been linked to suboptimal food environments, especially those where unhealthy foods and beverages are largely available, accessible, and affordable (8–10).

Over the past few decades, Viet Nam’s food environment has changed rapidly, with an increased availability of oils, fats, sugar, and beverages (11). Concurrently, nutrient-rich foods such as lean meats, fish, vegetables, and fruits have become disproportionately expensive compared to energy-dense foods (12), creating affordability barriers for healthier food choices, particularly for low-income households (11). While traditional wet markets remain key sources of fresh, nutritious foods in Viet Nam (13, 14), the expansion of formal modern retail has accelerated, with convenience stores and supermarkets expanding significantly (15, 16), from 897 outlets in 2012 to 3,272 by 2017 (17). These shifts reflect broader global trends where rapid changes in food environments have led to increased availability, accessibility, affordability, and consumption of UPF (18–21).

A growing number of governments started recognizing the urgency to address rising obesity and NCDs, subsequently adopting policy measures to respond to and reorient the evolving food environments. For example, Mexico’s adopted a sugar-sweetened beverages (SSBs) tax that led to reduced consumption of sugary drinks (22), while Chile restricted food marketing to children, alongside front-of-package warning labels, as part of a comprehensive strategy to curb the consumption of unhealthy foods (23). In Asia, Thailand’s excise tax on SSBs has also influenced consumption trends (24, 25)

In Viet Nam, multi-sectoral food system policies are emerging, such as Decision 300/QD-TTg approving the *National Action Plan on Food System Transformation* (26), but the food environment concept remains largely absent from policy discourse. In addition, while previous research examined important related issues such as nutrition policies, the impacts of trade agreements on nutrition and health, or food safety regulations (27–30), the full range of policies influencing food environments has not been comprehensively assessed. This study aims to fill that gap.

For this purpose, we adapted and utilized the Food Environment Policy Index from the INFORMAS network; a tool designed to examine and identify policy strengths and gaps in national regulatory frameworks (31). Through the engagement of an interdisciplinary panel of experts into a participatory process, we aimed to answer the following research questions:

- To what extent have different food environment domains been addressed in public policies, strategies, and orientations at the national level in Viet Nam?
- What actionable policy recommendations can be developed to address the identified policy gaps and enhance Viet Nam’s food environment policy framework?

By addressing these questions, our study aims to provide a comprehensive analysis of the policy landscape influencing food environments in Viet Nam and to identify a series of actionable policy propositions. With these, we seek to contribute to the emerging dialogue on food environment policies, offering evidence-based guidance for policymakers as they work toward healthier and more sustainable food systems. We also believe that this Viet Nam case study, being an example of an emerging economy where food environments and dietary patterns have been transforming rapidly over the last decades, offers valuable insights and potential lessons for other countries in similar stages of food system evolution.

## 2 Methods

### 2.1 Conceptual background: the food environment concept as a research framework

As defined above, food environments refer to the interface where food consumers engage with the food system. In this study, we define food environments as the conjunction of six domains: food products properties, food outlet properties, food marketing, food desirability, food prices and affordability, and food availability and accessibility. This definition aligns with existing definitions and frameworks (6, 10, 32), and is very close to the conceptualization proposed by Turner et al. (33).

**Figure 1:**
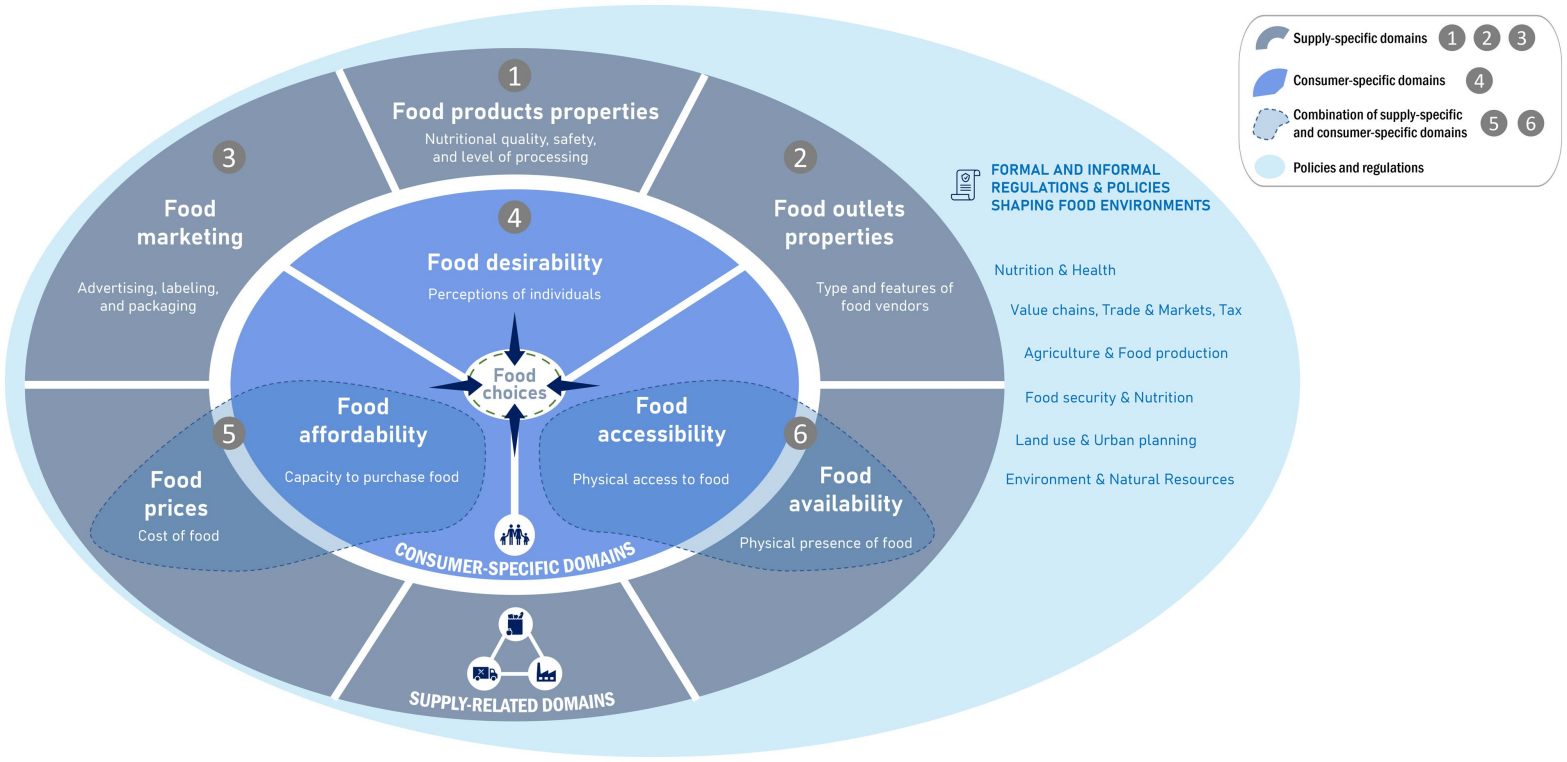
The food environment framework used to structure our analysis. Authors’ adaptation of Turner et al. (33).

Our adaptation (see Figure 1) refines Turner’s framework with the objective to enhance its applicability to policy analysis by making key structural modifications:

- Food product properties and food outlet properties have been disaggregated to distinguish between policies targeting food composition (e.g., nutrient profiles, processing levels) and those regulating retail structures (e.g., store types, locations, and operating models).
- Food marketing has been separated from regulation to reflect their distinct roles. While Turner’s framework links marketing with regulations, we treat marketing as a standalone domain of the food environment, recognizing its direct influence on consumer behavior through advertising, branding, labeling, and promotions. Regulations, in contrast, shape but do not constitute the food environment itself; they extend beyond marketing to other aspects of the food environment. This distinction allows for a clearer analysis of how marketing strategies shape food choices independently of regulatory interventions.
- Food availability and accessibility have been combined into a single domain, recognizing that physical presence (availability) and ease of acquisition (accessibility) are inherently interconnected in shaping food environments.
- Food prices and affordability have been combined into a single domain, given their close relationship in determining economic access to food. Prices reflect supply-side dynamics, while affordability relates to consumers’ purchasing power.

### 2.2 A staged process

**Figure 2:**
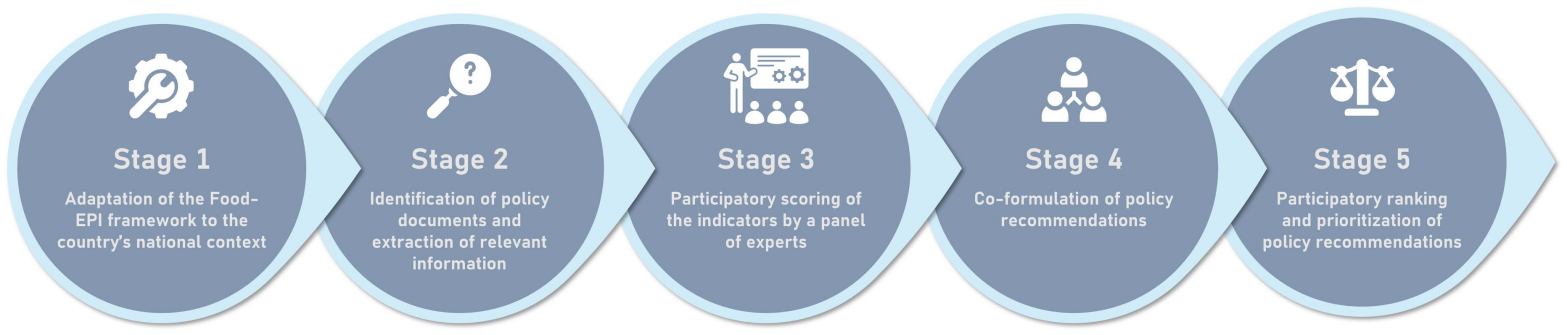
Overview of the methodological stages adopted for this study. Source: Authors’ own elaboration.

To apply the Food Environment Policy Index (Food-EPI) tool, developed by the International Network for Food and Obesity/Non-communicable Diseases Research, Monitoring and Action Support (INFORMAS), we designed a five-stage process tailored to our study. This process is summarized in Figure 2 and detailed in the following sub-sections.

#### 2.2.1 Stage 1: adaptation of the food-EPI framework to the national context

**Figure 3:**
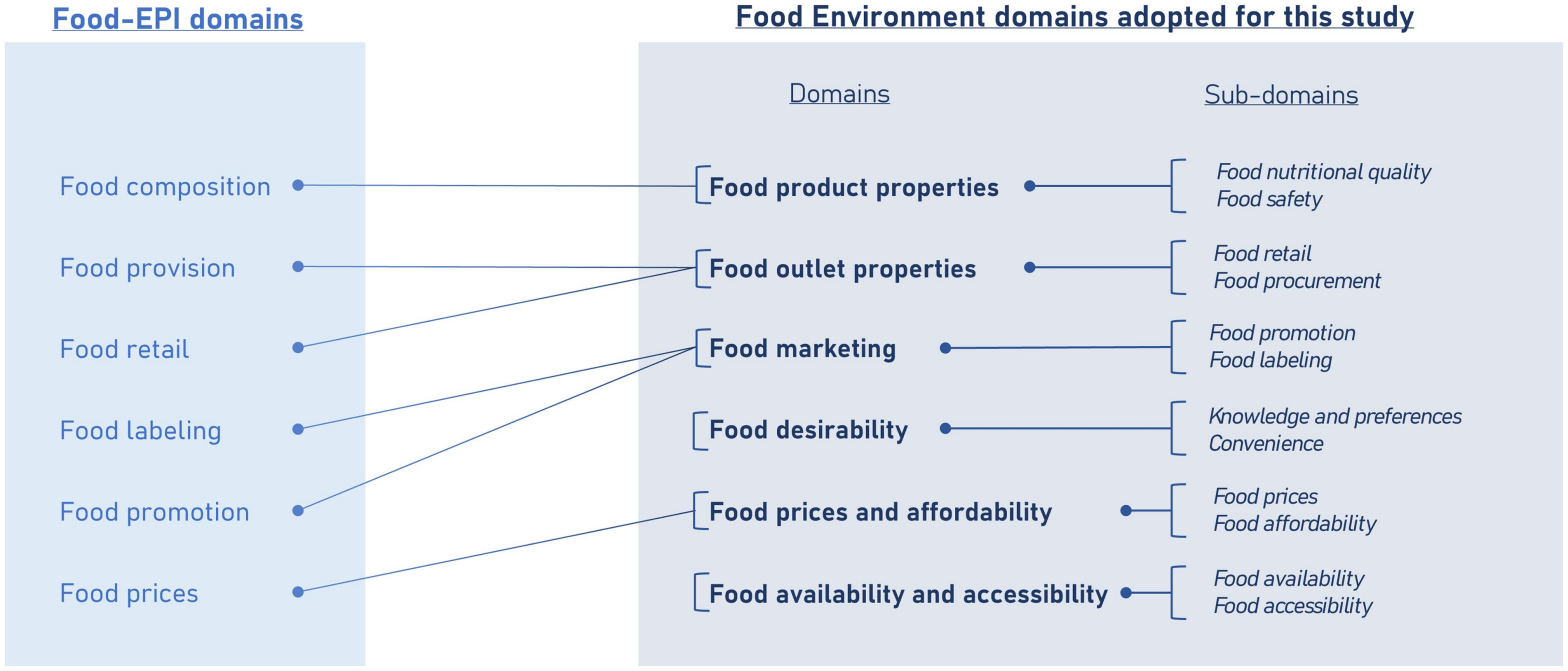
Adaptations of the food-EPI domains to the study scope. Source: Authors’ own elaboration.

We adapted the policy domains of the Food-EPI tool to better fit with our food environment theoretical framework (see Figure 3). This included the addition of two domains, namely food desirability and food availability and accessibility. Food desirability captures factors like nutritional knowledge and food preferences (10, 33) while food availability and accessibility encompass the factors that determine the extent to which individuals have access to nutritious food (33, 34).

Within each food environment domain, a set of indicators was developed, primarily drawn from the Food-EPI tool. These indicators were then reviewed and reorganized to align with our food environment domains. Additional indicators were added to capture Vietnam-specific aspects, including food safety–identified as an important component, especially given its influence on consumers’ food choice in Viet Nam (35, 36). Ultimately, our final tool consisted of 35 indicators (see Supplementary material 1).

#### 2.2.2 Stage 2: identification of policy documents and extraction of relevant information

Because we could not search for policy documents labeled as ‘food environments’ policies (as the concept is not integrated in current Vietnamese policies), we adopted an exploratory approach to identify relevant policy documents. This included a search of policies across six key sectors: (i) nutrition and health; (ii) agriculture and food production; (iii) value chains, trade and markets, and tax; (iv) food security and nutrition; (v) land use and urban planning; and (vi) environment and natural resources.

**Figure 4:**
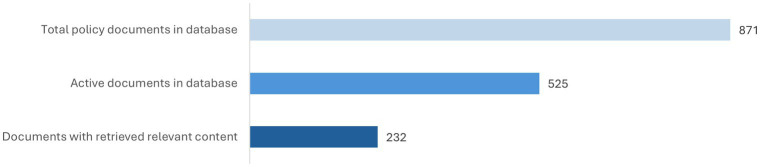
Total, active, and relevant policy documents in the database.

The search, which focused on official policy documents such as laws, decisions, decrees, directives, and circulars (see Supplementary material 2), was conducted between December 2022 and May 2023 and resulted in a total of 871 documents (see Figure 4). To ensure the relevance of the dataset, we filtered out expired or superseded policies, retaining 525 active documents (i.e., policies currently in force).

A keyword-based screening process was then applied to the documents. For each food environments domain, a structured list of keywords was developed (see Supplementary material 3), initially in English and subsequently translated into Vietnamese.

The screening was performed, using *WordStat9* and *QDAMiner* software, part of the *Provalis Suite^®^* (37), using keywords to automatically retrieve relevant text segments from the policy documents. This automated process scanned all policy documents for keyword matches. A total of 232 documents revealed containing relevant content (see Figure 4). Relevant text segments were extracted and compiled into a structured spreadsheet, allowing the categorization of the Vietnamese government’s policy measures in relation to each indicators (see Supplementary material 4).

Two independent analysts systematically reviewed all extracted text segments to assess their pertinence. Segments that did not explicitly address food environment-related aspects or lacked meaningful policy implications were excluded. After data cleaning, relevant segments were consolidated into an evidence report (see Supplementary material 5) presenting a coherent summary of the government’s measures for each indicator. To ensure the accuracy of the information, the draft evidence report was circulated among experts from the National Institute of Nutrition (NIN) and the Vietnamese Academy of Agricultural Sciences (VAAS) for review.

#### 2.2.3 Stage 3: participatory scoring of the indicators by a panel of experts

In a subsequent phase, we mobilized a panel of 18 experts (comprising academics, government officials, and representatives of NGOs) to score the indicators based on the evidence report as well as their own expertise and knowledge. The purpose of this scoring exercise was to assess the extent to which the current food environment-related policies address each indicator in a systematic way, identifying strengths, gaps, and areas for improvement in relation to international best practices. To ensure the integrity of the process and mitigate potential conflicts of interest, no representatives from the food industry were included. Experts were selected through purposive sampling, utilizing the authors’ professional networks to identify individuals with specialized knowledge in specific fields directly relevant to food environment dimensions, such as food production, processing, trade, safety, nutrition, and health (see Supplementary material 11). Both researchers and practitioners actively engaged in policy development and practice were considered. Participants were invited to join the panel via phone or email.

Prior to the engagement with the experts, a 4-level Likert scale-based scoring system was used by the research team to assign provisional scores to each indicator (see Supplementary material 6). These scores reflected the extent to which current Vietnamese policies collectively address and fulfil the requirements of each indicator. The scoring criteria considered policy recognition of the issue, specificity of measures, and presence of enforcement mechanisms; ranging from “*no acknowledgment*” of the issue (score D) to “*a comprehensive legal framework with specific measures and clear enforcement processes*” (score A). These provisional scores were then incorporated into the evidence report, along with examples of international best practice sourced from a collection produced by the INFORMAS network.

This compiled material was then distributed to all experts via email for review before the series of final scoring workshops (using the aforementioned 4-level Likert scale). The primary objective of these workshops (which took place in September 2023) was to reach consensus on the scoring of each of the 35 indicators. Through this consensus-building process, the panel aimed to align perspectives on priority actions and prepare the ground for the formulation of a cohesive set of policy recommendations.

#### 2.2.4 Stage 4: co-formulation of policy recommendations

A search of existing listings of policy recommendations related to food environment enhancement was completed, using previous publications related to the implementation of the Food-EPI index (38–41), and broader literature on food environment policy interventions (42–45). A preliminary list of 40+ policy recommendations pertinent to the Vietnamese context was then prepared by the research team. This draft list was then shared with the members of the expert panel, who were invited to provide written feedback and suggest any additional policy recommendations they deemed relevant. A workshop was held in May 2024 to further refine and revise the draft list of recommendations. Resulting from this process, a final list of 38 policy recommendations was proposed (see Supplementary material 7).

#### 2.2.5 Stage 5: participatory ranking and prioritization of policy recommendations

The final step involved the ranking and prioritization of the policy recommendations, utilizing a structured approach based on six specific criteria, covering four sets of criteria (see Box 1; Supplementary material 8).

Box 1 **The four sets of criteria used to score the policy recommendations**

**- “Relevance to the Vietnamese context”** criterion was used to evaluate the alignment of each policy recommendation with Vietnam’s specific institutional, socio-cultural, and economic landscape, ensuring that the proposed policies are appropriate and acceptable within the local context.

**- “Feasibility of implementation”** criterion was used to assess the practicality of enacting and executing the recommended policies across Vietnam. It included considerations such as the compatibility of the policies with existing legal frameworks and the resources required for successful implementation.

**- “Potential for achieving impact”** criterion was used to evaluate the expected effectiveness of the policies in improving nutritional and health outcomes at the population level, highlighting the impact each policy could have on public health.

**- “Urgency for action”** criterion was used to evaluate policies based on the immediacy of the issues they address, emphasizing the need for timely intervention in response to pressing health challenges in Vietnam.

The final list of 38 proposed policy recommendations, along with the ranking instruments, were distributed to the experts before the ranking workshop. During the workshop, each criterion was evaluated using a Likert scale, with 0 indicating the lowest score and 10 the highest. For each policy recommendation, a composite score index was then computed by aggregating the scores assigned by each expert across all criteria into a single metric.

## 3 Results

### 3.1 Policy gaps

**Figure 5:**
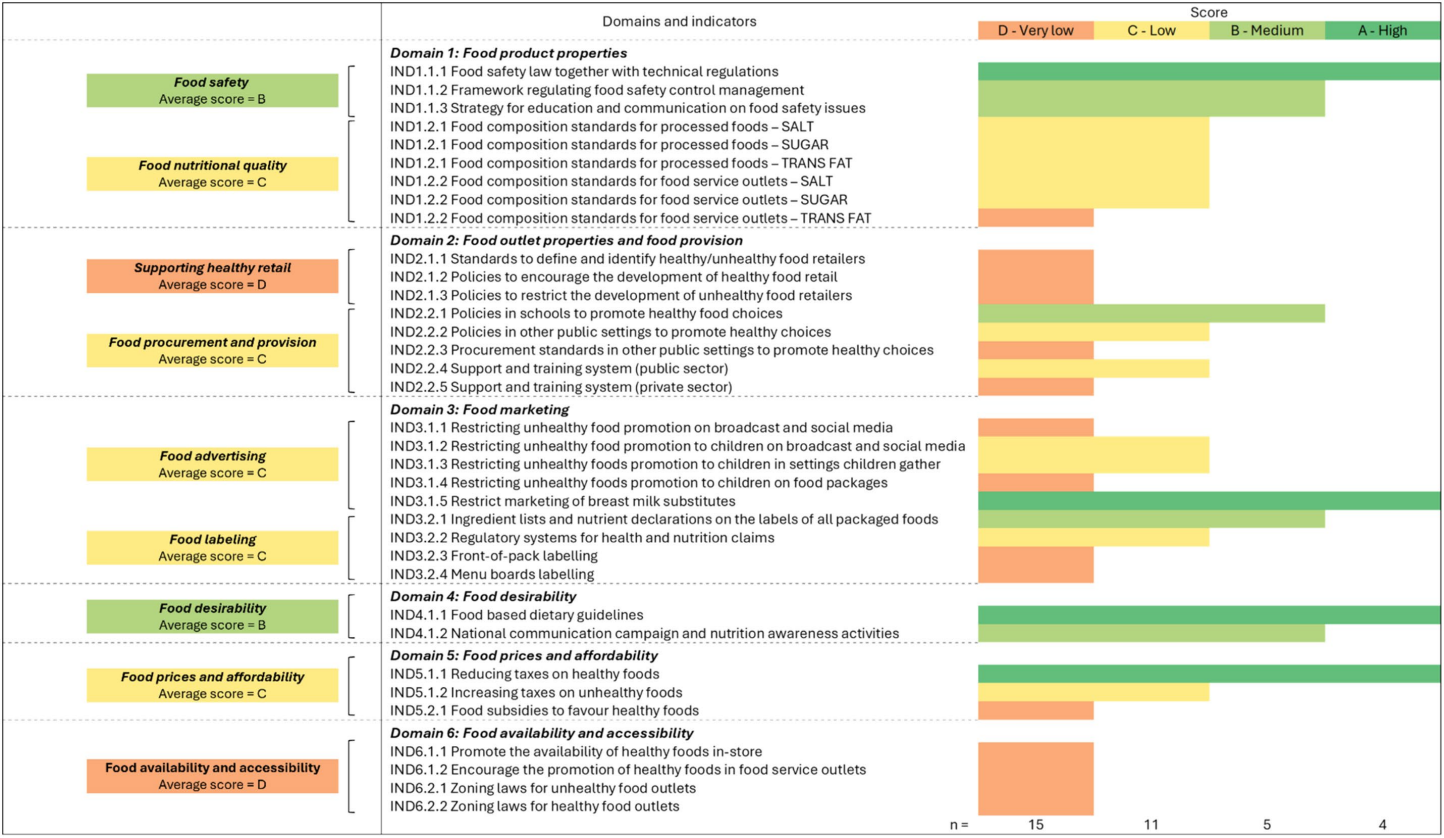
Overview of the scores for the 35 indicators. (1) Color code: *High* (A—green): the indicator/issue is comprehensively addressed in current Vietnamese policies, with clear implementation and enforcement mechanisms; *Medium* (light green—B): the indicator is partially addressed, with limited guidance and enforcement mechanisms; *Low* (yellow—C): the indicator is acknowledged without being addressed through policies; *Very low* (orange—D): the indicator is not acknowledged in existing policies. (2) The aggregated results displayed in the figure reflect the consensus reached by experts during the co-analysis of the evidence report.

Figure 5 presents the results of the participatory scoring exercise for each of the six food environment domains used in this study (as per the set of four criteria discussed in section 2.2.3 and listed in Supplementary material 6). The analysis reveals that Viet Nam falls significantly short of international best practices in several critical policy areas. Among the 35 indicators assessed, 43% (*n* = 15) received very low scores, 31% (*n* = 11) received low scores, 14% (*n* = 5) were rated as intermediate, and only 12% (*n* = 4) achieved high scores. Specifically, indicators related to food composition standards, food outlet properties, food marketing, food labeling, financial incentives, and accessibility to healthy food received very low to low scores, indicating substantial gaps compared to international benchmarks.

The subsequent subsections present syntheses of the co-analysis conducted as part of the rest of this analysis. Each subsection corresponds to one of the six food environment domains defined in our analytical framework (cf. figure 3). These syntheses are concise, with more comprehensive analyses provided in Supplementary material 5.

#### 3.1.1 Food products properties

Food nutritional quality indicators received an average score of C (low), indicating limited policy action in this area. Viet Nam has been implementing a limited number of policies addressing the nutritional quality of processed foods, with a primary focus on salt reduction (e.g., *Decision 376/QD-TTg* and *Decision 2033/QD-BYT*). However, these policies lack precise targets, enforcement mechanisms, and clarity on oversight responsibilities. Regulations on sugar and trans-fat content remain even more underdeveloped, with no clear standards or formal restrictions in place.

Food safety indicators received an average score of B (medium), reflecting notable policy advancements. In response to persistent food safety challenges (46), the Vietnamese government has implemented structural reforms, including the enactment of the *Food Safety Law 55/2010/QH12*, which established clearer regulatory responsibilities along with standards and technical regulations. Despite these advancements, significant enforcement challenges persist, particularly due to limited inspection capacity at lower administrative levels and inconsistent implementation of food safety communication and training.

#### 3.1.2 Food outlet properties

Indicators on regulating healthy and unhealthy food retail outlets received an average score of D (very low). Current policies do not differentiate between retailers offering healthy versus unhealthy food options. National policies generally promote modern retail expansion (e.g., *Decision 5078/QD-BCT*, *Decision 1,163/QD-TTg*), without specific provisions to regulate unhealthy food availability in convenience stores, or vending machines. On the other hand, informal traditional retail channels, including street vendors near schools, face increasing restrictions (e.g., *Joint Circular 08/2008/TTLT-BYT-BGDĐT, Plan 266/KH-UBND*), undermining their potential role in providing affordable, fresh food.

Food procurement and food provision indicators received an average score of C (low) reflecting the absence of robust policies ensuring food nutritional quality in both public and private settings. While the Food Safety Law provides general regulations to ensure food safety in food procurement and provision, specific measures addressing nutritional quality remain limited. School food policies exist (e.g., *Decision 712/QD-TTg*, *Decision 2195/QD-BGDDT*), but most measures remain non-mandatory, leading to inconsistent implementation across educational institutions. Beyond school settings, there are significant gaps in regulating food service activities in other public and private institutions. Workplace and hospital meal guidelines (e.g., *Decision 2879/QD-BYT*, *Resolution 07c/NQ-BCH*) exist but are not legally binding.

#### 3.1.3 Food marketing

Food advertising indicators received an average score of C (low), reflecting limited regulatory measures to restrict the promotion of unhealthy foods in all settings. Vietnamese policies acknowledge the need to regulate unhealthy foods advertising, particularly around schools (e.g., *Decision 1,294/QD-BYT*). However, existing regulations (e.g., *Advertising Law 16/2012/QH13, Decree 38/2021/NĐ-CP*) do not explicitly restrict the promotion of unhealthy foods and beverages. Current advertising restrictions primarily target breast milk substitutes (e.g., *Decree 181/2013/ND-CP*), leaving other unhealthy products largely unregulated.

Food labeling indicators received an average score of C (low). Existing food labeling regulations require packaged food products to include ingredient lists and nutritional information (e.g., *Decree 43/2017/ND-CP*) but enforcement remains weak due to the lack of clear mechanisms for review and approval of food labels.^1^ Health claims on processed food are also poorly regulated, with current provisions only applying to functional foods (e.g., *Circular 43/2014/TT-BYT*). Front-of-pack labeling remains voluntary, with no clear implementation guidelines (e.g., *Decision 5,924/QD-BYT*) limiting consumers access to clear, at-a-glance nutrition information.

#### 3.1.4 Food desirability

Indicators related to food desirability received an average score of B (medium), reflecting the government’s strong emphasis on consumers’ education. Viet Nam’s nutritional policies emphasize public awareness campaigns to improve dietary choices (e.g., *Decision 1,294/QD-BYT*, *Decision 02/QD-TTg*, *Decision 5,924/QD-BYT*). These efforts promote dietary guidelines through mass media, social networks, and targeted education programs. They also highlight the importance of nutrition education within public health initiatives and emphasize training for health communication staff at both central and local levels. However, these policies remain largely non-mandatory, leading to inconsistencies and limited implementation.

#### 3.1.5 Food prices and affordability

Indicators related to food prices and affordability received an average score of C (low), reflecting limited policy efforts to make healthier foods more affordable and the absence of fiscal measures to discourage unhealthy food consumption. The government has implemented several tax exemptions for minimally processed food (e.g., *Law 13/2008/QH12*, *Decree 129/2022/ND-CP*), that might have an effect on their price. However, there are no direct financial incentives to promote consumers’ healthier dietary choices. Although several policy documents (e.g., *Decision 376/QD-TTg, Decision 1,092/QD-TTg*) underscore the importance of designing and implementing suitable tax rates to limit the consumption of sugary drinks and processed foods, no such excise tax have been implemented to date. Additionally, Viet Nam’s subsidy policies prioritize price stabilization for staple foods like rice, salt, and sugar, (e.g., *Law 11/2012/QH13, Resolution 34/NQ-CP*), but there are no targeted subsidies to improve the affordability of healthier food choices.

#### 3.1.6 Food availability and accessibility

Indicators related to food availability and accessibility received an average score of D (very low), highlighting the absence of policies that incentivize retailers to promote healthy food options or regulate the proliferation of unhealthy food outlets. We found no evidence of specific policies aiming at encouraging food retailers to actively promote the availability of healthy foods or limit unhealthy options. Similarly, we found no evidence of zoning laws and policies aimed at regulating the density of unhealthy food outlets or encouraging the development of outlets providing healthier options.

### 3.2 Policy recommendations

A total of 38 policy recommendations were co-developed (see Supplementary material 7) aimed at addressing previously identified policy gaps and improving Viet Nam’s food environment. While we acknowledge that some recommendations may have effects across multiple food environment domains, each recommendation has been mapped to a single food environment domain for clarity and analytical purposes. The recommendations were designed to be adaptable rather than prescriptive, aiming at providing guidance for national-level policy discussions. While some recommendations focus on specific settings (e.g., schools), this reflects their recognized importance as policy entry points for shaping dietary habits early in life rather than an intentional emphasis on any particular sector.

Table 1 presents the aggregated scores for each recommendation. Detailed scores for each criterion are available in Supplementary material 9.

**Table 1:** Average aggregated scores for 38 policy recommendations.

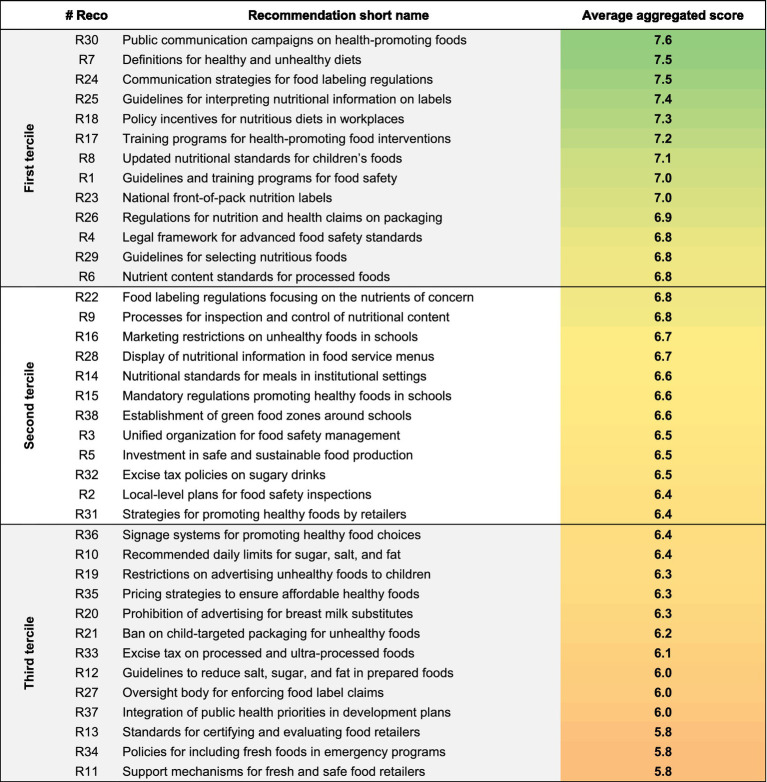

Scores range from 0 to 10, with 0 representing the minimum score and 10 representing the maximum score. The color scale progresses from orange (lower scores) to green (higher scores), visually indicating the relative strength of each score, with shades in between reflecting intermediate values.

All recommendations are characterized by an aggregated average score above 5.8 on the predefined 0–10 Likert scale, indicating that each recommendation was positively evaluated by the experts (i.e., scoring above the average threshold of 5). For analytical purposes, the recommendations were grouped into three terciles (cf. first column of Table 1) based on their scores. We used a fixed-threshold approach to classify policy recommendations into terciles. First Tercile includes the highest-scoring recommendations, representing those with the strongest alignment with the evaluative criteria, while the third Tercile contains the lowest-scoring recommendations, pointing to areas potentially considered less crucial by the panel of experts. This tercile-based analysis helps reveal potential patterns and supports the prioritization of policy recommendations.

The ranking indicates that recommendations achieving the highest overall scores predominantly focus on communication and awareness initiatives (e.g., R30, R24), training and guidelines (e.g., R25, R17, R29), and the establishment or update of standards and definitions (e.g., R7, R8, R6). In parallel, recommendations that involve greater control measures (e.g., R9, R2), regulatory frameworks (e.g., R22, R15), and restrictions (e.g., R16, R38, R32) tend to be situated in the second tercile. Finally, recommendations that entail even stricter restrictions, particularly those directed at the food industry (e.g., R36, R19, R35, R21, R33), as well as those involving complex policy processes (e.g., R27, R37), are predominantly located in the third tercile.

The data reveals experts’ apparent preferences for policies centered on public engagement and education, as opposed to those imposing regulatory constraints on food processors and distributors. Stricter regulatory approaches, particularly those involving advertising restrictions, pricing strategies, or taxation, received lower scores, reflecting concerns regarding their feasibility or their alignment with the Vietnamese context.

Notably, recommendations pertaining to the food desirability and food product properties domains received the highest average scores, indicating that experts viewed these domains as particularly critical and instrumental for achieving healthier food environments. The particularly high scores of the recommendations related to improving nutritional information and knowledge reflect the experts’ perceived importance of intervening on the demand side (i.e., shaping consumer preferences and behaviors toward healthier dietary choices), especially through educational and communication initiatives.

## 4 Discussion

This study employed a combination of evidence-based and participatory approaches to address two research questions: (1) To what extent have different food environment domains been addressed in Vietnamese policies, strategies, and orientations at the national level? And (2) What actionable policy recommendations can be developed to address the identified policy gaps and enhance Viet Nam’s food environment policy framework?

By examining policy gaps, this study highlights the limitations in Viet Nam’s regulatory framework for food environments, many of which reflect challenges commonly observed in emerging economies undergoing rapid food system transformations. In the subsequent sections, we first contextualize the main policy gaps identified in Viet Nam’s policy landscape and put them in perspective within the wider literature, in particular related to other emerging countries. We also discuss the actionable policy recommendations prioritized by the interdisciplinary panel of experts (See Boxes 2–5). The discussion concludes with reflections on the overarching need to prioritize public health interests over industry and economic profits, a challenge not unique to Viet Nam and relevant to many countries navigating similar stages of food environments and food system transformation.

### 4.1 Food product properties: expanding beyond food safety regulations to address broader nutritional risks posed by ultra-processed foods

In the past two decades regulations governing food production, processing, and trade have been largely focused on ensuring food safety, driven by significant public concern, extensive media coverage, and the stringent requirements of export markets (30, 46, 47). While food safety remains a crucial public health priority, the predominance of food safety-related policies has contributed to the lack of regulatory measures addressing other pressing public health issues, such as the rising prevalence of overweight and obesity (27), a shortcoming reflected in the low scores assigned to this domain. This pattern is not unique to Viet Nam. Similar trends have been observed in other countries, where food safety concerns often take precedence over dietary health, providing disincentives for producing high-quality and nutritious food, potentially limiting comprehensive responses to other dietary and health issues such as the growing obesity epidemic (48, 49).

In Vietnam, despite growing awareness of the health risks associated with excessive consumption of nutrients of concern (11), the regulatory framework remains underdeveloped. While the country has set general salt reduction objectives for the food industry, the absence of mandatory targets and clear enforcement mechanisms weakens the potential impact of these policies. Similar shortcomings have been observed in other LMICs, where voluntary guidelines, without mandatory regulations or precise targets, are considered less effective (50–54).

The regulatory framework surrounding sugar and trans-fat content is even less developed in Viet Nam. While sugar has been widely recognized as a major contributor to diet-related NCDs (55, 56), the adoption of formal regulations for specific sugar reduction targets in Vietnam remains slow and fails to adequately address the urgency of the issue. In contrast, other more advanced countries, like Mexico, have implemented stringent sugar reduction policies that include mandatory targets and taxation on SSBs, leading to a decrease of SSBs consumption, thus demonstrating the potential effectiveness of stronger regulatory approaches (22, 57, 58).

The lack of regulation surrounding trans-fat is also concerning given the well-documented health risks associated with trans-fat consumption, including increased risk of cardiovascular diseases (59, 60), and the evidence about the effectiveness of trans-fat bans on public health (61). In Viet Nam, the timid recognition of trans-fat as a public health issue and the lack of corresponding regulations, place the country at risk of falling behind international efforts to eliminate trans-fat from the food supply.

While addressing nutrient content is crucial, focusing solely on specific nutrients may overlook a broader public health issue: the degree of food processing (62, 63) (See Box 2). Evidence indicates that the health risks associated with UPF go beyond high levels of salt, sugar, and fat; Pagliai et al. (64) highlighted several studies suggesting that the adverse effects of UPFs may be attributed to other mechanisms, such as the presence of harmful compounds (e.g., acrylamide, acrolein, bisphenol A) that are generated during food processing and are therefore more prevalent in UPF (65, 66). Additionally, the organoleptic properties of UPFs, which promote rapid eating rates and delay satiety signaling, can lead to increased overall food intake (67). Despite reformulation efforts aimed at improving nutrient profiles, ultra-processed foods may continue to pose health risks. Thus, policy efforts should not only address specific nutrient content but also address broader issues related to UPF, including options that allow reduced availability and consumption of UPF.

Box 2 **Policy recommendations addressing food nutritional quality**

The high score assigned to the recommendation focused on **defining healthy and unhealthy diets (R7)** suggest a consensus among experts that standardized (but country-specific) definitions are essential to guide both regulatory actions and consumer choices. In a food environment increasingly penetrated by unhealthy foods, such clarity is fundamental. Similarly, recommendations calling for **nutritional standards for children’s foods (R8)** and **nutrient content standards for processed foods (R6)** also scored highly. By prioritizing these standards, experts appear to be advocating for stronger regulatory measures to limit the potential nutritional risks posed by processed foods. Establishing such standards could mark a shift in Vietnam’s food policies from a traditional focus on food safety toward a more comprehensive approach that addresses the nutritional quality of food products. The high score of the recommendation related to **processes for inspections and enforcement of nutritional content (R9)** also illustrates the experts’ view that setting standards alone is insufficient; effective enforcement mechanisms are crucial for ensuring compliance.

Interestingly, recommendations aimed at **reducing nutrient of concern -salt, trans-fat and sugar- in prepared food (R12)** received comparatively lower scores. This reflect the challenges Vietnam may face in implementing stringent ingredient-focused regulations, likely due to existing limitations in regulatory capacity and enforcement. Experts may view a phased or targeted approach as more feasible, focusing initially on broader nutritional standards before addressing specific nutrients/ingredients. Such a staged approach could allow Vietnam to build the necessary basis, setting the groundwork for more specific and ambitious regulations over time

### 4.2 Balancing the food retail transition with policy measures for more equitable access to fresh and healthy foods

While Viet Nam’s policies support investment in both developing modern food channels and upgrading traditional retail food infrastructure (e.g., Decree No. 02/2003/ND-CP, now replaced by Decree No. 60/2024/ND-CP), the upgrade of traditional markets has faced significant challenges in attracting investment. As a result, the expansion of modern food channels (e.g., convenience stores) has increasingly taken over the role of traditional food distribution channels (e.g., wet markets). While this may contribute to enhanced food safety, it potentially reduces access to fresh, healthy food options provided by wet markets, as well as informal and mobile vendors, such as those selling fruits (36, 155). It illustrates a broader trend within Vietnamese policy, where the promotion of modern retail often eclipses the need to preserve and enhance traditional food distribution channels, potentially limiting access to healthier food options (16). This imbalance is reflected in the low scores assigned to this domain, as policies have not sufficiently accounted for the need to ensure equitable food access across different retail formats.

This issue is not unique to Viet Nam. Research in other LMICs shows that unregulated modern retail expansion often leads to an increased access to ultra-processed and unhealthy foods, contributing to rising obesity rates and diet-related NCDs (20, 68, 69). Simultaneously, the relative neglect of traditional markets, which historically have been sources of fresh and less processed foods, exacerbates disparities in food affordability and accessibility, disproportionately affecting low-income populations (69–71). Policies aimed at preserving and strengthening traditional food markets (such as vendor support programs and infrastructure investments) are increasingly recognized as critical strategies for ensuring equitable food environments (16, 72).

Moreover, the absence of policies promoting the availability of healthy foods in retail and food service outlets raises concerns that the food environment may be dominated by less healthy options. Global evidence suggests that regulatory interventions increasing the availability and in-store promotion of healthy food options, such as fruits and vegetables, can drive positive dietary behaviors (73–76). However, Viet Nam currently lacks mechanisms to encourage or mandate such practices, reflecting a broader challenge observed in many LMICs where food environments remain largely shaped by market forces rather than public health objectives.

The absence of zoning laws or density regulations for unhealthy food outlets represents a missed opportunity. Evidence from other countries suggests that zoning laws limiting the concentration of fast-food outlets or incentivizing the establishment of stores selling fresh food products have proven effective in creating healthier food environments (73, 77). In Viet Nam, the unrestricted expansion of convenience stores and fast-food outlets, without counterbalancing policies to promote fresh food access (16), risks further entrenching obesogenic food environments (See Box 3).

Finally, Vietnamese policy documents related to food procurement and provision are scarce, except to some extent for school settings. However, the non-mandatory nature of these school nutrition guidelines raises concerns about consistency in their implementation. Evidence from other settings suggests that voluntary guidelines are less effective in shaping food environments compared to legally binding regulations (52). Additionally, the absence of comprehensive policies regulating food service activities in other public and private sectors underscores a critical gap, reflecting a broader global challenge in establishing robust food procurement regulations (78).

Box 3 **Policy recommendations targeting food outlets**

The expert panel prioritized **policy incentives for nutritious diets in workplaces (R18)** and **training programs for health-promoting food interventions (R17)**, reflecting a strategy focused on encouraging healthier dietary habits through capacity-building and incentive-driven initiatives. Recommendations in the middle tercile, such as **mandatory regulations promoting healthy foods in schools (R15)** and the **establishment of green food zones around schools (R38)**, suggest a willingness to create protective environments for children. The high scores on impact and urgency criteria for these recommendations indicate that experts recognize schools as critical settings where exposure to unhealthy food options needs to be reduced.

Conversely, recommendations that address changes within the food retail sector, such as **guidelines to reduce salt, sugar, and fat in prepared foods (R12), standards for certifying and evaluating food retailers (R13), support mechanisms for small-scale food retailers (R11)**, and **pricing strategies to ensure affordable healthy foods (R35)**, received lower prioritization. This may reflect the panel’s concern over the challenges (i.e., concerns over regulatory capacity, potential resistance from private stakeholders, complexity of enforcing these policies) associated with implementing such measures across the food retail sector. This hypothesis remains, however, difficult to confirm.

### 4.3 Addressing gaps in food messaging: marketing and labeling regulations should complement dietary guidelines and public awareness campaigns

The current regulatory framework in Viet Nam relies heavily on dietary guidelines and public awareness campaigns to promote healthier diets. This approach aligns with international recommendations for integrating education and dietary guidelines into public health strategies (79–81). However, the non-mandatory nature of many of these policies raises concerns about their potential efficacy in achieving long-term public health goals.

Global evidence suggests that while voluntary guidelines and public awareness campaigns can help improve consumer knowledge, they are insufficient in the absence of binding regulations that directly shape food environments (82–84). Studies show that mandatory policies, such as those regulating the nutritional content of school meals or restricting the marketing of unhealthy foods, are more effective in achieving significant improvements in public health outcomes (61, 85–87).

Our findings highlight several noticeable gaps in Viet Nam’s food marketing and labeling policies that could undermine public health objectives. The most pressing issue is the absence of specific restrictions on the advertisement of unhealthy food and beverage products to children (See Box 4). Internationally, there is a widespread recognition of the harmful effects of unhealthy food marketing to young consumers, especially given their greater susceptibility to advertising and limited ability to critically assess marketing messages (88–91). Viet Nam’s current regulatory framework, as in many LMICs, falls short of implementing effective controls. This gap is particularly concerning given the global evidence linking marketing of energy-dense, nutrient-poor foods to the rising prevalence of diet-related NCDs (86, 92, 93).

In terms of food labeling, Viet Nam has made substantial progress by requiring ingredient lists and nutritional information on packaged foods, which aligns with international standards (43, 94). However, the lack of mechanisms for reviewing and approving labels remains a significant weakness. Similarly, there are no mechanisms to review and approve health claims on food packages. This regulatory gap could lead to the proliferation of misleading nutrition and health claims. Given the potential influence of such claims on consumer behavior (95–97), particularly those that exaggerate the health benefits of certain products, it could create consumer confusion and encourage the purchase and consumption of products that are not genuinely health-promoting (98, 99). In the longer-term, it could even undermine the legitimacy and effectiveness of healthy food promotion.

Box 4 **Policy recommendations on food messaging and marketing**

The expert panel prioritized enhancing public nutrition awareness through non-restrictive, educational approaches. High-scoring recommendations, such as **public communication campaigns on health-promoting foods (R30)** and **guidelines for interpreting nutritional information on labels (R25)**, align with Vietnam’s existing focus on dietary guidelines and public awareness. These interventions were viewed as of primary importance for empowering consumers to make informed food choices.

Recommendations to **establish a national front-of-pack nutrition labeling system (R23)** and **regulate nutrition and health claims on packaging (R26)** also received high prioritization, reflecting the perceived need for stronger regulatory frameworks to improve labeling transparency and counteract misleading claims. Second tercile recommendations, including **food labeling regulations on nutrients of concern (R22)** and the **display of nutritional information in food service menus (R28)**, underline the perceived importance of extending nutritional information and labeling to food service establishments.

In contrast, more restrictive measures, such as **restrictions on advertising unhealthy foods to children (R19)** and a **ban on child-targeted packaging for unhealthy foods (R21)** received relatively lower scores. This lower prioritization may reflect the anticipated resistance from food industry stakeholders and perceived challenges in enforcing such policies, making these measures less feasible compared to communication- and education-based approaches. Overall, the prioritization reflects a strategic focus on empowering consumers and promoting voluntary dietary shifts (i.e., food demand side), while hesitations remain toward adopting stricter regulatory measures that directly confront food industry practices (i.e., food supply side).

### 4.4 Leveraging fiscal policies to enhance affordability of healthier food options

The absence of a targeted excise tax on sugary drinks and ultra-processed foods in Viet Nam is concerning, given WHO (100) recommendations and extensive evidence demonstrating the effectiveness of such taxes in reducing the purchase and consumption of unhealthy products [e.g., (58, 101, 102)]. Redondo et al. (58) found that taxes on SSBs have a significant impact on sales, reducing the purchase of such products. Niebylski et al. (102) review highlighted that taxation of unhealthy foods can increase the consumption of healthier alternatives by making nutrient-poor foods relatively less affordable. However, while the impact on purchasing and dietary behaviors is evident, the long-term effects of these taxes on public health outcomes, such as reductions in obesity and other diet-related non-communicable diseases (NCDs), remain less certain as these studies often do not track long-term health indicators. This underscores the need for continued research to determine the sustained impact of fiscal policies on long-term health outcomes (101, 103).

While the Vietnamese government’s recent consideration of introducing a special consumption tax on sugary beverages is a positive development, excluding UPF from taxation may undermine the overall effectiveness of the tax. Research suggests that a comprehensive approach, targeting both sugary drinks and other ultra-processed foods, would be more effective in improving public health (102). Several LMICs, including Mexico, Thailand, and South Africa, have implemented taxes on sugary beverages, with evidence showing reductions in purchases and increased consumer awareness of health risks (24, 104, 105). However, few LMICs have expanded such taxes to UPF, reflecting the challenge of overcoming industry resistance and ensuring political feasibility (106, 107).

In Viet Nam, tax exemption for non-and minimally processed foods represents a positive step toward promoting healthier diets. However, while these exemptions may help make fresh, unprocessed foods more affordable, they were not explicitly designed as a public health intervention to incentivize healthy dietary choices. Moreover, the existing set of subsidy policies which primarily support staple foods does not extend to healthier food options that are vital for preventing NCDs. The current focus on maintaining stable prices for essential staples like rice, salt, and sugar is crucial for food security, but without additional policies to make healthier foods more affordable, there is a risk of reinforcing unhealthy dietary patterns (See Box 5). It could disproportionately affect low-income households, which are already at higher risk of poor dietary choices due to financial constraints. This issue is not unique to Viet Nam. Many LMICs maintain subsidy programs aimed at food security rather than dietary quality, often focusing on staple crops while neglecting fruits, vegetables, and other nutrient-rich foods (108–110). Research also shows that without equitable economic access to healthy foods, socio-economic disparities in diet-related health outcomes can widen (18). Also, promoting foods like rice, or sugar, through subsidies might discourage the production of a more diverse range of crops, including fruits and vegetables, which are essential for a balanced diet (111). This could have long-term implications for the sustainability of local food systems and dietary diversity (112).

Box 5 **Policy recommendations on fiscal policies**

None of the recommendations related to food prices and affordability were ranked in the first tercile, reflecting precautions of the panel to support fiscal policies aimed at reshaping food consumption patterns. The **excise tax policy on sugary drinks (R32)** was ranked in the middle tercile, scoring relatively high on impact and urgency but lower on feasibility and relevance; indicating that experts view it as a moderately viable measure to improve food environments.

Broader fiscal measures, such as the **excise tax on processed and ultra-processed foods (R33)**, were ranked in the lower tercile. While scoring relatively well on impact, it received a low feasibility score, likely reflecting concerns over enforcement challenges and resistance from food industry stakeholders. Similarly, the recommendation to **include fresh foods in emergency programs (R34)** scored consistently low across all criteria, indicating limited perceived relevance, feasibility, and impact.

The higher prioritization of excise taxes on sugary drinks compared to broader taxes on ultra-processed foods might reflect a preference for targeted, incremental measures that are more feasible to implement. The perceived complexity of defining and enforcing broader taxes on ultra-processed foods underscores the potential need for a more phased approach. Future policy efforts could focus on piloting such measures to evaluate their feasibility and impact, providing a stronger evidence base for scaling up fiscal interventions in Vietnam.

### 4.5 Prioritizing public health over industry and economic interests

The current policy framework in Viet Nam, which, as our evidence shows, largely depends on non-mandatory measures, faces significant challenges in addressing the rising burden of diet-related NCDs. The gaps identified in the current policy framework suggest that there is significant room for improvement in attempts to create a healthier food environment in Viet Nam. While some progress has been made in promoting the consumption of healthier foods, the existing policies remain insufficient in restricting the availability, accessibility, affordability, and appeal of unhealthy food options. Moreover, practical and institutional barriers (including weak intersectoral coordination, limited regulatory oversight, and insufficient accountability mechanisms) hinder policy effectiveness. Without stronger, mandatory and enforceable regulations, it is unlikely that the country will effectively counter the rise in NCDs driven by unhealthy diets.

To advance public health outcomes, it will be important for the Vietnamese government to consider adopting comprehensive policies that not only promote the consumption of healthy foods, but also restrict unhealthy food options. This includes introducing stringent measures to limit the widespread distribution and appeal of ultra-processed and nutrient-poor foods. Regulatory action is necessary, as the food industry’s economic interests and political influence often result in resistance to improvements that prioritize public health (113–117). It is now evident that reliance on market mechanisms, along with deregulation and minimal government interventions, has contributed to the corporate concentration that dominate the food industry today (106, 118–120). Policy feasibility is thus frequently constrained by corporate lobbying and economic dependencies (121–123).

While globalization and liberalization have contributed to lower food prices, they have also posed serious challenges to public health, equity, and environmental sustainability (106, 124–126). The political economy of food governance presents significant barriers to effective regulatory enforcement. Big Food industries consistently resist constraining regulations through various tactics aimed at safeguarding their interests (127–130). These include notably the use of intensive lobbying strategies, public-private partnerships that blur regulatory boundaries, and corporate-led initiatives that frame voluntary self-regulation as a substitute for formal government action (21, 127, 131–133). These industry-led efforts have historically undermined the feasibility of public health regulations by delaying legislative processes, influencing decision-makers, and shaping public narratives around food policies (127, 134–137).

However, governments also bear responsibility for maintaining weak regulatory frameworks (138, 139). Policy inertia, policy ineffectiveness, conflicts of interest, and the prioritization of economic growth over public health frequently lead to inadequate actions against industry practices that undermine healthy diets (107, 134, 135, 140). In many cases, governments have actively facilitated the growth and market dominance of food corporations through policies that favor deregulation, trade liberalization, and corporate expansion, often at the expense of social justice and public health objectives (141–144). This is further evident in instances where governments align with industry interests to avoid the political costs of implementing stricter regulations, or due to the significant contributions these industries make to national economies through tax revenues, exports, or employment (136, 138, 145, 146). In Viet Nam, these dynamics are further reinforced by the significant economic contributions of the food and beverage sector, including employment generation, tax revenues, and export earnings (147); factors that may make policy-makers reluctant to introduce restrictive measures that might be perceived as anti-business.

For regulatory frameworks to effectively protect consumers and promote healthier diets, governments must place public health above industry and economic interests. This requires overcoming both corporate resistance and governance inefficiencies through clearer intersectoral policy integration, independent regulatory oversight, and stronger accountability mechanisms. Governments must dismantle structural barriers to transparent, inclusive, and accountable governance by fostering participatory bottom-up approaches in decision-making and actively prioritizing evidence-based policy interventions that address both corporate practices and systemic inequalities in food environments (8, 134, 142, 145, 148–150). To enhance policy feasibility, Viet Nam could also benefit from phased implementation strategies and independent monitoring mechanisms to mitigate corporate interference and ensure alignment with international best practices.

### 4.6 Potential limitations

The scope of our analysis focused on government orientations, strategies, and commitments as outlined in policy documents. While this provides insights into the intended direction of public food environment policy, it does not permit an evaluation of the actual effectiveness of these policies or their on-the-ground real impact. The discrepancy between policy goals and real-world outcomes can be significant (151) and must be acknowledged. Future studies could address this limitation by evaluating the operationalization and implementation of these policies.

Our findings are grounded in the evaluation of our participating experts. Although the scoring exercises were based on evidence, it inherently involves a certain level of subjectivity (152, 153). Expert judgments, while valuable, are shaped by individual experiences, perspectives, and knowledge, potentially introducing biases (154).

The Food-EPI tool primarily focuses on food environment policies related to nutrition and human health (31). Although these areas are crucial, social and environmental considerations are also integral to comprehensive food environment policy (32). Addressing this limitation in future research may involve expanding the scope of the tool or incorporating complementary frameworks that capture the environmental and social dimensions of food environments, allowing for a more holistic evaluation of regulatory frameworks. In addition, while we employed a comprehensive keyword search strategy, and ensured broad coverage by including synonyms and variations of key terms, there remains a possibility that some relevant content or policy documents were not captured due to differences in phrasing.

## 5 Conclusion

This study provides a comprehensive assessment of the food environment policy landscape in Viet Nam, offering insights into the challenges faced by emerging countries amid rapidly transforming food systems. By identifying key policy gaps and proposing concrete, actionable recommendations for policy interventions, it serves as a valuable resource for policy makers, civil society and scientific organizations seeking to drive policy change.

Viet Nam’s nutrition and health policies have taken important steps in recognizing the increasing burden of diet-related NCDs, but there is a growing need for them to be more integrated across sectors beyond health. Acknowledging the importance of multilevel and multi-sectoral approaches rather than relying on single domain solutions is critical for implementing strategic, coordinated government action. This requires incorporating nutrition and health considerations into all areas of government, such as city planning, economic development, agricultural and trade policies, and conducting nutrition and health impact assessments across these domains.

Addressing the rising consumption of unhealthy foods requires more than voluntary measures. Stricter regulations are needed to restrict the availability, accessibility, affordability, and appeal of nutrient-poor, ultra-processed foods alongside adequate resources to ensure compliance. Overcoming resistance from industry actors with vested economic interests will be critical to disrupt the status quo. Such a dual approach, promoting nutrient-dense foods while placing tighter restrictions on harmful ultra-processed options, is essential for fostering a healthier food environment and achieving meaningful public health outcomes.

Viet Nam’s experience highlights the challenges and opportunities faced by many LMICs undergoing rapid food system transformations. The policy gaps identified in this study—particularly the need for stronger regulatory measures, improved intersectoral coordination, and enhanced enforcement mechanisms—are common across emerging economies. Balancing food safety priorities with broader nutrition objectives, leveraging fiscal policies to promote healthier food choices, and addressing industry influence in policymaking are key takeaways that can inform policy reforms elsewhere. Ultimately, policy strategies must be both evidence-based and adaptable to the specific governance and economic contexts of each country to ensure meaningful and lasting improvements in food environments.

While this study provides actionable policy recommendations, further research is needed to evaluate policy implementation and long-term impact. Future studies could focus on assessing policy effectiveness through longitudinal analyses, identifying challenges in policy enforcement across administrative levels, and exploring strategies to enhance multi-sectoral collaboration.

## Data Availability

The data analyzed in this study is subject to the following licenses/restrictions: The data supporting the conclusions of this article will be made available by the authors upon request, provided that at least one author of this article is involved in any subsequent publication that utilizes these data. Requests to access these datasets should be directed to b.even@cgiar.org.

## Footnotes

^1^ Subsequent to data collection and analysis (which included updates up to May 2023), Viet Nam’s Ministry of Health issued Circular 29/2023/TT-BYT. This Circular provides guidelines for labeling nutritional components and values on prepackaged foods, stipulating that by December 31, 2025, producers, traders, and importers must include nutritional composition on food labels. This represents a significant step forward in Vietnam’s food labeling regulations.
