# Supplementary material for: Unpacking food environment policy landscapes for healthier diets in “emerging” countries: the case of Viet Nam
Source: Front Public Health. 2025 Apr 22;13:1548956. doi: 10.3389/fpubh.2025.1548956 (PMC12063741; doi:10.3389/fpubh.2025.1548956)
Supplement: Supplementary file 1 [file Data_Sheet_1.pdf]

Supplementary Material

1. **Supp. Mat. #1: List of indicators 2**
2. **Supp. Mat. #2: Definitions and scope of policy documents in Vietnam....................4**
3. **Supp. Mat. #3: List of keywords for each domain 6**
4. **Supp. Mat. #4: List of policy documents from which evidence was retrieved 8**
5. **Supp. Mat. #5: Evidence report 23**
6. **Supp. Mat. #6: Scoring system for indicators....................65**
7. **Supp. Mat. #7: List of policy recommendations....................67**
8. **Supp. Mat. #8: Ranking system for policy recommendations 69**
9. **Supp. Mat. #9: Detailed scores for all criteria across all recommendations 73**
10. **Supp. Mat. #10: Definitions of the food environments domains 75**
11. **Supp. Mat. #11: Affiliations and expertise of panel members 77**
12. **Supp. Mat. #12: Detailed findings on policy strengths and weaknesses....................78**

1. Supp. Mat. #1: List of indicators

Domain	Sub-domain	Indicator	Initially included in food-EPI
D1 Food product's properties	SD1.1 Food safety	**IND1.1.1** Existence of a national modern food safety law that establishes the legal framework for ensuring food safety governance and regulatory oversight	**No**
D1 Food product's properties	SD1.1 Food safety	**IND1.1.2** Existence of an operational framework for food safety control, specifying mechanisms for enforcement, inspection services and product testing	**No**
D1 Food product's properties	SD1.1 Food safety	**IND1.1.3** A strategy and plan have been developed for information, education and communication on food safety issues	**No**
D1 Food product's properties	SD1.2 Food nutritional quality	**IND1.2.1** Food composition targets/standards/restrictions have been established for the content of the nutrients of concern (trans fats, added sugars, salt, saturated fat) in industrially processed foods	**Yes**
D1 Food product's properties	SD1.2 Food nutritional quality	**IND1.2.2** Food composition targets/standards/restrictions have been established for the content of the nutrients of concern (trans fats, added sugars, salt, saturated fat) in meals sold from food service outlets	**Yes**
D2 Food outlet properties	SD2.1 Food retail	**IND2.1.1** Standards have been established to define and identify healthy/unhealthy food retailers	**No**
D2 Food outlet properties	SD2.1 Food retail	**IND2.1.2** Policies to support the development and modernization of healthy food retailers (i.e. retail channels that provide affordable fresh food (e.g. traditional channels such as wet markets)).	**No**
D2 Food outlet properties	SD2.1 Food retail	**IND2.1.3** Policies to restrict the development of unhealthy food retailers (i.e. retail channels that provide food rich in nutrients of concern (e.g. fast-food chains)).	**No**
D2 Food outlet properties	SD2.2 Food procurement	**IND2.2.1** Clear, consistent policies (including nutrition standards) in schools and early childhood education services for food service activities (canteens, food at events, fundraising, promotions, vending machines etc.) to provide and promote healthy food choices.	**Yes**
D2 Food outlet properties	SD2.2 Food procurement	**IND2.2.2** Clear, consistent policies in other public sector settings for food service activities (canteens, food at events, fundraising, promotions, vending machines, etc.) to provide and promote healthy food choices.	**Yes**
D2 Food outlet properties	SD2.2 Food procurement	**IND2.2.3** Clear, consistent procurement standards to be implemented in public sector settings for food service activities to provide and promote healthy food choices.	**Yes**
D2 Food outlet properties	SD2.2 Food procurement	**IND2.2.4** Training system to help schools and other public sector organisations and their caterers meet the healthy food service policies and guidelines.	**Yes**
D2 Food outlet properties	SD2.2 Food procurement	**IND2.2.5** Policies that actively encourage and support private companies to provide and promote healthy foods and meals in their workplaces.	**Yes**
D3 Food marketing	SD3.1 Food promotion	**IND3.1.1** Policies to restrict exposure and power of promotion of unhealthy foods through broadcast media (TV, radio) and online/social media.	**Yes**
D3 Food marketing	SD3.1 Food promotion	**IND3.1.2** Policies to restrict exposure and power of promotion of unhealthy foods to children (including adolescents) through broadcast media (TV, radio) and online/social media.	**Yes**
D3 Food marketing	SD3.1 Food promotion	**IND3.1.3** Policies to ensure that unhealthy foods are not commercially promoted to children (including adolescents) in settings where children gather (e.g. preschools, schools, sport, and cultural events).	**Yes**
D3 Food marketing	SD3.1 Food promotion	**IND3.1.4** Policies to ensure that unhealthy foods are not commercially promoted to children (including adolescents) on food packages.	**Yes**
D3 Food marketing	SD3.1 Food promotion	**IND3.1.5** Restrict marketing of breast milk substitutes	**No**
D3 Food marketing	SD3.2 Food labelling	**IND3.2.1** Policies to ensure that ingredient lists and nutrient declarations in line with NIN/WHO recommendations are present on the labels of all packaged foods.	**Yes**
D3 Food marketing	SD3.2 Food labelling	**IND3.2.2** Evidence-based regulations for reviewing and approving claims on foods, so that consumers are protected against unsubstantiated and misleading nutrition and health claims.	**Yes**
D3 Food marketing	SD3.2 Food labelling	**IND3.2.3** Policies to ensure that evidence-informed front-of-pack supplementary nutrition information system, which allow consumers to assess a product's healthiness, is applied to all packaged foods (e.g. Nutri-Score, Traffic light label).	**Yes**

D3 Food marketing	SD3.2 Food labelling	**IND3.2.4** Policies to set a simple and clearly visible system of labelling the menu boards of all quick service restaurants (i.e. fast-food chains), which allows consumers to interpret the nutrient quality and energy content of foods and meals on sale.	**Yes**
D4 Food desirability	SD4.1 Nutrition knowledge	**IND4.1.1** Existence of clear and evidenced-informed food based dietary guidelines supporting nutrition information and communication.	**Yes**
D4 Food desirability	SD4.1 Nutrition knowledge	**IND4.1.2** Policies supporting the establishment of national communication campaign and nutrition awareness activities	**Yes**
D5 Food prices and affordability	D5.1 Food prices	**IND5.1.1** Taxes on healthy foods are minimized to encourage healthy food choices (e.g. low or no sales tax, value-added or import duties on fruit and vegetables).	**Yes**
D5 Food prices and affordability	D5.1 Food prices	**IND5.1.2** Excise taxes on unhealthy foods (e.g. sugar-sweetened beverages, foods high in nutrients of concern) are in place and increase the retail prices of these foods to discourage unhealthy food choices.	**Yes**
D5 Food prices and affordability	D5.2 Food affordability	**IND5.2.1** The intent of existing subsidies on foods is to favour healthy rather than unhealthy foods	**Yes**
D6 Food availability and accessibility	SD6.1 Food availability	**IND6.1.1** Existence of policies to encourage food stores to promote the in-store availability of healthy foods and to limit the in-store availability of unhealthy foods.	**Yes**
D6 Food availability and accessibility	SD6.1 Food availability	**IND6.1.2** Existence of policies to encourage the promotion and availability of healthy foods in food service outlets and to discourage the promotion and availability of unhealthy foods in food service outlets.	**Yes**
D6 Food availability and accessibility	SD6.2 Food accessibility	**IND6.2.1** Zoning laws and policies to place limits on the density or placement of quick serve restaurants or other outlets selling mainly unhealthy foods in communities and/or access to these outlets (e.g. opening hours).	**Yes**
D6 Food availability and accessibility	SD6.2 Food accessibility	**IND6.2.2** Zoning laws and policies to encourage the development of outlets selling fresh fruit and vegetables and/or to increase access to these outlets (e.g. opening hours, frequency i.e. for markets).	**Yes**

2. Supp. Mat. #2: Definitions and scope of policy documents in Vietnam

Law: is a type of mandatory legal document with the highest legal value (after the Constitution), promulgated by the National Assembly to regulate basic issues. Law documents help specify the provisions of the Constitution for each specialized industry and field.

Resolution: a type of legal document, often used to decide on basic issues after being discussed and passed by a majority vote, expressing the opinion or intention of party agencies or organizations on a certain issue. Resolutions are usually in the form of documents of the National Assembly, the National Assembly Standing Committee, the Government, the Council of Judges of the Supreme People's Court, the People's Council, the People's Committees at all levels or of an agency, certain organization.

Decree: A type of legal document issued by the Government and used to guide laws or regulate arising matters that have not yet been regulated by any laws or ordinances. In addition, the Decree issued by the Government also stipulates the rights and obligations of the people according to the Constitution and Law.

Decision: A form of legal document, including legal normative documents and legal application documents issued by competent agencies and organizations. A Decision can be a legal document used to promulgate specific measures and regulations to implement the Party and State's guidelines and policies. At the same time, a Decision can also be a legal document management of a specific organization to promulgate specific content related to individuals and specific tasks of that organization.

Directive: An administrative document developed to express measures to direct, manage, and handle the affairs of organizations and agencies such as Ministries, Ministerial-level agencies, Government agencies, and the People's Committee people at all levels in the process of implementing state guidelines, policies, and laws.

Circular: A document that explains and guides the implementation of state documents issued and under the management of a specific sector.

Joint Circular: A document jointly issued by multiple relevant ministries, such as those pertaining to food safety management or food labeling.

Plan: A document issued by an agency, organization or individual have the authority to develop the content of work to be performed in a certain period of time to achieve a certain purpose.

Technical Regulations (QCVN): Regulations defining the limits of technical characteristics and management requirements that products, goods, services, processes, the environment, and other objects in socio-economic activities must adhere to. These regulations are established to ensure safety, hygiene, and human health; protect animals, plants, and the environment; and safeguard national interests, security, consumer interests, and other essential requirements. Technical regulations are formally promulgated in writing by a competent state agency and are mandatory for application.

Technical Standards (TCVN): Regulations outlining technical characteristics and management requirements used as a standard for classifying and appraising products, goods, services, processes, the environment, and other objects in socio-economic activities. The purpose of these standards is to enhance the quality and effectiveness of these objects. Technical standards are published in written form by an organization and are intended for voluntary application.

Note: Legal documents of a similar nature issued by different entities may exhibit variations in terms of scale and the extent of planning and implementation

The search was done on key online platforms, including the Government's Official Portal (https://chinhphu.vn) and websites of the Ministry of Agriculture and Rural Development (https://www.mard.gov.vn), the Ministry of Health (https://moh.gov.vn), and the Ministry of Industry and Trade (https://moit.gov.vn). Additionally, *Thư Viện Pháp Luật* (https://thuvienphapluat.vn), a comprehensive legal database, was utilized to identify supplementary documents and verify the validity of collected policies.

The search was complemented by leveraging policy databases from previous studies and projects, such as the SAFEGRO project database (focused on food safety regulations) and the IPSARD policy database (focused on agricultural production). This strategy ensured the comprehensiveness of data collection and enabled the inclusion of prior research insights.

3. Supp. Mat. #3: List of keywords for each domain

Domain	*Extensive list of keywords*	*Retained keywords for text retrieval (English)*	*Retained keywords for text retrieval (Vietnamese)*
Food products' properties	food ingredients; food substances; food composition; food quality; nutritious; nutritious food; food safety; organic; safe production; clean production; safe products; clean products; pesticides residues; veterinary drug residues; pathogens; heavy metals; shelf-life; nutrients; nutrient; micronutrients; nutrient-rich; nutritional quality; full of energy; energy dense; salt; sodium; fat; saturated fat; trans-fat; sugar; carbohydrate; ultra-processed food; processed food; food additives.	processed foods; pre-packaged foods; ultra-processed foods; salt; fats; sugar; trans fats; glucose; carbohydrates; food service outlets; canteen; restaurant; collective kitchen.	thực phẩm chế biến; thực phẩm bao gói sẵn; thực phẩm siêu chế biến; muối; chất béo; chất béo bão hòa; đường; đường glucose; chất bột đường; cửa hàng dịch vụ ăn uống; căngtin; nhà hàng; bếp ăn tập thể.
Food outlets' properties	food procurement; public procurement; canteen; food provision; food service policies; food at workplaces; school meal; shift meal; commercial kitchen; collective kitchen; food retail; food outlets; food markets; farmers' market; wet markets; toad markets; supermarkets; convenience stores; street vendors; mobile vendors; informal markets; formal markets; illegal markets; wholesale markets; people's markets; local markets; food courts; food hall; food enterprises; food MSMEs; food operators; market regulations; market operations; market management; market planning; licensing; license; business license; granting business license; franchise; urban planning; land use; retail infrastructures.	healthy food; market; supermarket; shopping mall; street vendors; vending machine; grocery store; food market; agricultural markets; traditional market; wet market; mobile vendors; local market; wholesale market; convenience store; canteen; school; school meals; children's meals; school nutrition; diet; hospital; army; prison; social welfare establishments; food supply; schools; hospitals; military; prison camps; social protection facilities; cooking staff; healthcare staff; nutrition officer; food services staff; industrial meals; nutritional meals for workers; worker meals.	thực phẩm lành mạnh; chợ; siêu thị; trung tâm thương mại; hàng rong; máy bán hàng tự động; cửa hàng thực phẩm; cửa hàng thực phẩm; chợ thực phẩm; chợ nông sản; chợ truyền thống; chợ bán sản phẩm tươi sống; cửa hàng lưu động; chợ dân sinh; chợ đầu mối; máy bán hàng tự động; cửa hàng tiện lợi; căng tin; trường học; bữa ăn học đường; bữa ăn cho trẻ; dinh dưỡng học đường; chế độ ăn; bệnh viện; quân đội; trại giam; cơ sở bảo trợ xã hội; máy bán hàng tự động; cung ứng thực phẩm; trường học; bệnh viện; quân đội; trại giam; cơ sở bảo trợ xã hội; máy bán hàng tự động; nhân viên nấu ăn; nhân viên y tế; cán bộ dinh dưỡng; nhân viên dịch vụ ăn uống; bữa ăn ca; bữa ăn dinh dưỡng cho người lao động; bữa ăn công nhân.
Food marketing	food labelling; food labeling; food label; nutritional information; nutrition facts; packaging; nutrition score; branding; trademark; certification; user manual; use instructions; storage instructions; food marketing; food promotion; food advertisement; food advertising; promotional information; branding; sponsorship; marketing exposure; marketing to children; nutrition knowledge; nutrition awareness; nutrition education; nutrition skills; cooking skills; food diversity; variety of food; diverse food; food groups; food preparation.	advertisement; promotion; not healthy; sugar drinkers; children; students, teenagers; breast milk substitutes; nutrition label; labeling; nutritional content; nutritional ingredients; required labeling; warning; health disclosure; front-of-pack label; simple label; label the menu board.	quảng cáo; khuyến mãi; không có lợi cho sức khỏe; đồ uống có đường; trẻ em; học sinh, thanh thiếu niên; sản phẩm thay thế sữa mẹ; nhãn dinh dưỡng; ghi nhãn; hàm lượng dinh dưỡng; thành phần dinh dưỡng; cảnh báo; công bố sức khỏe; nhãn mặt trước; nhãn đơn giản; dán nhãn bảng thực đơn.

Food desirability	food desirability; food acceptability; food preferences; food culture; consumer behavior; cooking traditions; cooking methods; traditional cuisine; local cuisine; consumer behavior; taste.	nutrition; diet guidelines; nutrition communication; propagate; raise awareness; nutrition information.	dinh dưỡng; hướng dẫn chế độ ăn; truyền thông dinh dưỡng; tuyên truyền; nâng cao nhận thức, thông tin dinh dưỡng.
Food prices and affordability	food prices; price stabilization; inflation; food taxes; food tax; agricultural tax; product tax; tax exemption; food subsidies; rice subsidies; rice support; salt subsidies; salt support; food assistance; food security; food affordability; purchasing power; safety nets; social protection; food programs; food security programs; food banks.	VAT; import tax; healthy products; special consumption tax; not healthy; sugar drinkers; subsidize; price stability; fertilizer support; variety support; free irrigation.	thuế GTGT; thuế nhập khẩu; sản phẩm có lợi cho sức khỏe; thuế tiêu thụ đặc biệt; không có lợi cho sức khỏe; đồ uống có đường; trợ cấp; bình ổn giá; hỗ trợ phân bón; hỗ trợ giống; miễn phí thủy lợi.
Food availability and accessibility	food accessibility; food availability; trade in agricultural products; Food Trade Agreement; SPS Agreement; Codex; OIE; IPPC; IFS; BRC; commercial policies; Free Trade Agreement; investment; investment incentives; domestic production; food import; food aid; storage; food flows; food proximity; food transportation; food access; access to food; healthy food supply; safe food supply; food deserts; food swamps.	grocery store; supermarket; fresh market; food retail outlets; distribution and consumption system; safe vegetable business; fresh food; organic agricultural products; food service store; restaurant; street food; product choice; healthy food; unhealthy food; fast food store; prohibited zones; around the school; wanted goods; opening hours limited; farmers markets; fruit and vegetable store; product consumption; available; supply fresh vegetables.	cửa hàng tiện lợi; siêu thị; chợ tươi sống; cửa hàng bán lẻ thực phẩm; hệ thống phân phối, tiêu thụ; kinh doanh rau an toàn; thực phẩm sạch; nông sản hữu cơ; cửa hàng dịch vụ ăn uống; nhà hàng; thức ăn đường phố; lựa chọn sản phẩm; thực phẩm lành mạnh; thực phẩm không lành mạnh; cửa hàng thức ăn nhanh; phân vùng cấm; xung quanh trường học; hàng rong; giới hạn giờ mở cửa; chợ nông sản; cửa hàng rau quả; tiêu thụ sản phẩm; sẵn có; cung cấp rau quả tươi.

4. Supp. Mat. #4: List of policy documents from which evidence was retrieved

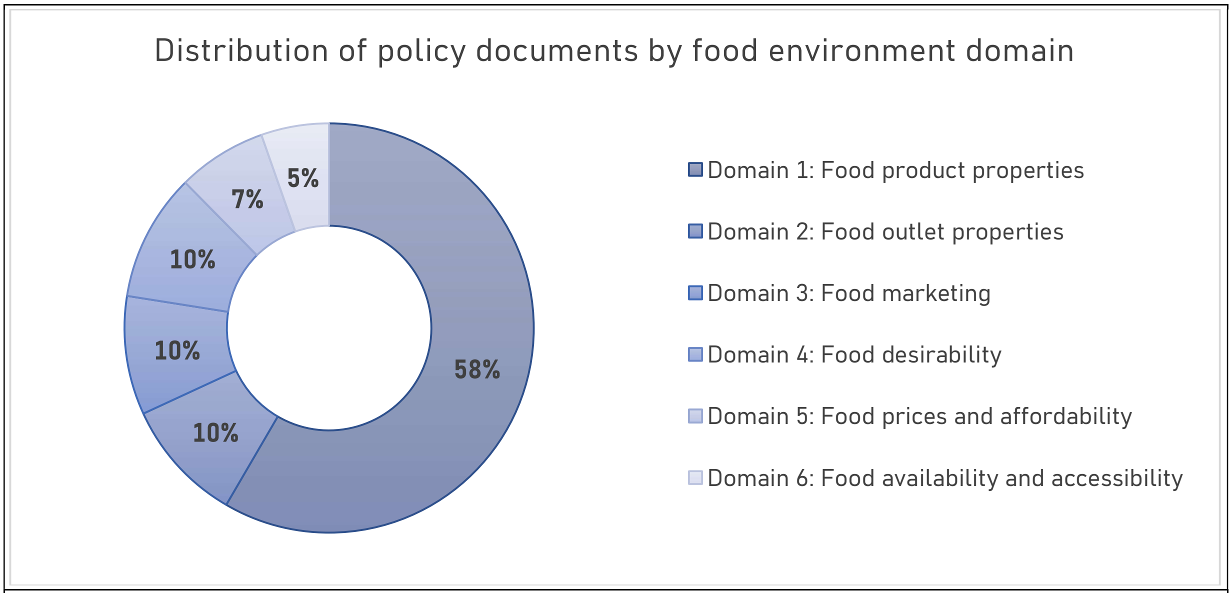

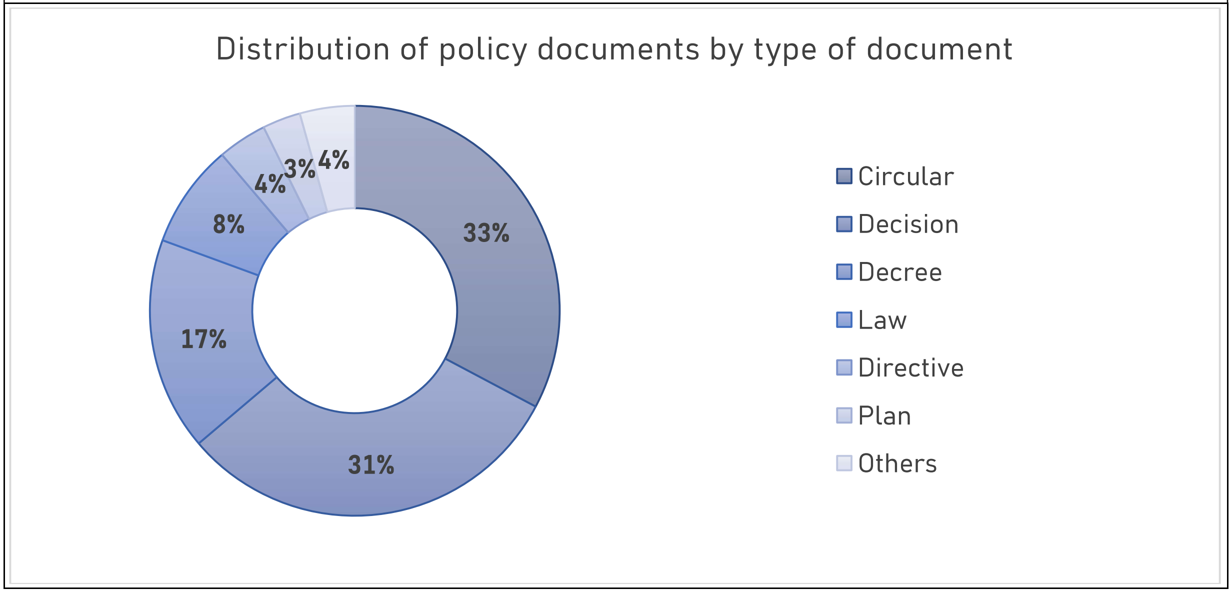

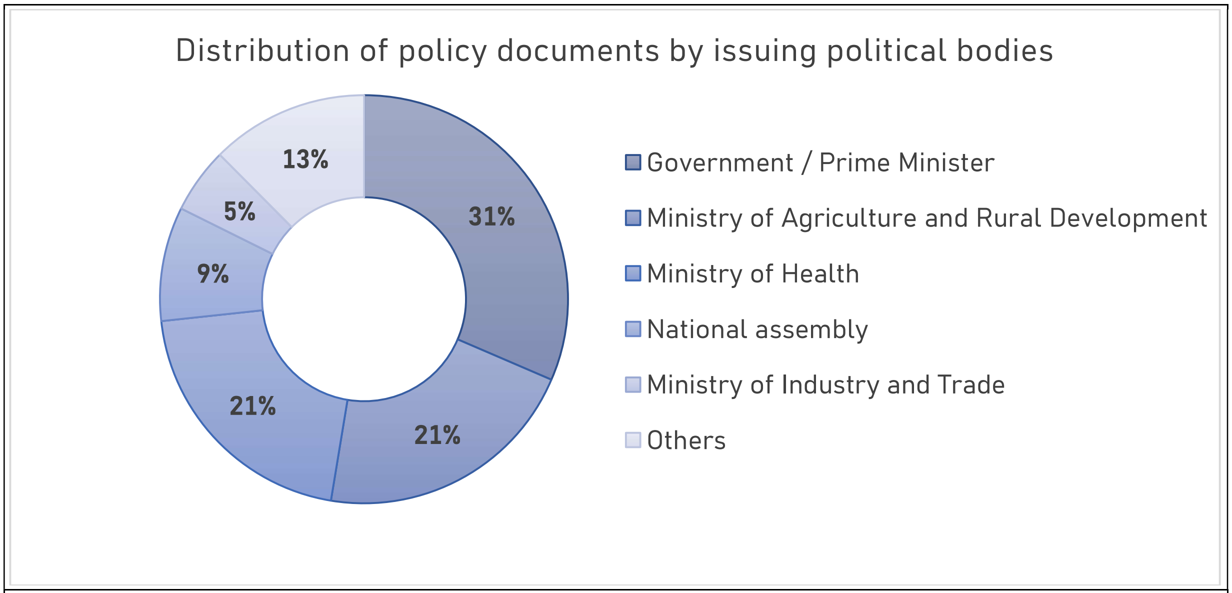
Distribution of policy documents by issuing political bodies
31%
21%
21%
9%
5%
13%
Government / Prime Minister
Ministry of Agriculture and Rural Development
Ministry of Health
National assembly
Ministry of Industry and Trade
Others

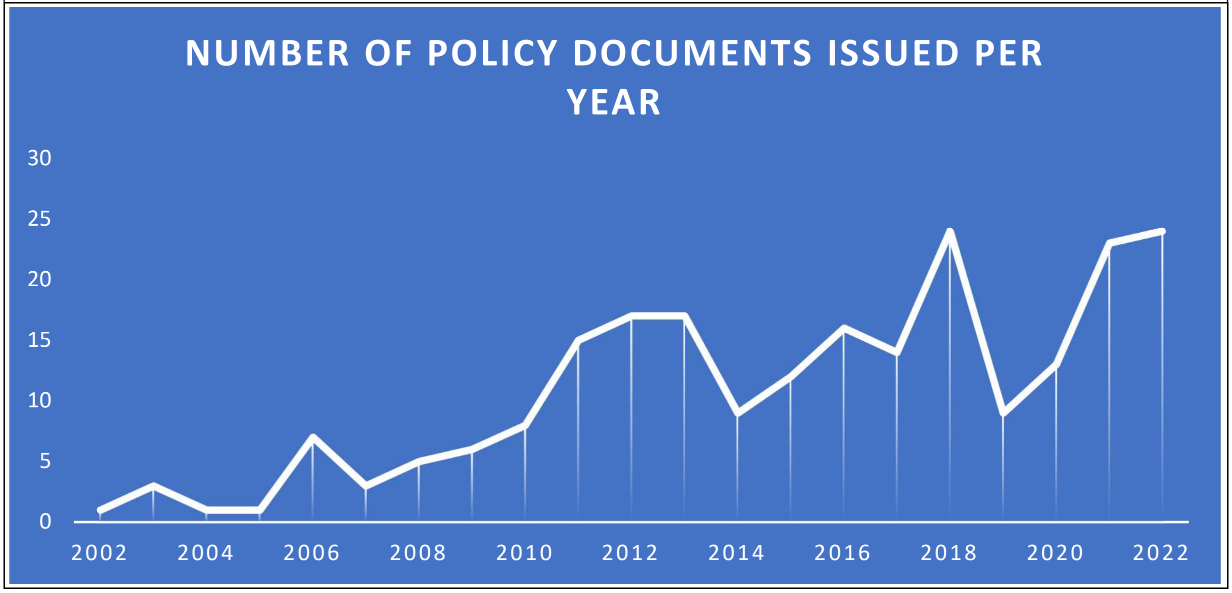
NUMBER OF POLICY DOCUMENTS ISSUED PER YEAR
30
25
20
15
10
5
0
2002
2004
2006
2008
2010
2012
2014
2016
2018
2020
2022

No.	Document type	Official document code	Policy name	Publishing body	Publication date
1	Law	36/2005/QH11	Law on Commercial	National assembly	2005
2	Law	68/2006/QH11	Law on Standards and Technical Regulations	National Assembly	2006
3	Law	05/2007/QH12	The Law on Product and Goods Quality	National assembly	2007
4	Law	13/2008/QH12	Law on value-added tax	National assembly	2008
5	Law	55/2010/QH 12	Law on Food safety	National assembly	2010
6	Law	11/2012/QH13	Law on Prices	National assembly	2012
7	Law	16/2012/QH13	Law on Advertising	National Assembly	2012
8	Law	15/2012/QH13	Law on Handling Administrative Violations	National Assembly	2012
9	Law	41/2013/QH13	Law on Plant protection and quarantine	National assembly	2013
10	Law	79/2015/QH13	Law on Veterinary Medicine	National assembly	2015
11	Law	106/2016/QH13	Amendments to some Articles of the Law on Value-added tax, the law on special excise duty, and the law on tax administration	National assembly	2016
12	Law	107_2016_QH13	The law export and import duties	National assembly	2016
13	Law	18/2017/QH14	Law on fisheries	National assembly	2017
14	Law	31/2018/QH14	Law on Crop production	National assembly	2018
15	Law	32/2018/QH14	Law on Animal husbandry	National assembly	2018
16	Law	02/VBHN-VPQH	Law on Food safety	National Assembly	2018
17	Law	47/VBHN-VPQH	Law on Advertising	National assembly	2018
18	Law	67/2020/QH14	Amendments and supplements to certain Articles of the Law on Handling of Administrative Violations	National Assembly	2020
19	Law	11/2022/QH15	Law on Inspection	National assembly	2022
20	Resolution	30a/2008/NQ-CP	The support program for fast and sustainable poverty reduction in 61 poor districts	Government	2008
21	Resolution	07c/NQ-BCH	Resolution of the 7th Conference of the Executive Committee of the Vietnam General Confederation of Labor (XI term) on the quality of shift meals of employees	Vietnam General Confederation of Labor	2016
22	Resolution	20-NQ/TW	Strengthen the work of protecting, caring for and improving people's health in the new situation	The central executive committee	2017
23	Resolution	53/NQ-CP	Solutions to encourage and promote businesses to invest in agriculture effectively, safely and sustainably promulgated by the Government	Government	2019
24	Resolution	34/NQ-CP	Ensuring National food security toward 2030	Government	2021
25	Resolution	101/2023/QH15	Resolution the 5th meeting of the 15th national assembly	National assembly	2023

26	Decree	58/2002/ND-CP	Promulgating the Plant Protection Regulation, the Plant Quarantine Regulation and the Plant Protection Drug Management Regulation	Government	2002
27	Decree	02/2003/NĐ-CP	Development and Management of marketplaces	Government	2003
28	Decree	127/2007/ND-CP	On detailing a number of articles of the Law on Standards and Technical Regulations	Government	2007
29	Decree	132/2008/ND-CP	Detailing the implementation of a number of articles of the Law on Product and Goods Quality	Government	2008
30	Decree	114/2009/ND-CP	Decree amending and supplementing a number of articles of the Government's Decree No. 02/2003/ND-CP dated January 14, 2003, on market development and management	Government	2009
31	Decree	34/2009/NQ-QH12	Stepping up the implementation of policies and Laws on food quality, hygiene and safety administration	National Assembly	2009
32	Decree	86/2011/ND-CP	Detailing and guiding the implementation of a number of articles of the Law on Inspection	Government	2011
33	Decree	07/2012/ND-CP	Providing for agencies assigned to perform the function of specialized inspection	Government	2012
34	Decree	178/2013/ND-CP	Regulations on sanctioning administrative violations in food safety	Government	2013
35	Decree	177/2013/NĐ-CP	Detailing and guiding the implementation of the Law on price	Government	2013
36	Decree	181/2013/ND-CP	Elaboration of some Article of the Law on Advertising	Government	2013
37	Decree	100/2014/ND-CP	On the trading in and use of nutritious products for infants, feeding bottles and teats	Government	2014
38	Decree	116/2014/ND-CP	Providing detailed instructions on the implementation of several articles of the Law on Plant Protection and Quarantine	Government	2014
39	Decree	122/2014/ND-CP	Organization and activities of the Health Inspectorate	Government	2014
40	Decree	127/2015/ND-CP	Organization and operation of inspectorates in Industry and Trade sector	Government	2015
41	Decree	09/2016/ND-CP	Providing for fortification of food with micronutrients	Government	2016
42	Decree	67/2016/ND-CP	Decree on requirements for food manufacturing and trading under specialized management of the Ministry of Health	Government	2016
43	Decree	35/2016/ND-CP	Guidelines for the law of veterinary medicine	Government	2016
44	Decree	43/2017/ND-CP	Goods labels	Government	2017
45	Decree	41/2017/ND-CP	Amendment and supplementation to a number of articles of the decree on administrative penalties in aquaculture, veterinary, animal breeds, animal feeds; forest management, development and protection, and forestry management	Government	2017
46	Decree	119/2017/ND-CP	On penalties for administrative violations against regulations on standards, measurement and quality of goods	Government	2017
47	Decree	109/2018/ND-CP	Organic agriculture.	Government	2018
48	Decree	115/2018/NĐ-CP	Penalties for administrative violations against regulations on food safety	Government	2018

49	Decree	15/2018/ND-CP	Elaborate some Articles of the Law of Food safety	Government	2018
50	Decree	78/2018/NĐ-CP	Amending and supplementing a number of articles of the Government's Decree No. 27/2007/ND-CP dated August 1, 2007, detailing the implementation of a number of articles of the Law on Standards and Technical Regulations	Government	2018
51	Decree	26/2019/ND-CP	Guidelines for implementation of the Law on Fisheries.	Government	2019
52	Decree	42/2019/ND-CP	Penalties for administrative violations agaisnt regulations on fisheries	Government	2019
53	Decree	13/2020/ND-CP	Elaborate the Law on Animal Husbandry	Government	2020
54	Decree	04/2020/ND-CP	Amendments to Government's Decree No. 31/2016/ND-CP dated May 06, 2016, on penalties for administrative violations against regulations on plant varieties, plant protection and quarantine, and Government's Decree No. 90/2017/ND-CP dated July 31, 20	Government	2020
55	Decree	98/2020/ND-CP	Prescribing penalties for administrative violations against regulations on commerce, production and trade in counterfeit and prohibited goods, and protection of consumer rights	Government	2020
56	Decree	111/2021/ND-CP	Amendments to some articles of Government's Decree No. 43/2017/ND-CP dated April 14, 2017, on goods labels	Government	2021
57	Decree	70/2021/ND-CP	Amending the Government's Decree No. 181/2013/ND-CP dated November 14, 2013, on elaboration of some Articles of the Law on Advertising	Government	2021
58	Decree	124/2021/ND-CP	Amending and supplementing a number of articles of the Government's Decree No. 115/2018/ND-CP dated September 4, 2018, on sanctioning of administrative violations on food safety and Decree No. 117/2020/ND -CP dated September 28, 2020, of the Government	Government	2021
59	Decree	38/2021/NĐ-CP	Regulations on sanctioning administrative violations in the field of culture and advertising	Government	2021
60	Decree	14/2021/ND-CP	Regulating penalties for administrative offences involving animal husbandry	Government	2021
61	Decree	126/2021/ND-CP	On amendments to certain articles of Decrees on administrative penalties in industrial property; standards, measurement and quality of goods; science and technology activities, technology transfers; atomic energy.	Government	2021
62	Decree	129/2022/NĐ-CP	Vietnam's special preferential import tariff schedule for implementation of the Regional Comprehensive Economic Partnership in the 2022 – 2027 period.	Government	2022
63	Decree	07/VBHN-BCT	Organization and operation of inspectorates in Industry and Trade sector	MOIT	2023
64	Decision	1233/2003/QD-BYT	Transferring the management of the Testing Center of Food Safety and Hygiene under the Department for Management of Food Hygiene and Safety to the National Institute of Nutrition	MOH	2003
65	Decision	5327/2003/QD-BYT	Promulgates the Regulation on taking food samples and specimens when food poisoning occurs	MOH	2003

66	Decision	1371/2004/QD-BTM	The Regulation on Department Stores and Trade	MOIT	2004
67	Decision	146-2006-QD-UB	Promulgating the "Mechanism to encourage investment in the construction of trade centers and supermarkets in Hanoi City" issued by the People's Committee of Hanoi	Government	2006
68	Decision	2879/QD/BYT	Approving The guidelines for the dietary in hospitals	MARD	2006
69	Decision	222/2006/QĐ-UBND	Promulgating “Regulations on encouraging investment and trading in safe vegetables and clean food in Hanoi city”	Hanoi People's Committee	2006
70	Decision	394/QD-TTg	To encourage investment in new construction and expansion of slaughterhouses, livestock and poultry preservation and processing establishments, and concentrated and industrial poultry breeding establishments.	Government	2006
71	Decision	39/2006/QD-BYT	Statute for food poisoning investigation	MOH	2006
72	Decision	146/2006/QD-UB	Mechanism to encourage investment in building commercial centers and supermarkets in Hanoi City	Hanoi People's Committee	2006
73	Decision	23/2007/QD-BNN	List of pesticides allowed to use, restricted from use, banned from use in Vietnam	MARD	2007
74	Decision	147/2008/QD-TTg	Approving the national action plan on acceleration of the implementation of commitments under the Agreement on the application of sanitary and phytosanitary measures in performing the WTO member obligations.	Government	2008
75	Decision	2374/QD-BNN-QLCL	Promulgating the Project on strengthening the quality management of agro-forestry-fishery products to ensure food hygiene and safety until 2015, with orientation to 2020	MARD	2009
76	Decision	641/QD-TTg	Decision on Approving the master plan on the development of Vietnamese' physical and stature in the 2011-2030 period	Government	2011
77	Decision	20/QD-TTg	Approving the National Strategy for Food Safety in the 2011-2020 period and the Vision 2030	Government	2012
78	Decision	226/QĐ-TTg	Ratification of the National Nutrition Strategy for 2011 – 2020, With a Vision toward 2030	Government	2012
79	Decision	01/2012/QĐ-TTg	Some policies supporting the application of the method of good agricultural practices to agriculture, silviculture and aquaculture	Government	2012
80	Decision	1228/QĐ-TTg	Approving the National Target Program on Food Hygiene and Safety for the period 2012 - 2015 promulgated by the Government	Government	2012
81	Decision	316/QD-BNN-HTQT	Approving the project "Strengthening the capacity of the agricultural and aquatic food safety control system"	MARD	2012
82	Decision	1555/QĐ-TTg	National action program for children for the period 2012 - 2020	Government	2012
83	Decision	189/QD-BYT	Promulgating “the 10 reasonable nutrition tips to 2020" to implement the National Strategy on Nutrition for the period 2011-2020 with a vision to 2030	MOH	2013

84	Decision	9762/QĐ-BCT	Project "Commercial development planning in the Southeast region to 2020, orientation to 2030"	MOIT	2013
85	Decision	3073/QĐ-BNN-QLCL	Approving the project to build and develop a model of a safe agro-forestry-fishery food supply chain nationwide	MARD	2013
86	Decision	2413/QĐ_TTG	Approving the project list "Strengthening capacity for information, education and communication on food safety to support the implementation of the Law on Food Safety and the National Strategy on Food Safety for the period 2011-2020 with a vision to	Government	2013
87	Decision	122/QD-TTg	Approving the national strategy to protect, care, and improve public health during 2011 - 2020 period, and the orientation towards 2030	Government	2013
88	Decision	4950/QD-BYT	Promulgating the plan for implementation the Government Decree No. 100/2014/ND-CP dated 6-Nov-2014 on "The trading in and use of nutritious products for infants, feeding bottles and teats"	MOH	2014
89	Decision	6481/QD-BCT	Master plan on development of a nationwide market network by 2025, vision to 2035	MOIT	2015
90	Decision	272/QĐ-BCT	Approving the Project on commercial development planning in the Red River Delta until 2020, with a vision to 2030, issued by the Minister of Industry and Trade	MOIT	2015
91	Decision	376/QD-TTg	Approval of the national strategy for preventing and controlling cancers, cardiovascular disease, diabetes, chronic obstructive pulmonary disease, bronchial asthma and other noninfectious diseases during the 2015-2025 period	Government	2015
92	Decision	38/2015/QĐ-TTg	pilot implementation of specialized inspection of food safety in districts, towns and cities, communes and wards, communes and towns of Hanoi and Ho Chi Minh City.	Government	2015
93	Decision	3800 /QD-BYT	Approving the guidelines for intervention to prevent stunting in children under 5 years to implement the national target program on construction new rural period 2016-2020	MOH	2016
94	Decision	4487/QD-BYT	Guidelines for the diagnosis and treatment of acute malnutrition in children aged 0 to 72 months.	MOH	2016
95	Decision	4988/QD-BYT	04 types of food safety inspection	MOH	2016
96	Decision	3705/QD-BCT	Supplementing wholesale markets, grade I markets under the "Master plan on development of the national market network up to 2025, with a vision to 2035"	MOIT	2017
97	Decision	1714/QD-TTg	Approving and signing the Memorandum of Understanding on Strengthening Cooperation in the field of Food Safety and Phytosanitary (SPS) between ASEAN Member States and China	MARD	2017
98	Decision	196/QD-BGDĐT	The balanced menu planning software	MOET	2017

99	Decision	1246/QĐ-BYT	Issue Instructions for implementation of three-step practice and storage of food samples for food service businesses	MOH	2017
100	Decision	38/2018/TT-BNNPTNT	Prescribing assessment and certification of compliance with food safety regulations of agro-forestry-fishery food manufacturing and trading businesses	MARD	2018
101	Decision	712/QD-TTg	National action program on zero hunger to 2025	Government	2018
102	Decision	3489/QĐ-BNN-QLCL	Assign the task of state inspection of imported food safety for products under the management of the Ministry of Agriculture and Rural Development to the relevant agencies.	MARD	2018
103	Decision	1092/QD-TTg	Approval of Vietnam Health Plan	Government	2018
104	Decision	2033/QD-BYT	National plan for communication and advocacy to reduce salt in the diet for prevention and control of hypertension, stroke and other non-communicable diseases, period 2018-2025	MOH	2018
105	Decision	47/2018/QĐ-TTg	Pilot implementation of specialized food safety inspection in districts, urban districts, towns, provincial cities and communes, wards and townships in districts, urban districts, towns and cities) cities of 09 provinces and centrally run cities: Han	Government	2018
106	Decision	41/QĐ-TTg	Approving scheme for proper nutrition assurance and physical activity increase for children and students with the aim of health improvement and prevention of cancer, cardiovascular disease, diabetes, chronic obstructive pulmonary disease and bronchia	Government	2019
107	Decision	533/QD-BNN-QLCL	Action plan to ensure food safety in the agricultural sector in 2019	MARD	2019
108	Decision	1896/QD-TTg	Promulgating program for nutrition care in the first 1.000 days of life for prevention of maternal and child undernutrition and improvement of Vietnamese people’s height	Government	2019
109	Decision	885/QD-TTg	The Organic Agriculture Development Project 2020 - 2030	Government	2020
110	Decision	3081/QD-BYT	Promulgates "Regulations on reporting regime and reporting form on food safety under the management of health sector"	MOH	2020
111	Decision	645/QD-TTg	Approval of national electronic commerce master plan during 2021 – 2025	Government	2020
112	Decision	5924/QD-TTg	Approving the implementation plan of the Vietnam health program in the 2021-2025 period	MOH	2021
113	Decision	1034/QD-BTTTT	The plan to support agricultural production households on the e-commerce platform, ending the development of the agricultural and rural digital economy	MIC	2021
114	Decision	184/QĐ-BNN-QLCL	Action plan to ensure food safety in the agricultural sector in 2021	MARD	2021
115	Decision	1163/QD-TTg	National strategy on domestic trade development 2021-2030, vision to 2045	Government	2021

116	Decision	3000/QD-BGDĐT	Approving the model of school meals to ensure reasonable nutrition combined with increased physical activity for Vietnamese children, pupils and students	MOET	2021
117	Decision	1956/QD-TTg	Signing of a Memorandum of Understanding between the Governments of the Member States of the Association of Southeast Asian Nations and the Government of the People's Republic of China on strengthening cooperation in the field of Food Safety and Phyt	Government	2021
118	Decision	1408/QD-TTg	Approval of the project on development of seafood processing industry in the period of 2021-2030	Government	2021
119	Decision	1660_QD_TTg	School Health Program for the period of 2021 - 2025	Government	2021
120	Decision	1660/QD-TTg	School Health Program for the period of 2021 - 2025	Government	2021
121	Decision	38/QD-TTg	The Project on “Reform of quality and food safety inspection model for imported goods”	Government	2021
122	Decision	1294/QD-BYT	Issue an action plan to implement the national strategy on nutrition until 2025	MOH	2022
123	Decision	02/QD-TTg	Approving the national nutrition strategy for the 2021-2030 period with a vision toward 2045	Government	2022
124	Decision	350/QD-BTTTT	The plan to support agricultural production households on the e-commerce platform, promote the development of the agricultural and rural digital economy in 2022	MIC	2022
125	Decision	56/QD-BTTTT	The plan to support agricultural production households on the e-commerce platform, promote the development of the agricultural and rural digital economy in 2022	MIC	2022
126	Decision	1099/QĐ-BNN-QLCL	Promulgating a plan to ensure food safety and improve the quality of agriculture, forestry and fishery in the agricultural sector in 2022	MARD	2022
127	Decision	1768/QD-BYT	Guidelines for the implementation of the content "Improving nutrition" under the National Target Program for Sustainable Poverty Reduction in the 2021-2025 period	MOH	2022
128	Decision	1858/QD-BYT	Guidelines for the implementation of interventions to prevent stunting in children under 5 years old to implement the National Target Program on building new rural for the period 2021-2025	MOH	2022
129	Decision	2195/QD-BGDĐT	Approving Guidelines for organizing school meals in combination with increasing physical activity for children and students in preschool and primary education institutions	MOET	2022
130	Decision	4280/QD-BGDĐT	Approving the Guidelines for organizing school meals combined with increased physical activity for junior high school students	MOET	2022
131	Decision	1088/QD-BNN-KTHT	Pilot project to build areas of agricultural and forestry raw materials meeting standards for domestic consumption and export in the period of 2022-2025	MARD	2022

132	Decision	2415_QD_BYT	Guidelines for the implementation of Project 7- Taking care of people's health, improving the physical condition and stature of ethnic minorities; child malnutrition prevention under the National Target Program on socio-economic development in ethnic	MOH	2022
133	Decision	155/QD-TTg	The National Plan for prevention and control of non-communicable diseases and mental disorders for the period of 2022-2025	Government	2022
134	Decision	300/QD-TTg	National action plan to transform transparent, responsible and sustainable food system in Vietnam by 2030	Government	2023
135	Decision	1588/QĐ-BNN-TCCB	Regulations on functions, tasks, powers and organizational structure of the National Agro-Forestry-Fisheries Quality Assurance Department	MARD	2023
136	Joint circular	08/2008/TTLT-BYT-BGDĐT ¶	Guiding for food safety assurance in educational institutions	MOET, MOH	2008
137	Circular	47/2009/TT-BNNPTNT	Promulgation of national technical regulations on food safety conditions applied to fish production	MARD	2009
138	Circular	82/2009/TT-BNNPTNT	Promulgation of national technical regulations of food hygiene and safety conditions in fisheries production	MARD	2009
139	Circular	75/2009/TT-BNNPTNT	Promulgating national technical regulations on food safety and hygiene conditions in agricultural production	MARD	2009
140	Circular	14/2010/TT-BYT	Promulgating national technical regulation on substances that may be added for zinc fortification in food	MOH	2010
141	Circular	32/2010/TT-BYT	National technical regulation on cheese products	MOH	2010
142	Circular	33/2010/TT-BYT	Promulgating national technical regulation for dairy fat products	MOH	2010
143	Circular	34/2010/TT-BYT	Promulgation of national technical regulation on natural water and bottled drinking water issued by the minister of health	MOH	2010
144	Circular	35/2010/TT-BYT	Promulgating national technical regulation for soft drinks	MOH	2010
145	Circular	45/2010/TT-BYT	Promulgation of the national technical regulation for alcoholic beverages	MOH	2010
146	Circular	37/2010/TT-BNNPTNT	Promulgating national technical regulations on food safety criteria for agricultural products	MARD	2010
147	Circular	34/2011/TT-BYT	Promulgating national technical regulations on containers and packaging in direct contact with food	MOH	2011
148	Circular	03/2011/TT-BYT	Promulgation of National Technical Regulations for Substances Used to fortify micronutrients to food	MOH	2011
149	Circular	18/2011/TT-BYT	Promulgation of national technical regulations for the micronutrient fortified food	MOH	2011
150	Circular	33/2011/TT-BNNPTNT	National technical regulation on veterinary hygiene conditions	MARD	2011
151	Circular	41/2010/TT-BYT	National technical regulation for fermented milk products	MOH	2011
152	Circular	31/2010/TT-BYT	National technical regulation for powdered milk products	MOH	2011
153	Circular	05/2011/TT-BYT	National technical regulations for food supplements with micronutrients	MOH	2011

154	Circular	76/2011/TT-BNNPTNT	Regulations on the list of foods permitted to be irradiated and the maximum allowable absorption dose for foods under the management of the Ministry of Agriculture and Rural Development	MARD	2011
155	Circular	01/2011/TT-BYT	Promulgation of national technical regulations on food additive	MOH	2011
156	Circular	02/2011/TT-BYT	Issuance of National Technical Regulations for limits of chemicals in food	MOH	2011
157	Circular	17/2011/TT-BYT	Provisions on radioactive contamination limit levels in foods	MOH	2011
158	Circular	13/2011/TT-BYT	Decentralization of tasks, testing indicators and testing procedures for state management in terms of food quality, safety and hygiene in the health sector	MOH	2011
159	Circular	20/2012/TT-BYT	Promulgating the national technical regulation on infant formula (for children up to 12 months of age)	MOH	2012
160	Circular	21/2012/TT-BYT	Issuing national technical regulation on formulas for special medical purposes intended for infants (up to 12 months of age)	MOH	2012
161	Circular	22/2012/TT-BYT	Issuing national technical regulation on follow-up formulas for infants from 6th month on and young children up to 36 months of age	MOH	2012
162	Circular	23/2012/TT-BYT	Issuing national technical regulations on processed cereal-based foods for infants from 6th month on and young children up to 36 months of age	MOH	2012
163	Circular	05/2012/TT-BYT	Promulgating the national technical regulations on microbiological contaminants in food	MOH	2012
164	Circular	02/2012/TT-BNNPTNT	Promulgating National Technical Regulations on food safety assurance conditions for establishments producing fish sauce, fishery products in the form of fish sauce and dried aquatic products used as food	MARD	2012
165	Circular	26/2012/TT-BKHCN	Stipulating the state inspection of quality of goods circulating in the market	MOST	2012
166	Circular	24/2013/TT-BYT	the Regulation on Maximum limits on residues of veterinary medicines in food	MOH	2013
167	Circular	48/2013/TT-BNNPTNT	Regulations on inspection and certification of aquatic food safety for export.	MARD	2013
168	Circular	07/2013/TT-BNNPTNT	Issuing the national technical regulation on fresh vegetable, fruit and tea - conditions for ensuring food safety in production and preparation	MARD	2013
169	Circular	40/2013/TT-BCT	Provisions on conditions, order and procedures for nominations of food safety testing facilities and responsibilities for state management of the Ministry of Industry and Trade	MOIT	2013
170	Circular	45/2012/TT-BCT	Regulations on inspection of food quality and safety during food production under the state management responsibility of the Ministry of Industry and Trade	MOIT	2013
171	Circular	02/2013/TT-BNNPTNT	Risk analysis and food safety management in the agro—forestry-fishery and salt production/trading chain	MARD	2013
172	Joint circular	20/2013/TTLT-BYT-BCT-BNNPTNT	Regulations on conditions, order and procedures for appointing food testing establishments in service of state management	MOH, MOIT, MARD	2013

173	Circular	16/2014/TT-BNNPTNT	National Technical Regulation on Quarantine and Plant Protection	MARD	2014
174	Circular	43/2014/TT-BYT	Regulating the management of functional foods	MOH	2014
175	Circular	30/2014/TT-BNNPTNT	Promulgating the list of plant quarantine articles; the list of plant quarantine articles subject to the pest risk analysis before being imported into Vietnam	MARD	2014
176	Circular	02/2014/TT-BNNPTTN	Regulating the order and procedures for issuing and revoking the certificate of genetically modified plants eligible for use as food and feed	MARD	2014
177	Joint circular	45/2015/TTLT-BNNPTNT-BKHCN	The labeling of prepacked genetically modified foods.	MARD, MOST	2015
178	Circular	48/2015/TT-BYT	Regulation on food safety inspection in food production and trading under the administration of the Ministry of Health	MOH	2015
179	Circular	31/2015/TT-BNNPTNT	The Circular on monitoring of toxic residue in farmed fisheries and fishery products.	MARD	2015
180	Circular	16/2015/TT-BNNPTNT	National technical regulations on aquaculture requirements	MARD	2015
181	Circular	52/2015/TT-BYT	Regulations on state inspection of food safety for imported food and dossiers and procedures for issuing certificates for exported food under the management of the Ministry of Health	MOH	2015
182	Circular	33/2015/TT-BNNPTNT	Regulations on hygiene and food safety supervision in the harvesting of bivalve mollusks	MARD	2015
183	Circular	50/2016/TT-BYT	Regulations on maximum residue levels of pesticide in food.	MOH	2016
184	Circular	08/2016/TT-BNNPTNT	On food safety supervision of agro-aqua-forestry products	Government	2016
185	Circular	09/2016/TT-BNNPTNT	Providing for animal slaughter control and veterinary hygiene inspection	MARD	2016
186	Circular	26/2016/TT-BNNPTNT	Providing for the quarantine of aquatic animals and animal products	MARD	2016
187	Circular	2956/CT-BNN-QLCL	Strengthening control over the abuse of chemicals and food additives in the production and trading of agro-forestry-fishery products	MARD	2016
188	Circular	07/2017/TT-BNNPTNT	The National Technical Regulation on “Fishery product - Frozen Tra fish fillets”	MARD	2017
189	Circular	40/2017/TT-BYT	Regulations on hygiene and safety standards for food, drinking water and meals of seafarers working on board Vietnamese ships	MOH	2017
190	Circular	03/2017/TT-BNNPTNT	Promulgating national technical regulations on requirements to ensure food safety in aquatic product production and business	MARD	2017
191	Circular	12/2017/TT-BKHCN	amending and supplementing a number of articles of the Circular No. 26/2012/TT-BKHCN dated December 12, 2012, of the Minister of Science and Technology providing for the state inspection of the quality of goods circulating on the market.	MOST	2017
192	Circular	11/2018/TT-BNNPTNT	Promulgating "National technical regulation on wholesale markets, agro-forestry-fishery auction markets - Food safety assurance requirements"	MARD	2018
193	Circular	23/2018/TT-BYT	Regulation on recall and disposal of disqualified foods under the management of the Ministry of Health	MOH	2018

194	Circular	36/2018/TT-BNNPTNT	Amendments to the Circular 26/2016/TT-BNNPTNT on quarantine of aquatic animals and products	MARD	2018
195	Circular	10/2018/TT-BNNPTNT	Promulgation of National Technical Regulation on veterinary drug-general requirements	MARD	2018
196	Circular	17/2018/TT-BNNPTNT	Regulations on methods of managing food safety conditions for agro-forestry-fishery production and business establishments that are not subject to the issuance of a Certificate of eligibility for food safety under the management of the Ministry of Ag	MARD	2018
197	Circular	07/2018/TT-BNNPTNT	Regulations on inspection of impurities in shrimp and shrimp products	MARD	2018
198	Circular	43/2018/TT-BCT	Stipulating food safety management by the Ministry of Industry and Trade	MOIT	2018
199	Circular	24/2019/TT-BYT	Guiding the management and use of food additives	MOH	2019
200	Circular	25/2019/TT-BYT	Traceability of food-related products	MOH	2019
201	Circular	08/2019/TT-BNNPTNT	National technical regulation on aquaculture feed.	MARD	2019
202	Circular	04/2020/TT-BNNPTNT	Promulgation of National technical regulation on animal feed and ingredients in aquaculture feed	MARD	2020
203	Circular	08/2020/TT-BNNPTNT	Amending and supplementing Article 2 of Circular No. 04/2020/TT-BNNPTNT dated March 9, 2020, of the Minister of Agriculture and Rural Development promulgating the National Technical Regulation on animal feed and raw materials aquatic feed production	MARD	2020
204	Circular	27/2020/TT-BCT	Provisions on contents, order and procedures for inspection, handling of administrative violations and implementation of professional measures of market management force	MOIT	2020
205	Circular	03/2020/TT-BNNPTNT	The National technical regulation on environmental treating products in aquaculture	MARD	2020
206	Circular	67/2021/TT-BTC	Providing for fees for food safety and hygiene affairs and the collection, transfer, management and use thereof	MOF	2021
207	Circular	555/QD-BNN-TT	The scheme for restructuring of Vietnam's rice industry by 2025 and 2030	MARD	2021
208	Circular	28/2021/TT-BYT	Promulgated the nomenclature of foods, food additives and food implements, packaging and containers with a view to state management of safety of imported foods	MOH	2021
209	Circular	17/2021/TT-BNNPTNT	Regulation on origin tracing, recall, and handling of unsafe food under the management of The Ministry of Agriculture and Rural Development	MARD	2021
210	Circular	20/2021/TT-BCT	Amendments and supplements to a number of articles of Circular No. 27/2020/TT-BCT	MOIT	2021
211	Circular	19/2022/TT-BNNPTNT	Promulgating lists of approved and prohibited plant protection chemicals in Vietnam	MARD	2022
212	Circular	32/2022/TT-BNNPTNT	Amending and supplementing a number of Circulars stipulating the appraisal and certification of agricultural, forestry and aquatic food production and	MARD	2022

			trading establishments meeting the conditions for ensuring food safety under the management of the		
213	Circular	06/2022/TT-BNNPTNT	https://thuvienphapluat.vn/van-ban/The-thao-Y-te/Thong-tu-06-2022-TT-BNNPTNT-sua-doi-Thong-tu-kiem-dich-dong-vat-san-pham-dong-vat-thuy-san-523855.aspx	MARD	2022
214	Circular	16/2021/TT-BNNPTNT	List of potentially unsafe products and goods under the management responsibility of the Ministry of Agriculture and Rural Development	MARD	2022
215	Directive	08-CT/TW	Strengthening the Party's leadership in food safety management; improve capacity and quality of state management of food safety; promote advocacy, propaganda and education, create a real change in food safety behavior, promote socialization of food s	Secretariat	2011
216	Directive	4462 /BYT-ATTP	on strengthening measures to ensure food safety, prevent food poisoning and food-borne diseases issued by the Ministry of Health	MOH	2013
217	Directive	34/CT-TTg	On continuing to promote food safety assurance and food poisoning prevention in the new situation	Government	2014
218	Directive	08/CT-UBND	Strengthening management of food service and street food business activities in Hanoi	Hanoi People's Committee	2016
219	Directive	13/CT-TTg	Strengthening state management of food safety	Government	2016
220	Directive	46/CT-TTg	Enhancement of Nutrition in new circumstances	Government	2017
221	Directive	584/BGDĐT-GDTC	Strengthening nutrition work in the new situation issued by the Ministry of Education and Training	MOET	2018
222	Directive	17/CT-TTg	Continue to strengthen the responsibility of state management of food safety in the new situation	Government	2020
223	Directive	17-CT/TW	Directive on enhancing security and food safety in the new situation	The central executive committee	2022
224	Decree	04/VBHN-BKHCN	Detailing the implementation of a number of articles of the Law on Standards and Technical Regulations	MOST	2018
225	Plan	251/KH-UBND	Plan: Food safety work in Hanoi city in 2021	Hanoi People's Committee	2020
226	Plan	41/KH-UBND	Action plan: Ensuring food safety in the agricultural sector in 2021	Hanoi People's Committee	2021
227	Plan	122/KH-UBND	Hanoi Plan on installation of automatic vending machines by 2025	Hanoi People's Committee	2022
228	Plan	375/KH-BCĐTUATTP	Launching "Action Month for Food Safety"	Central Inter-Sectoral Steering Committee on Food Safety	2022
229	Plan	18/KH-UBND	Implement the project "Management of food production and trading establishments in markets in Hanoi city in the period of 2022-2025"	Hanoi People's Committee	2022

230	Plan	336/KH-UBND	Food safety work plan in Hanoi city in 2023	Hanoi People's Committee	2022
231	Plan	266/KH-UBND	Plan: Implement the National Strategy on Nutrition in Hanoi, period 2022-2030	Hanoi People's Committee	2022
232	Documentary	3109/BCT-KHCN	To the Departments of Industry and Trade of provinces and cities guiding the implementation of Decree No. 15/2018/ND-CP.	MOIT	2018

5. Supp. Mat. #5: Evidence report

Food product properties

Food safety

Indicators	Definitions and scope	Evidence of Vietnamese Government actions	International Best Practice Examples (Benchmarks)	Final Score and Justification
**IND1.1.1** Food laws and regulations have been established for ensuring food safety	Any policy documents regulating all related matters of food safety to ensure that food consumed, produced, distributed, or marketed in the country meets the highest standards of food safety and hygiene reasonably attainable and to ensure that food complies with legal requirements, or where appropriate with recognized codes of good practice including regulation on enforcement of all food	Law 55/2010/QH12 (*Law on Food safety*), issued by The National Assembly in 2010, defines mandatory technical regulations for food safety. It affirms that organizations and individuals participating in the food chain are obliged to ensure food safety. It provides procedures for food testing and inspection, and food safety risk analysis.<br><br>The decree 15/2018/ND-CP (*Elaborate some Articles of the Law of Food safety*), issued by the Government in 2018, specifies the procedures of certification and declaration of conformity with technical regulations, food safety controls for Genetically Modified Organisms (GMOs), state control of imported and exported foodstuffs, food labeling, and advertising.<br><br>Additional laws contribute to the regulation of food safety in Vietnam, including:<br><br>The Law 68/2006/QH11 (*Law on Standards and Technical Regulations*), issued by the National Assembly in 2006, provides guidelines for the formulation, promulgation, and application of standards and technical regulations, as well as for the assessment of conformity with these standards and regulations.<br><br>The Law 05/2007/QH12 (*Law on Quality of Products and Commodities*), issued by the National Assembly in 2007, provides guidelines for the rights and obligations of individuals and organizations involved in the production and trade of products and goods, as well as those involved in activities related to product and goods quality. It establishes standards for product and goods quality, including requirements for labeling, packaging, and advertising, as well as methods for testing and inspection.<br><br>The Law 11/2022/QH15 (Law on inspection), issued by the National Assembly in 2022 (replacing The Law 56/2010/QH12 (*Law on inspection*), issued by the National Assembly in 2010, that expired on July 1, 2023), establishes a system for the management and implementation of inspections, including the creation of inspection plans, the selection	*Australia:* Food Act 2001 sets out food safety requirements for Australia. This Act requires food sold both local and imported in Australia is safe and suitable for human consumption and meets all standards set out in the Food Standards Code.	**A**<br><br>Vietnam is equipped with a modern food safety law and technical regulations encompassing production, handling, storage, and preparation of food. Food safety standards and guidelines have been established. The roles and responsibilities of the relevant authorities involved in ensuring food safety are clear.<br><br>We identified 384 policy documents related to technical regulations (cf. FS database), focusing on food safety assurance conditions for food production and trading, food safety assurance conditions for import and export, as well as standards settings.

	legislation in Vietnam.	of inspectors, and the issuance of inspection reports. It also outlines the rights and obligations of individuals and organizations subject to inspection, including the right to receive notice of the inspection and the right to provide feedback and suggestions.<br><br>In addition, in Law 79/2015/QH13 (Law on Veterinary Medicine); Law 32/2018/QH14 (Law on Livestock); Law 31/2018/QH14 (Cultivation Law); Law 18/2017/QH14 (Law on Fisheries); Law 41/2013/QH13 (Law on Plant Protection and Quarantine); Decree No. 127/2007/NDCP of the Government: Detailed regulations for the implementation of a number of articles of the Law on Standards and Technical Regulations; Decree No. 132/2008/ND-CP of the Government: Detailed regulations for the implementation of a number of articles of the Law on Product and Goods Quality; Decree No. 43/2017/ND-CP dated April 14, 2017 of the Government on goods labels and Decree No. 111/2021/ND-CP dated December 9, 2021 of the Government Amending and supplementing a number of Article Decree No. 43/2017/ND-CP dated April 14, 2017 of the Government on goods labels also has regulations on ensuring food safety.		
**IND1.1.2** A food **control management system** have been established for ensuring food safety	Mechanisms and tools are in place to implement, monitor and enforce food legislation in the country to ensure compliance with the food standards set out by the laws and regulations.	The Law 55/2010/QH12 (*Law on Food safety*), issued by the National Assembly in 2010, and Decree 15/2018/ND-CP (Detailing a number of articles of the Food Safety Law) delegates the state's responsibilities for food control and food safety management to the government, and reaffirms the key role the Ministry of Health (MOH), the Ministry of agriculture and Rural Development (MARD), and the Ministry of Industry and trade (MOIT). Management responsibilities are distributed among the 3 ministries based on the category of food at the stage of the value chain at stake.<br><br>Decision 4988/QD-BYT 2016 of the Ministry of Health promulgating 04 food safety inspection procedures, in which stipulating the process of scheduled inspection and unscheduled inspections of food safety.<br><br>Circular 48/2015/TT-BYT 2015 by the Ministry of Health regulating food safety inspection activities in food production and trading sectors managed by MOH which clearly states that the authorized organization must prepare the annual inspection plan in which deadlines are before November 1 for the commune level, before November 15 for the district level, before December 1 for provincial level and before December 15 for the Department of Food safety.<br><br>Circular 38/2018/TT-BNNPTNT (Regulating the appraisal and certification of agricultural, forestry and fishery food production and trading establishments that meet food safety conditions under the management of the Ministry of Agriculture and Rural Development) regulates Assessing and certifying food safety conditions for establishments producing and trading agricultural, forestry and fishery foods.	_Australia:_ Food Standards Australia New Zealand (FSANZ), a statutory authority in Australian Government Health portfolio develops and manages standards for food, called The Food Standards Code for Australia and New Zealand. The code contains standards and legal requirements for food safety that involve general food standards, food product	**B**<br><br>Beyond the assignment of responsibilities for state management of food safety, we identified 552 policy documents related to food safety control management, such as food safety inspection, food safety examination and supervision, food sampling, testing, and analysis, integrated risk management, prevention and remedy of food safety incidents, traceability, recall and disposal of unsafe foods, and regulations on penalties for food safety violations. However, specialized food safety inspection regulations have only been developed at the provincial/city level, and not yet developed for lower levels. The pilot program at the district and ward level has ended and is not maintained.

		Law 11/2022/QH15 (Law on Inspection); Decree No. 43/2023/ND-CP (Detailing a number of articles and measures to implement the inspection law) and Decree 07/2012/ND-CP (regulations on agencies assigned to perform the specialized inspection function and specialized inspection activities) of the Government in 2012 stipulating agencies assigned to perform specialized inspection functions; Duties and powers of agencies assigned to perform specialized inspection functions. Based on the functions and tasks of each ministry, the Government also issued specific regulations on tasks, powers, responsibilities, content and specialized inspection activities on food safety for the Ministry of Health, Ministry of Agriculture and Rural Development, Ministry of Industry and Trade according to Decree 122/2014/ND-CP on health inspection, Decree 47/2015/ND-CP on agricultural inspection, Consolidated document 7/VBHN-BCT on industry and trade inspection.<br><br>Law 67/2020/QH14 (Law amending and supplementing a number of articles of Laws 15/2012/QH13, 54/2014/QH13 and 18/2017/QH14 regulating the handling of administrative violations); Decree 115/2018/ND-CP (regulating penalties for administrative violations of food safety).<br><br>Decree No. 124/2021/ND-CP (Amending and supplementing a number of articles of Decree No. 115/2018/ND-CP dated September 4, 2018, of the Government stipulating penalties for administrative violations of food safety products) regulates administrative violations, forms, levels of sanctions, and authority to sanction administrative violations of food safety.<br><br>Directive 34/CT-TTg (On continuing to promote food safety assurance and food poisoning prevention in the new situation) dated December 11, 2014, of the Prime Minister; Directive 13/CT-TTg (Strengthening state management of food safety) issued by the Government in 2016 has provided specific solutions that authorities need to implement to strengthen inspection work. Inspect, inspect and monitor food safety in food production, processing, trading and distribution chains. At the same time, he emphasized the importance of inter-sectoral cooperation and coordination to ensure effective implementation of food safety regulations. Next, the Prime Minister issued Directive 17/CT-TTg dated April 13, 2020, on continuing to strengthen state management responsibility for food safety in the new situation. And most recently, Directive No. 17-CT/TW dated October 21, 2022, of the Secretariat on strengthening security and food safety in the new situation. One of the key tasks and solutions raised is: "Strengthening inspection, examination and supervision of food security and safety assurance; resolutely fight, prevent and strictly handle organizations, individuals, production and business establishments that violate regulations on security and food safety; proactively prevent and combat negative group interests in the field of security	standards, food safety standards, primary production, and processing standards. The Australian Government and state and territory governments enforce the standards, in line with their food legislation. The Department of Agriculture ensures that imported products meet the strict biosecurity laws and food standards.<br><br>*__Ireland:__* The Food Safety Authority of Ireland Act, 1998 contains enforcement provisions which are in addition to the powers to prosecute and other provisions on specific pieces of food legislation. The provisions in the FSAI Act, 1998 provide a means of reacting to and	

		and food safety. Promote the mass movement to detect and denounce violations of security and food safety; creating public opinion, consumers condemn and boycott products and goods that do not ensure food safety. Have adequate policies, timely rewards and appropriate measures to protect those who report violations of security and food safety. At the same time, strictly handle acts of taking advantage of security and food safety to lower reputation and negatively affect the healthy production and business of organizations, businesses and individuals.<br><br>Decision 20/QD-TTg (*approving the national food safety strategy for the 2011-2020 period, with a vision toward 2030*), issued by the Government in 2012, sets up the national food safety strategy and objectives. It aims at raising awareness and practicing food safety for target groups; building capacity for food safety management system; improving food safety assurance in manufacture, processing and selling facilities; actively preventing acute food poisoning.<br><br>Decision 38/QD-TTg (Approving the Project on Reforming the Quality Inspection and Food Safety Inspection Model for Imported Goods) dated January 12, 2021, of the Prime Minister to conduct comprehensive reform regulations on quality inspection and food safety inspection activities for imported goods to reduce costs and time for customs clearance of imported goods and reasonably protect domestic production, protect environment, rights and public health.<br><br>Decision 1588/QĐ-BNN-TCCB (Responsibilities and organizational structure of the NAFIQPM) issued on April 19, 2023, by MARD (replacing the expired Decision 670/QD-BNNTCCB), assigns the Department of Quality, Processing and Market Development (NAFIQPM) as the contact point in organizing the implementation of legislation on quality and safety of agroforestry and fishery products within MARD's authority.	dealing with situations posing a risk to public health. The Food Safety Authority of Ireland (FSAI) has the statutory responsibility for enforcing food legislation. Enforcement is carried out mainly through service contracts with official agencies who act on behalf of the FSAI.	
**IND1.1.3** A strategy and plan have been developed for information, education, communication and training on food safety.	Dissemination of food safety issues and legislation at the policy level to raise public awareness, improve knowledge, compliance, practices and implementation of food safety	The Law 55/2010/QH12 (*Law on Food safety*), issued by the Government in 2010, provides an overview of the objectives, requirements, contents and forms of information, education, and communication on food safety in Vietnam. It targets both organizations and individuals involved in food production and trading, as well as food consumers.<br><br>Resolution 43/2017/QH14 (on promoting the implementation of legal policies on food safety for the period 2011 - 2016) of the National Assembly in 2017 identifies one of the key tasks to be carried out, which is to strengthen the education, communication, accurate, timely, comprehensive, responsible, and honest information on issues related to food safety.	<u>*Ireland*</u>*:* The FSAI Act imposed Food Safety Training is a legal requirement that food handlers are supervised and instructed and/or trained in food hygiene matters commensurate with their work activity. The Food	**B**<br><br>Many existing policies contain communication programs and plans on food safety. There are also specific regulations on objectives, implementation plans, and specific assignment of responsibility for communication on food safety to relevant agencies.

	standard by different stakeholders in the food sectors.	Plan 375/KH-BCDTUFO (Deployment plan for the "Action Month for Food Safety" in 2022) of the Central Steering Committee on Food Safety in 2022 sets out goals, priority subjects and propaganda content, disseminates specific knowledge on food safety to each audience.<br><br>Decision 1228/QD-TTg (National Target Program on Food Hygiene and Safety for the period 2012 - 2015) of the Prime Minister in 2012 has a project on information, education and communication to ensure food hygiene and safety. In which, clearly specify the objectives, contents of activities, assignment of implementation and estimated budget for project implementation.<br><br>There are also communication plans and strategies on food safety such as Decision 184/QD-BNN-QLCL ("Action Plan to ensure food safety in the agricultural sector in 2021") issued by the Ministry of Agriculture and Rural Development in 2021, sets out the goals and tasks for the implementation of information and communication work on food quality and safety in the agricultural sector.<br><br>Decision 1384/QD-BNN-QLCL (Project "Ensuring food safety, improving the quality of agriculture, forestry and fishery in the 2021-2030 period") in 2022 of the Ministry of Agriculture and Rural Development stipulates propaganda activities, dissemination of laws and information and communication on the quality and safety of agro-forestry-fishery products.<br><br>Decision 2413/QD-TTg (list of projects "Strengthening capacity for information, education and communication on food safety to support the implementation of the Law on Food Safety and the National Strategy for Food Safety in the second phase) period 2011-2020 and vision 2030" funded by FAO) in 2013 specifying the implementing agency of the project; objectives, main results, and project implementation time.<br><br>Directive No. 34/-TTg dated December 11, 2014, of the Prime Minister on continuing to promote the work of ensuring food safety and preventing food poisoning in the new situation has launched. Organize Action Month on quality, hygiene and food safety.<br><br>Directive No. 17-CT/TW (on enhancing security and food safety in the new situation) dated October 21, 2022 of the Central Executive Committee has provided key tasks and solutions as: "The Vietnam Fatherland Front, socio-political organizations, Vietnam Cooperative Alliance, Consumer Protection Association and other organizations proactively and actively propagate, mobilize and provide information, consulting services, and legal support on ensuring food security and safety; Timely information	Safety Authority of Ireland has published a series of training guideline known as Guide to Food Safety Training for different skill levels.	Yet, there is no specific guidance on education and especially trainings on food safety for food system stakeholders

		about safe, high-quality food supply chains, violating establishments, potential risks of insecurity and food safety to union members, members and people; Strengthen the role and responsibility of monitoring and social criticism in the field of security and food safety."<br><br>The Directive 13/CT-TTg (*Strengthening state management of food safety*), issued by the Government in 2016, recognizes the importance of enhancing public awareness and education on food safety issues. It stipulates that press agencies operating at both central and local levels must intensify food safety coverage through diverse means such as news articles, columns, special pages, and programs. This coverage is intended to showcase examples of safe food production, processing, and circulation, as well as cases of food safety violations. Furthermore, broadcast media channels such as TV and radio are to work in close collaboration with the MOH, MARD and MOIT to increase public awareness and knowledge regarding food safety.		

Food nutritional quality

**Indicators**	**Definitions and scope**	**Evidence of Vietnamese Government actions**	**International Best Practice Examples (Benchmarks)**	**Final Score and Justification**
**IND1.2.1 Food composition targets/standards/restrictions** have been established for the content of the nutrients of concern (trans fats, added sugars, salt, saturated fat) in **industrially processed foods - SALT**	Includes packaged food manufactured in country our overseas and imported to Vietnam.<br><br>Includes mandatory or voluntary targets, standards (i.e. reduce by X%, maximum mg/g per 100g or per serving).<br><br>Include legislated ban on nutrients of concern.<br><br>Excludes legislated restrictions related to other ingredients (e.g., additives).<br><br>Excludes mandatory food composition regulation related to vitamins and	Decision 376/QD-TTg (*Approval of the national strategy for preventing and controlling cancers, cardiovascular disease, diabetes, chronic obstructive pulmonary disease, bronchial asthma and other non-infectious diseases during the 2015-2025 period*), issued by MOH in 2015, sets out objectives to manage and monitor salt, sugar, fat and additives in processed foods.<br><br>Objective is to reduce average consumption of salt by 30% between 2015 and 2025.<br><br>Decision 2033/QD-BYT (*National plan for communication and advocacy to reduce salt in the diet for prevention and control of hypertension, stroke and other non-communicable diseases, period 2018-2025*), issued by MOH in 2018, mentions the need to establish standards for the	<u>*South Africa*</u>: In 2013, the South African Department of Health adopted *mandatory targets for salt reduction in 13 food categories by means of regulation* (Foodstuffs, Cosmetics and Disinfectants Act).<br><br><u>*Argentina/South Africa*</u>: Laws on max levels of sodium in a broad range of food categories	**C**<br><br>The issue of high salt content in processed food is acknowledged. There are targets set to reduce salt consumption and to have 30% of businesses implement salt reduction measures. However, there are no standards specifying the maximum salt content of processed food, and there is no clarity regarding who is controlling salt content in processed food.

	micronutrients (e.g., iodine fortification).<br><br>Industrially processed foods are the processed and ultra-processed foods according to the NOVA classification (please find the complete definitions here: (https://world.openfoodfacts.org/nova).<br><br>Processed foods, such as pickled vegetables (dưa cà muối), canned fish, processed meat products (e.g., sausages, giò, chả, etc.,) cheeses and freshly made breads, are made essentially by adding salt, oil, sugar, or other substances from Group 2 (processed culinary ingredients) to Group 1 (unprocessed or minimally processed) foods.<br><br>Ultra-processed foods, such as soft drinks, sweet or savory packaged snacks, reconstituted meat products and pre-prepared frozen dishes, are not modified foods but formulations made mostly or entirely from substances derived from foods and additives, with little if any intact Group 1 (unprocessed or minimally processed foods) foods. The overall purpose of ultra-processing is to create branded, convenient (durable, ready to consume), attractive (hyper-palatable) and highly profitable (low-cost ingredients) food products designed to displace all other food groups	amount of salt included in pre-packaged foods. Additionally, the decision sets quantitative objectives for food processing companies, requiring that at least 30% of such businesses implement measures to reduce salt in cooking, processing, and food supply. It also mandates that over 30% of pre-packaged food production establishments have at least one product that is reduced in salt and labelled with a declaration of salt content, an indicator of foods with high salt content, and a health warning about overconsumption of salt. The decision further recommends that companies research and implement advanced technology and techniques to produce low-sodium salts, sauces, seasonings, and ready-made foods.<br><br>Circular 23/2012/TT-BYT (on national technical regulations for nutritional products made from cereals for children from 6 to 36 months of age) regulating salt content for nutritional products for children under 36 months old.		The maximum salt threshold indicated in Circular 23/2012/TT-BYT is twice as high than the WHO recommendation; also, the circular has not taken into account the updated WHO nutritional profile model on sugar, salt, and fat ingredients.
**IND1.2.1 Food composition targets/standards/restrictions** have been established for the	See definitions and scope above for **IND1.2.1**	Decision 376/QD-TTg (*Approval of the national strategy for preventing and controlling cancers, cardiovascular disease, diabetes, chronic*	*UK*: In 2016, a key commitment of the 'Childhood obesity:	**C**

content of the nutrients of concern (trans fats, added sugars, salt, saturated fat) in **industrially processed foods** - **SUGAR**		*obstructive pulmonary disease, bronchial asthma, and other non-infectious diseases during the 2015-2025 period*), issued by MOH in 2015, sets out objectives to manage and monitor salt, sugar, fat, and additives in processed foods.<br><br>Circular 23/2012/TT-BYT (*on national technical regulations for nutritional products made from cereals for children from 6 to 36 months of age)* regulating Sugar content for nutritional products for children under 36 months old.	a plan for action' was to launch a broad, structured sugar reduction program to remove sugar from everyday products. Reduction program to remove sugars by at least 20% by 2020.<br><br>*France*: Under a Charter of Engagement with the food industry (2008), companies can make voluntary commitments to reduce salt, sugar, total and saturated fats and increase fiber.	The issue of high sugar content in processed food is acknowledged; however, there are no specific targets, standards, or restrictions for limiting sugar content in processed food.
**IND1.2.1 Food composition targets/standards/restrictions** have been established for the content of the nutrients of concern (trans fats, added sugars, salt, saturated fat) **in industrially processed foods** – **FAT**	See definitions and scope above for **IND1.2.1**	Decision 376/QD-TTg (*Approval of the national strategy for preventing and controlling cancers, cardiovascular disease, diabetes, chronic obstructive pulmonary disease, bronchial asthma, and other non-infectious diseases during the 2015-2025 period*), issued by MOH in 2015, sets out objectives to manage and monitor salt, sugar, fat, and additives in processed foods.	*The Netherlands*: On January 2014, the Dutch Ministry of Health, Welfare and Sport signed an agreement with trade organisations representing food manufacturers, supermarkets, hotels, restaurants, caterers and the hospitality industry to lower the levels of salt, saturated fat and calories in food products.	**C**<br><br>The issue of fat content in processed food is acknowledged; however, there are no specific targets, standards, or restrictions for limiting trans-fat content in processed food.

**IND1.2.2 Food composition targets/standards/restrictions** have been established for the content of the nutrients of concern (trans fats, added sugars, salt, saturated fat) in **meals sold from food service outlets** - **SALT**	Meals sold at food service outlets include foods sold at quick service restaurants, dine-in restaurants and take-away food outlets, coffee, bakery, and snack/street food outlets (both fixed outlets and mobile food vendors). This also includes food from catering operations and delivery meals.<br><br>Includes legislated bans on nutrients of concern.<br><br>Includes mandatory or voluntary targets (i.e., reduce by X%, maximum mg/g per 100g or per serving).<br><br>Excludes legislated restrictions related to other ingredients (e.g., additives)<br><br>Excludes mandatory out-of-home meal composition regulations related to vitamins and micronutrients, e.g., folic acid or iodine fortification.<br><br>Excludes food consumption standards/targets for fiber, healthy ingredients like fruits and vegetables.<br><br>Excludes the provision of resources or expertise to support food service outlets with reformulation.	Decision 2195/QD-BGDĐT (*Approving Guidelines for organizing school meals in combination with increasing physical activity for children and students in preschool and primary education institutions*), issued by MOET in 2022, mentions the need to limit sugar and salt in school menus and requires taking measures to limit the sale of foods containing a lot of salt, sugar, and fat in school canteens. Standards for **salt** are set as following: "Primary school students should consume no more than 4g of salt per day, while children under 5 years old should consume no more than 3g per day. Spices and salt should not be added to the food of children under 1 year old. When preparing food, it is recommended to use iodized salt."<br><br>Decision 2033/QD-BYT (*National plan for communication and advocacy to reduce salt in the diet for prevention and control of hypertension, stroke and other non-communicable diseases, period 2018-2025*), issued by MOH in 2018, recommends reducing the average adult salt consumption to less than 7 grams/person/day. It also proposes several interventions to reduce salt intake at food service establishments, including (i) coordinating with establishment and restaurant owners to apply measures to reduce salt in their menus; (ii) providing training and instructions for chefs, cooks, and staff on techniques and measures to reduce salt in the menu; (iii) applying salt reduction measures in restaurants and bars. This involves choosing low-salt foods, reducing salt in food preparation and cooking, removing varieties and minimizing the amount of spices, fish sauce, and salt on the guest's table; (iv) providing warning messages about the health effects of eating too much salt and recommending measures to reduce salt intake to customers, such as hanging posters in	<u>*New York City (US):*</u> Voluntary salt guidelines for various restaurant and store-bought foods which evolved into the National Salt Reduction Initiative, involving nationwide partnerships among food manufacturers and restaurants.	**C**<br><br>Standards for salt content have been established in school meals. However, these standards are mainly guidance documents and are not mandatory.

		the restaurant premises, placing messages and instructions in the kitchen, placing warning and advice messages on the guest's table, and marking and indicating high-salt dishes on the customer's menu.		
**IND1.2.2 Food composition targets/standards/restrictions** have been established for the content of the nutrients of concern (trans fats, added sugars, salt, saturated fat) in **meals sold from food service outlets – SUGAR**	See definitions and scope above for **IND1.2.2**	Decision 2195/QD-BGDĐT (*Approving Guidelines for organizing school meals in combination with increasing physical activity for children and students in preschool and primary education institutions*), issued by MOET in 2022, highlights the importance of limiting sugar and salt in school menus and mandates taking measures to restrict the sale of foods that contain high amounts of sugar, salt, and fat in school canteens. The decision also sets specific standards for **sugar** intake, stating that the daily sugar intake for students should not exceed 15g per day.	*Hong Kong*: introduced the Less-salt-and-sugar Restaurant Scheme in early 2019 to encourage restaurants to provide customers with food options of reduced salt and/or sugar or tailor-made less-salt-and-sugar dishes. Some 1100 restaurants have joined the Scheme.	**C**<br><br>Standards are set for sugar consumption of students. However, there are no standards in terms of sugar content in meals served in educational institutions and other food service outlets. No clear targets and restrictions either.
**IND1.2.2 Food composition targets/standards/restrictions** have been established for the content of the nutrients of concern (trans fats, added sugars, salt, saturated fat) in **meals sold from food service outlets – TRANS FAT**	See definitions and scope above for **IND1.2.2**	Decision 2195/QD-BGDĐT (*Approving Guidelines for organizing school meals in combination with increasing physical activity for children and students in preschool and primary education institutions*), issued by MOET in 2022, highlights the importance of limiting sugar and salt in school menus and mandates taking measures to restrict the sale of foods that contain high amounts of sugar, salt, and fat in school canteens. Standards for **fat** are not set.<br><br>Apart from providing the RDI and the recommended intake of lipids (fats) (Decision 2195/QD-BGDDT; Circular 28/2016/TT-BGDDT),	*New Zealand*: The Chip Group set industry standards on chip size, serving size, cooking oil temperature, salt addition, and oil type. The standard for deep-frying oils is max 28% saturated fat, max 3% linolenic acid and max	**D**<br><br>The issue of trans-fat content in meals sold from food service outlets is not recognized as a concern in any of the policy documents.

		there are no targets, standards, or restrictions for trans and saturated fats.	1% *trans* fat. There are currently 11 registered approved oils.	

Food outlet properties

Supporting and restricting healthy/unhealthy retail

Indicators	Definitions and scope	Evidence of Vietnamese Government actions	International Best Practice Examples (Benchmarks)	Final Score and Justification
**IND2.1.1** Standards have been established to define and identify healthy/unhealthy food retailers	Definitions or guidelines with specific indicators to distinguish ‘healthy’ or healthier food outlets and “unhealthy” or “less healthy” food outlets have been set and published to the general population to encourage consumers to eat and consumer more healthy food.<br><br>Food retailers are all retail outlets offering food for consumption include markets, supermarket, grocery stores, food cooperatives, quick service restaurants, eat-in or take-away restaurants, and street/sidewalk/casual food catering outlets.	There is a market classification included in the decree 02/2003/NĐ-CP (*Development and management of marketplaces*) issued in 2003 and updated in 2009, however this classification does not consider aspects related to the healthiness of the food products sold. Decision 1371/2004/QD-BTM (the Regulation on Department Stores and Trade Centers) issued in 2004 and Decision 146/2006/QD-UB (M*echanisms to encourage investment in the construction of trade centers and supermarkets in Hanoi City*), issued in 2006 by Hanoi People Committee, proposes a classification of trade centers and supermarkets, but again this does not relate to the healthy (or unhealthy) characteristics of these outlets and the food items they sell. The main classification criteria are related to the size and scale of the outlets (e.g. area of the facilities, number of business points of sale, etc.).		**D**<br><br>No policies found. The existing policy documents do not recognize the significance and necessity of establishing standards to define and identify healthy and unhealthy food retailers
**IND2.1.2** Policies to support the development and modernization of healthy food	Healthy food retailers can be defined as retailers that primarily focus on offering and promoting a wide range of nutritious and healthy food options to consumers and	Decision 5078/QD-BCT (*Master plan for commercial development of the key economic region of the Mekong Delta until 2020, a vision to 2030*), issued by MOIT in 2013, considers the need to upgrade traditional retail channels, but also		**D**<br><br>No policies found. Overall, the limited support (and restrictions) put on some of the

retailers (i.e., retail channels that provide affordable fresh food (e.g. traditional channels such as wet markets)).	prioritize foods that align with a balanced diet. Key characteristics of a healthy food retailer typically include: (i) Nutrient-rich inventory offering a variety of healthy foods[1] that are rich in essential nutrients such as vitamins, minerals, fiber, and antioxidants. (e.g., fresh fruits and vegetables, whole grains, lean proteins, and low-fat dairy products); (ii) Availability of minimally processed food or unprocessed food options, which retain their natural nutritional integrity and are free from excessive additives, preservatives, and artificial ingredients; (iii) Limited availability of unhealthy food[2] items such as sugary snacks, high-sodium processed foods, and sugar-sweetened beverages.	strongly support the development of more modern retail channels.<br><br>Decision 9762/QD-BCT (*Project "Commercial development planning in the Southeast region to 2020, orientation to 2030"),* issued by MOIT in 2013, aims to modernize the retail sector in the Southeast region by reorganizing and upgrading traditional retailers. The decision also promotes collaboration among the different actors within the trading network to enhance the stability of the markets, ensuring that prices, availability, quality, and consumption are maintained at a satisfactory level.<br><br>Decision 1163/QD-TTg (*National strategy on domestic trade development 2021-2030, vision to 2045*), issued by the government in 2021, plans to accelerate the process of transforming the market management model, focusing on renovating and upgrading existing urban markets in towns and cities and improving service quality in the direction of ensuring food safety.		traditional retail channels does not favour the development of healthy food outlets. Policies typically foster the process of modernisation in the retail sectors; yet lack of adequate attention is paid to the availability of healthy food in these retail outlets.
**IND2.1.3** Policies to **restrict the development of unhealthy food retailers** (i.e. retail channels that provide food rich in nutrients of concern (e.g. fast-food chains)).	Unhealthy food retailer can be defined as retails that primarily specializes in offering and promoting food and beverage products that are often characterized by their low nutritional value, high levels of unhealthy ingredients, and a focus on energy-dense, heavily processed, or sugary items. Key characteristics of an unhealthy food retailer typically include: (i) Limited nutritional value of proposed products, that are low in essential nutrients such as vitamins, minerals, and fiber, while often being high in empty calories, unhealthy fats, and	Directive 08/CT-UBND (*Strengthening management of food service and street food business activities in Hanoi*), issued by Hanoi People Committee in 2016, recommends to "organize a street to concentrate food and street food services, to remove establishments encroaching on sidewalks and selling goods at unauthorized locations, inside schools, hospitals, and temporary markets, when food safety is not guaranteed." Restrictions are applied based on food safety, disregarding the healthy and unhealthy aspects of the food sold.<br><br>Decision 1163/QD-TTg (*National strategy on domestic trade development 2021-2030, vision to 2045*), issued by the government in 2021, confirms the prominent role of modern types of outlets, such as convenience stores, specialized supermarkets, and trade centers, for food retail distribution.		**D**<br><br>Experts stated that these modern retail channels are necessary in a modern society, policies only recommend diverse food items sold in these channels (including fresh fruits), but there are no specific guidelines considering the healthy or unhealthy aspects of these retail types.

[1] See definition of healthy foods

[2] See definition of unhealthy foods

	added sugars; (ii) Their inventory may consist heavily of processed and sugary foods and beverages, including fast food, sugary snacks, carbonated soft drinks, and high-calorie, low-nutrient items; (iii) Minimal fresh produce: These retailers may have limited or no availability of fresh products (such as fruits and vegetables), making it difficult for customers to access nutritious options; (iv) Unhealthy food retailers often prioritize convenience and fast-food items, encouraging the consumption of foods that are high in calories and unhealthy ingredients; (v) They may not provide transparent and easily accessible nutritional information, ingredient lists, or health-related claims on their products, making it challenging for consumers to make informed choices.	It affirms the need to "continue to improve the policy on development and management of commercial centers, supermarkets, convenience stores, vending machines, etc." ignoring the unhealthy potential of these modern channels.<br><br>Plan 122/KH-UBND (*Hanoi Plan on installation of automatic vending machines by 2025*), issued by Hanoi People's committee in 2022, promotes "the development of vending machines", which are typically used to sell ultra-processed food and carbonated drinks.<br><br>Plan 336/KH-UBND (*Food safety work plan in Hanoi city in 2023*), and Plan 49/KH-UBND (*Managing food production and trading establishments in markets in Hanoi city in 2022*), issued by Hanoi People's committee in 2022, both plan the clearance of informal markets (which are typically selling fresh and nutritious food items) and "spontaneous business points encroaching on roadways and sidewalks, that do not ensure food safety, and cause disorder in traffic safety and urban beauty".		

Food procurement and food provision

Indicators	Definitions and scope	Evidence of Vietnamese Government actions	International Best Practice Examples (Benchmarks)	Final Score and Justification
**IND2.2.1** Clear, consistent policies (including nutrition standards) in **schools and early childhood education** services for food service activities (canteens, food at events, fundraising,	Includes early childhood education and care services (for children from 0-5 years)<br><br>Schools include government and non-government primary and secondary schools (up to age 18 years) or any other vocational schools with adult students.<br><br>Includes policies and nutrition standards to provide and promote healthy food choices	Decision 712/QD-TTg (*National action program on zero hunger to 2025*), issued by the government in 2018 planned to "complete guidelines for school meals for preschool children and apply software to build nutritionally balanced menus in preschools".<br><br>Directive 584/BGDĐT-GDTC (*Strengthening nutrition work in the new situation*), issued by MOET in 2018, limits access to carbonated drinks and unhealthy food in school premises.	*<u>Ireland</u>:* The school Meals (Local Projects) Scheme, is an administrative scheme, operated directly by the Department of Employment Affairs and Social Protection (Healthy Ireland, 2017a). The Scheme provides funding to primary and post-primary schools, local groups, voluntary	**B**<br><br>Only guidelines (no mandatory regulations) for the implementation in secondary and high schools.

promotions, vending machines etc.) to provide and promote healthy food choices.	or to limit or restrict the provision or promotion of unhealthy food choices.<br><br>Includes policies that relate to school meals programs, where the program is partly or fully funded, managed or overseen by the government.	Decision 41/QĐ-TTg (*Scheme for proper nutrition assurance and physical activity increase for children and students with the aim of health improvement and prevention of cancer, cardiovascular disease, diabetes, chronic obstructive pulmonary disease and bronchial asthma for 2018 - 2025 period*), issued by the government in 2019, recommends to "develop and replicate pilot models for promoting healthy nutrition at all school levels", and to "supplement and complete regulations on nutritional standards".<br><br>Decision 1294/QD-BYT (A*ction plan to implement the national strategy on nutrition until 2025*), issued by MOH in 2022, recommends, among others, to "develop technical standards for school meals, regulations on adequate nutrition and physical activity in schools, regulations on operation of school canteens to ensure food and beverage supply beneficial to the health of students".<br><br>Decision 02/QD-TTg (*National nutrition strategy for the 2021-2030 period with a vision toward 2045*), issued by the government in 2022, recommends to "Develop guidelines for school meals to ensure reasonable nutrition according to age, and region, to ensure food diversity, and to issue regulations to limit students' access to unhealthy foods".<br><br>The Plan 266/KH-UBND (*National Strategy on Nutrition in Hanoi, period 2022-2030*), issued by Hanoi People Committee in 2022, recognizes the importance of collaboration between the education and health sectors in promoting adequate nutrition in schools. To this end, the plan emphasizes the need for an interdisciplinary approach to organizing, implementing, and monitoring activities aimed at improving students' nutrition.<br><br>Decision 2195/QD-BGDĐT (*Guidelines for organizing school meals in combination with increasing physical*	organisations, and community-based not-for-profit preschools operating their own school meals projects.<br><br>*Australia*: Six states and territories have mandatory standards for food in schools.<br>Chile: Limits on nutrient content of foods (e.g., calories, total sugar, saturated fat and sodium) in schools.<br>Brazil: School feeding programs in Brazil set nutrition standards and require schools to buy locally grown products: 70% of foods provided must be unprocessed by law.<br><br>*Jamaica:* mandatory nutrient guidelines prohibiting beverages to be sold/served in public education institutions that meet an increasing tier of g/100ml threshold (6gm/100mL - 2.5g/100ml over 4 year).	

		activity for children and students in preschool and primary education institutions), issued by MOET in 2022, outlines a comprehensive approach to ensure the provision of healthy meals to school-going children. It defines the required standards for each meal, including the level of calories, portion of protein, fat, and carbohydrate, and the acceptable levels of salt and sugar intake based on age groups. It also outlines the meal preparation process and feeding guidelines for children. The guidelines establish intake limits for sugar (15g/day) and salt (4g/day) and recommend selecting processed foods according to the criteria "low sugar, low salt, low saturated fat, supplemented with micronutrients, rich in fibers." Furthermore, it provides detailed recommendations for businesses operating canteens, such as encouraging students to choose healthy food and drinks, limiting the sale of foods containing saturated fat, salt, and sugar, and ensuring the quality and diversity of school meals.		
**IND2.2.2** Clear, consistent policies in **other public sector settings** for food service activities (canteens, food at events, fundraising, promotions, vending machines, etc.) to provide and promote healthy food choices.	Public sector settings include: - government-funded or managed services where the government is responsible for the provision of food, including public hospitals and other in-patient health services (acute and sub-acute, including mental health services), residential care homes, aged and disability care settings, custodial care facilities, prisons and home/community care services – government-owned, funded or managed services where the general public purchase foods including health services, parks, sporting and leisure facilities, community events etc. – Public sector workspaces.<br><br>Includes private businesses that are under contract by the government organisations to provide food.	Decision 02/QD-TTg (National Strategy on Nutrition for the period 2021-2030 with a vision to 2045) issued by the Prime Minister in 2022 mentions the implementation of regulations on nutrition practices in hospitals such as implementing nutrition indicators and breastfeeding in line with the Hospital Quality Criteria.<br><br>Decision 2879/QD-BYT (*Issuing "Guidelines for Diets in Hospitals*), issued by MOH in 2006, establishes catering and nutrition specifications for hospitalized patients, including children, adults, and diverse populations with varying nutrient requirements. The guidelines outline a range of nutrient criteria appropriate for each patient population, including daily requirements for energy, protein, lipids, sodium, fluids, vitamins, and minerals.<br><br>Decision 1294/QD-BYT (A*ction plan to implement the national strategy on nutrition until 2025*), issued by	*Latvia:* In 2012, the government set salt levels for all foods served in hospitals and long-term social care institutions. Levels may not exceed 1.25g of salt/100g; fish products may contain up to 1.5g salt/100g.<br><br>*Ireland*: An updated vending machine policy applies to all vending machines on government health facilities banning sugar sweetened beverages, snacks containing more than 200 calories per packet. 50% of beverages stocked will be water.<br><br>*Brazil*: Government procurement policy restricts	**C**<br><br>Policies have acknowledged the need to regulate the provision and promotion of healthy options for some public sector (hospitals, health care services, and military agencies) but have not yet policies for other public sector agencies.

	Excludes ‘public settings’ such as train stations, venues, facilities or events that are not funded or managed by the government.<br><br>Excludes school and early childhood settings (see IND2.2.1).<br><br>Includes policies and nutrition standards to provide and promote healthy food choices or to limit or restrict the provision or promotion of unhealthy food choices.<br><br>Includes the strategic placement of foods and beverages in cabinets, fridges, on shelves or near the cashier.<br><br>Includes the use of signage to highlight healthy options or endorsements (such as traffic lights or a recognized healthy symbol).<br><br>Includes modifying ingredients to make foods and drinks healthier or changing the menu to offer healthier options.<br><br>Excludes public procurement standards (see IND2.2.3).	MOH in 2022, mentions the need to develop guidelines for adequate nutrition for the army forces as well as for hospital patients.<br><br>Plan 266/KH/UBND (Implementation of the National Strategy on Nutrition in Hanoi in the period of 2022-2030) in 2022 of the Hanoi People's Committee mentioned the implementation of nutrition regulations in hospital/treatment clinics.	processed food from being sold or served within the Ministry of Health facilities and its entities.	
**IND2.2.3** Clear, consistent **procurement standards** in **public sector settings** for food service activities to provide and promote healthy food choices.	Includes standards for the public sector which encourage the procurement of healthy foods.<br><br>Includes standards for the public sector which discourage the procurement of unhealthy foods.<br><br>Includes public sector settings as defined in IND2.2.2 and IND2.2.3	Decision 2195/QD-BGDĐT (*guidelines for organizing school meals in combination with increasing physical activity for children and students in preschool and primary education institutions*) does make important recommendations such as sourcing processed foods with low levels of salt, sugar, and saturated fat, as well as including milk and dairy products in school meals, but there is no policy documents providing detailed public procurement standards for promoting healthy food choices in other public sector food service activities. Therefore, more comprehensive guidelines and standards are needed to ensure the	*UK*: The Government ‘Buying Standard for Food and Catering Services’ has nutrition standards for the public sector (hospitals, care homes and armed forces).<br><br>*Brazil*: A school food procurement law approved in 2001, limits the number of processed foods purchased by schools to 30%, and bans the	**D**<br><br>Food in public sector settings is not on the list of public procurement activities of the government, so there are no policies about public

		provision of nutritious meals in educational institutions.	procurement of drinks with low nutritional value, such as sugary drinks. The law requires schools to buy locally grown or manufactured products, supporting small farmers and stimulating the local economy.	procurement for food. Policies related to procurement for food will be normally issued by relevant ministries, such as Ministry of Education and Training and Ministry of Health.
**IND2.2.4 Training system** to help **schools and other public sector organisations** and their caterers meet the healthy food service policies and guidelines.	Includes support for early childhood education services as defined in IND2.2.1<br><br>Public sector organisations include settings defined in IND2.2.2.<br><br>Support and training systems include guidelines, toolkits, templates (e.g., policy/guidelines or contracts), recipes and menu planning tools, expert advice, menu and product assessments, online training modules, cook/caterer or other food service staff information and training workshops or courses	Decision 1294/QD-BYT (A*ction plan to implement the national strategy on nutrition until 2025*), issued by MOH in 2022, includes recommendations for strengthening the public officer's expertise in nutrition and agriculture. Specifically, the decision calls for the development of a nutrition training program aimed at school staff involved in food service activities.<br><br>Decision 41/QĐ-TTg (*Scheme for proper nutrition assurance and physical activity increase for children and students for 2018 - 2025 period*), issued by the government in 2019, recommends that all employees who work at collective kitchens in educational institutions and vocational training institutions should undergo proper nutrition training.<br><br>Decision 1768/QD-BYT (*Guidelines for the implementation of the content "Improving nutrition" under the National Target Program for Sustainable Poverty Reduction in the 2021-2025 period*), issued by MOH in 2022, is a comprehensive policy document that goes beyond other policy documents by recommending training a wider range of public officers, beyond educational institutions, on proper nutrition for women, children, and adolescents.<br><br>Decisions 2195/QD-BGDĐT (*Guidelines for organizing school meals in combination with increasing physical*	*Australia*: The Healthy Eating Advisory Service supports childcare centers, schools, workplaces, health services, food outlets and sports centers to provide healthy foods and drinks.<br>*Japan*: The Cabinet Office has introduced laws to promote 'shokuiku' (diet, growth and education). Concepts include promotion of 'shokuiku' at home, schools, provision of dietary guidance by dieticians in food service facilities, diet and nutrition teachers and school lunch practice standards.	**C**<br><br>Policies acknowledge the need for training public officers, and the need to develop nutrition training and school health programs. However, there are no regulations for granting certificates to trainees after nutrition training, only food safety certificates are issued.

		activity for children and students in preschool and primary education institutions), Decision 4280/QD-BGDĐT (*Guidelines for organizing school meals combined with increased physical activity for junior high school students*), issued by MOET in 2022, make recommendations for the development of guidelines for nutrition training programs for school staff.<br><br>Decision 1660_QD_TTg (*School Health Program for the period of 2021-2025*), issued by the government in 2021, includes a recommendation to provide nutrition training to medical staff.<br><br>Decision 2033/QD-BYT (*National plan for communication and advocacy to reduce salt in the diet, period 2018-2025*), issued by MOH in 2018, recommends the provision of instructional documents and training for chefs, cooks, and restaurant staff on techniques and measures to reduce salt in menus.		
**IND2.2.5** Policies that actively encourage and support **private companies** to provide and promote healthy foods and meals in their workplaces.	For the purpose of this indicator, 'private companies' includes for profit companies and extends to non-government organisations (NGOs) including not-for-profit/charitable organisations, community-controlled organisations, etc.<br><br>Includes healthy catering policies, fundraising, events, -includes support and training systems such as guidelines, toolkits, templates (e.g. policy/guidelines or contracts), recipes and menu planning tools, expert advice, menu and product assessments, online training modules, cook/caterer and other food service staff information and training workshops or courses (where relevant to the provision of food in a workplace)	**Clear orientation but no specific policies to support private companies to provide and promote healthy food choices in workplaces.**<br><br>Resolution 07c/NQ-BCH dated February 25, 2016, of the Vietnam General Confederation of Labor on "Quality of shift meals for employees" set the target to ensure workers' shift meals at the minimum of 15,000 VND and encourage businesses to increase the value of shift meals higher to ensure providing nutritional meals for their employees.<br><br>Decision 02/QD-TTg (National strategy on nutrition for the period 2021-2030 and vision to 2045) of the Prime Minister in 2022 assigned the Ministry of Labor, War Invalids and Social Affairs to coordinate with other relevant agencies to promote propaganda and direct the implementation of sufficient nutrition	*Australia*: Victoria has 'Healthy choices: healthy eating policy and catering guide for workplaces' supported by a Healthy Eating Advisory Service.<br><br>*Singapore*: The National Workplace Health Promotion Program provides tools and resources for healthy workplace nutrition.	**D**<br><br>A few policies just encourage dissemination activities about sufficient nutrition. There are no specific regulations to encourage and support private companies to provide and promote healthy food and meals in their workplaces.

	Excludes the provision or promotion of food to people not employed by that organisation (e.g. visitors or customers).<br><br>Excludes support for organisations to provide staff education on healthy foods	for workers, especially female workers, pregnant female workers, nursing mother workers, workers in industrial parks, workers in heavy, hazardous, and dangerous occupations and jobs, especially hazardous and dangerous occupations and jobs.<br><br>Decision 1294/QD-BYT (*Action plan to implement the national strategy on nutrition until 2025*), issued by MOH in 2022, recommends "to develop recommendations on workers' meals suitable to the industry, and to disseminate and apply them in production, business, and industrial zones", as well as "to develop and implement models to improve nutritional status, collective meals of employees, and providing nursing rooms in the workplace".		

Food Marketing

Food promotion

Indicators	Definitions and scope	Evidence of Vietnamese Government actions	International Best Practice Examples (Benchmarks)	Final Score and Justification
**IND3.1.1** Policies to restrict exposure and power of promotion of unhealthy foods through **broadcast media (TV, radio) and online/social media.**	Includes mandatory policy (i.e. legislation or regulations) or voluntary standards, codes, guidelines set by government or by industry where the government plays a role in development, monitoring, enforcement or resolving complaints (i.e. co-regulation).<br><br>Includes free-to-air and subscription television and radio and online media (e.g.	Law 36/2005/QH11 (Commercial) of the National Assembly issued in 2005 regulates the prohibition of advertising, promotion or use of alcohol and beer for promotion to people under 18 years of age. It is prohibited to advertise, promote, or use tobacco or alcohol with an alcohol content of 30 degrees or more for promotion in any form. Promotions for the consumption of poor-quality goods that are harmful to the environment, human health and other public interests are prohibited. At the same time, it is forbidden to advertise falsely about any of the following contents: quantity, quality, price, utility, design, origin of goods, type, packaging, service method, warranty period of the product. goods, services.<br><br>Law 16/2012/QH13 (Advertising Law) and Consolidated Document 47/VBHN-VPQH (Advertising Law) of the National Assembly in 2018 provide minimum regulations to limit and transparently inform	*UK*: Restrictions banning advertising of foods high in fat, salt or sugar on TV before 9pm and paid-for adverts online will come into force in January 2024. This restriction is a key part of the government's commitment to reduce obesity.	**D**<br><br>Minimum regulations to regulate, control, and limit the promotion and transparently inform consumers whether products are healthy or unhealthy have been mentioned. However, restricting the promotion of unhealthy foods in

	social media, branded education websites, online games, competitions, and apps)<br><br>Effective means that the policies are likely to reduce overall exposure of general population to unhealthy food advertising over the day.	consumers about advertising activities. Whether the product is healthy or not. In addition, in legal and sub-legal documents, there are regulations aimed at transparency and providing more information to consumers that are considered the minimum level to limit exposure and the impact of advertising.<br><br>Decision 376/QD-TTg (*Approval of the national strategy for preventing and controlling cancers, cardiovascular disease, diabetes, chronic obstructive pulmonary disease, bronchial asthma and other non-infectious diseases during the 2015-2025 period*), issued by the government in 2015, and Decision 1092/QD-TTg (*Approval of Vietnam Health Plan*), issued by the government in 2018, both recommend to develop appropriate mechanisms to control and limit the advertising of soft drinks, processed foods and a number of other products with the risk of causing non-communicable diseases.<br><br>The Directive 46/CT-TTg (*Enhancement of Nutrition in new circumstances*), issued by the government in 2017, mentions the need to limit advertising of unhealthy nutritional products according to regulations.<br><br>Decision 5924/QD-BYT (*Implementation plan of the Vietnam health program in the 2021-2025 period*), issued by MOH in 2021, plans to develop and submit to competent authorities for promulgation appropriate mechanisms and legal regulations on management and control of advertising (as well as business and tax increase) to limit the use of sugary drinks and ready-made foods, food additives and a number of other products with health risks, especially products for children.<br><br>Decision 1294/QD-BYT (A*ction plan to implement the national strategy on nutrition until 2025*), issued by MOH in 2022, recommends “to add and complete policies and regulations to control the advertising of unhealthy foods and products, especially products for children and pregnant women.” And “to develop regulations on limiting advertising, promotion and sponsorship of brands for unhealthy foods, especially for children.”		mass media and online/social networks has not been specifically mentioned and has clear regulations

**IND3.1.2** Policies to **restrict exposure and power of promotion** of unhealthy foods to **children** (including adolescents) through **broadcast media (TV, radio) and online/social media**	Includes mandatory policy (i.e. legislation or regulations) or voluntary standards, codes, guidelines set by government or by industry where the government plays a role in development, monitoring, enforcement or resolving complaints (i.e. co-regulation).<br><br>Includes free-to-air and subscription television and radio and online media (e.g. social media, branded education websites, online games, competitions, and apps)<br><br>Effective means that the policies are likely to reduce overall exposure of children, including adolescents to unhealthy food advertising over the day.	Law 16/2012/QH13 (Advertising Law) and Consolidated Document 47/VBHN-VPQH (Advertising Law) of the National Assembly in 2018 provide minimum regulations to limit children's exposure to food advertising. not good for health. Specifically, the Law prohibits advertisements that cause children to have thoughts, words, and actions that are contrary to morality and good customs; cause adverse effects on the health, safety or normal development of children.<br><br>The Decree 38/2021/NĐ-CP (*Regulations on sanctioning administrative violations in the field of culture and advertising*), issued by the Government in 2021, sets out a fine ranging from VND 10,000,000 to 20,000,000 for publishing advertisements that may have a negative impact on children's health. However, the decree does not specify what constitutes a "negative impact on children's health", nor does it mention anything about unhealthy foods.<br><br>Decision 1294/QD-BYT (*Action plan to implement the national strategy on nutrition until 2025*), issued by MOH in 2022, emphasizes the need for strengthening educational communication and disseminating nutrition knowledge through social media platforms such as TikTok, Instagram, and Zalo, with content that covers a range of topics, including nurturing young children, nutrition for school-age children, nutrition for the prevention of non-communicable diseases, nutrition in emergencies and epidemics, and how to read and understand nutrition labels.	<u>*Quebec*</u>: All advertising to children under 13 years old banned since 1980.<br><br><u>*Ireland*</u>: Advertising of unhealthy foods during children's TV and radio programmes (when over 50% of the audience is under 18) prohibited. Advertising to children under 13 must not feature celebrities.<br><br><u>*South Korea*</u>: Since 2010 TV food ads before, during and after programmes between 5-7pm and during other children's programmes prohibited. Children are defined as 4-18 yrs. The regulation of TV ads also applies to the Internet.<br><br><u>*Chile*</u>: the Law of Nutritional Composition of Food and Advertising restricts advertising directed to children under 14 years (for foods exceeding limits for calories, sugar, saturated fat and/or sodium in food and beverages). This includes promotional strategies and premium offers.	**C**<br><br>There are minimal regulations regarding restrictions on advertising of unhealthy foods to children. There are no specific regulations to limit advertising of unhealthy foods to children in mass media and online/social networks.
**IND3.1.3** Policies to ensure that **unhealthy foods are not commercially promoted to children** (including adolescents) **in settings where**	Children's settings include areas in and around schools, preschools/ kindergartens, day-care centers, children's health services (including primary care, maternal and child health or tertiary settings), sport, recreation	The directive 46/CT-TTg (*Enhancement of Nutrition in new circumstances*) issued by the government in 2017, the directive 584/BGDĐT-GDTC (*Strengthening nutrition work in the new situation*), issued by MOET in 2018, the decision 02/QD-TTg (*Approving the national nutrition strategy for the 2021-2030 period with a vision toward 2045*), issued by the government in 2022, and the decision 718/QD-BYT (*Approving the national plan of action for nutrition to 2020*), issued by MOH in 2018, recommend not	<u>*Chile*</u>: The Law of Nutritional Composition of Food and Advertising restricts advertising directed to children under 14 years (for foods exceeding limits for calories, sugar, saturated fat, sodium in food and beverages). This includes the school grounds.	**C**<br><br>Restrictions on advertising of unhealthy foods in and around schools have been recognized in the

**children gather** (e.g. preschools, schools, sport, and cultural events).	and play areas/venues/facilities and cultural/community events where children are commonly present.<br><br>Includes restrictions on marketing in government-owned or managed facilities/venues (including within the service contracts where management is outsourced)<br><br>Includes restrictions on unhealthy food sponsorship in sport (e.g. junior sport, sporting events, venues).<br><br>Effective means that the policies are likely to reduce overall exposure of children, including adolescents to unhealthy food advertising over the day.	advertising and sell alcoholic beverages, sugary drinks and other unhealthy foods in and around schools. This is also mentioned in local level plans, like in the plan 266/KH-UBND (*National Strategy on Nutrition in Hanoi, period 2022-2030*), issued by Hanoi People Committee in 2022.<br><br>Decision 1294/QD-BYT (*Action plan to implement the national strategy on nutrition until 2025*), issued by MOH in 2022, recommends the addition and completion of policies and regulations aimed at controlling the advertising of unhealthy foods in schools, in order to promote a healthier food environment.	*Spain*: the Law on Nutrition and Food Safety states that kindergartens and schools should be free from all advertising.<br><br>*Hungary*: All advertising directed at children under 18 years in schools and institutes is prohibited.	documents. However, the new regulations only stop at incentives without specific policies and sanctions
**IND3.1.4** Policies to ensure that **unhealthy foods are not commercially promoted to children** (including adolescents) **on food packages**.	Includes product design and packaging (e.g. use of celebrities or cartoons, competitions and giveaways)<br><br>Effective means that the policies are likely to reduce overall exposure of children, including adolescents to unhealthy food advertising over the day.	No relevant content found in the existing policy documents.	*Chile*: Law Number 20.606 (Nutritional Composition of Food and Advertising) specifically seek to regulate companies with brands that target children through misleading advertising and the use of cartoon mascots on commercial packaging.	**D**<br><br>The need to restrict promotion of unhealthy food to children on food packages is not acknowledged in existing policy documents.

**IND3.1.5** Restrict marketing of **breast milk substitutes**	Prohibition of all advertising and other forms of promotion of breastmilk substitutes including infant formular (marketed for use from birth), follow-on formular (marketed for use from 6 months of ages) and milks for older babies (up to 36 months) on any types of media and communication channels.<br><br>Effective means that the policies are likely to protect and promote breastfeeding and its nutritional and health benefits; as well as reduce the exposure of parents/ guardians to misleading, inaccurate or biased marketing claims about breast milk substitute products and consequently threatens the health of the population.	Decision 307-TTg (*Trade and use of substitutes for breast milk in support of breast feeding*) issued in 1994 by the government (and still active), sets fines (ranging from VND 50,000 to 500,000) for advertising breast milk substitutes, while the recently updated Decree 38/2021/NĐ-CP (Regulations on sanctioning administrative violations in the field of culture and advertising) increases the fines significantly (ranging from VND 50,000,000 to 70,000,000).<br><br>The Law 16/2012/QH13 (*Law on Advertising*) issued by the National Assembly in 2012, bans advertising of milk substitutes for breast milk for children under 24 months old, nutritional supplements for children under 6 months old; feeding bottles and pacifiers for children according to Article 7, while no restrictions are in place for specific food items that might have adverse impact on children's health.<br><br>The Decree 181/2013/ND-CP (*Details implementation of some articles of the advertising law*), issued by the government in 2013, provides further details on the conditions for advertising content for milk and nutritional products for children under 36 months old in which advertising contents must be registered and approved by the authorized agency in advance.<br><br>The Decree 100/2014/ND-CP (*trading and use of nutritious products for infants*), issued in 2014, aims to protect the health and well-being of infants and young children by prohibiting the advertising of breast milk substitutes for children under 24 months and complementary foods for children under 6 months old. These decrees specify various ways in which advertising is prohibited, including direct or indirect contact with mothers, pregnant women, or their families for the purpose of promoting these products, displaying names and logos of breast milk substitutes and complementary foods in retail stores and medical facilities, and offering promotional measures like samples, coupons, prizes, discounts, or scholarships to propagate and encourage the use of these products. The decrees also prohibit information, education, and communication related to nurturing young children that promote the use of breast milk substitutes. The decrees outline specific activities that physicians, medical staff, and health facilities should not engage in regarding the promotion of breast milk	<u>UK</u>: The Formula and Follow-on Formula Regulations (2007) prohibit advertising and promotion of infant formula only (marketed for use from birth). Follow-on formula (marketed for use from 6 months of age) and milks for older babies can be advertised and promoted.	**A**<br><br>Existence of multiple policies to restrict the marketing of breast milk substitutes. These policies prohibit aggressive marketing practices, misleading advertising, and promotional activities that undermine breastfeeding. The policies also include regulations on the labeling, packaging, and promotion of breast milk substitutes to ensure that they are used appropriately and as a last resort. However, Vietnam's regulations are not consistent with WHO and Codex recommendations. According to WHO and Codex recommendations, advertising and marketing of products for children under 36 months old is prohibited, while Vietnamese law only prohibits advertising

		substitutes. In addition, the decrees provide guidance on what information, education, and communication materials can and cannot contain.		and marketing for children under 24 months old.

Food labelling

**Indicators**	**Definitions and scope**	**Evidence of Vietnamese Government actions**	**International Best Practice Examples (Benchmarks)**	**Final Score and Justification**
**IND3.2.1** Policies to ensure that **ingredient lists and nutrient declarations** in line with NIN/WHO recommendations are present on the labels of all packaged foods.	Includes packaged food manufactured in country our overseas and imported to Vietnam.<br><br>Nutrient declaration means a standardized statement or listing of the nutrient content of a food.<br><br>Excludes health and nutrition claims (see IND3.2.2).<br><br>Includes trans fats and added sugar which are not part of the standard seven elements generally part of mandatory nutrient declarations (energy, total fat, saturated fat, trans fat, carbohydrates, sugar, protein, sodium).	Resolution 20-NQ/TW (strengthening the protection, care and improvement of people's health in the new situation) issued by the Central Executive Committee in 2017 sets out a common goal for the Vietnamese people by 2030. Men have improved physical and mental health, quality of life, and longevity, with the new "Recommending and disseminating appropriate nutritional regimes and diets for each target group, source of ingredients, and taste." of Vietnamese people. Packaged products and foods must have full information about ingredients, energy, and health warnings on the packaging."<br><br>Decree 119/2017/ND-CP regulates penalties for administrative violations in the field of standards, measurement and quality of products and goods and Decree 126/2021/ND-CP amends and supplements a number of articles of decrees regulating penalties for administrative violations in the field of industrial property; standards, measurement and quality of products and goods; science and technology activities, technology transfer; Atomic energy has regulations on administrative penalties for mislabelling products<br><br>Decree 100/2014/ND-CP (*trading and use of nutritious products for infants*) issued by the Government in 2006, sets out list of contents should be declared on the labels of food and nutritional products for young children in which all contents must be written in Vietnamese,	*Wide range of countries in Europe, NZ, Australia etc.*: It is required by law to provide a comprehensive nutrient list on packaged food products regardless of whether there is a health or nutrition claim.<br><br>*New Zealand*: NZ participates in Codex and has a Food Standards Code (2002) outlining the labelling regulation.	**B**<br><br>The need for labeling is acknowledged. Ingredient lists and nutrition declarations are required by law. However, these regulations are still not in sync with WHO recommendations, especially regulations related to Trans fats and added sugar content.

		information about "the origin, production and expiry date, ingredients, certificate number for publication of quality standards, food hygiene and safety and nutritional information of the products" must be clearly presented on the product's labels.<br><br>Decree 43/2017/ND-CP (Goods labelling) issued by the Government in 2017, describes the compulsory content that must be displayed on the label by the characteristics of the goods. Decree 111/2021/ND-CP was later issued in 2021 amending *some articles of Government's Decree No. 43/2017/ND-CP*, specifies the required information to be displayed on food packaging, including the quantity, production date, expiry date, ingredients or quantity of ingredients, and nutritional information such as the nutritional value (if available). Food products subjected to the policies are food items, food for health, food that has been irradiated, genetically modified food; beverage (except alcohol), alcohol, micronutrients. Food items exempted from the labelling requirements are "Commodities are fresh, raw, processed foods without packaging and sold directly to consumers".<br><br>Decision 155/QD-TTg 2022 Approving the national plan for prevention and control of non-communicable diseases and mental health disorders for the period 2022 - 2025 "regulations on mandatory nutritional labelling on food products for publication" information on the content of salt, sugar, fat and other related ingredients."<br><br>Decision 5924/QD-BYT (I*mplementation plan of the Vietnam health program in the 2021-2025 period*), issued by MOH in 2021, and Decision 1294/QD-BYT (A*ction plan to implement the national strategy on nutrition until 2025*), issued by MOH in 2022, aim to develop a circular on nutrition labelling on the front of the packaging of pre-packaged products, particularly breast milk substitutes and complementary foods. The circular is expected to include technical criteria on nutritional recommendations for Vietnamese people, the maximum tolerance threshold, and nutritional reference values. However, the document		

		does not specify whether the criteria should be aligned with WHO recommendations.<br><br>Circular 43/2014/TT-BYT (*Regulating the management of functional foods*), issued by the Government in 2014, describes the compulsory contents that must be displayed on the label (incl. amount, production and expired dates; list of ingredients, nutrition values, use instructions) by the characteristics of the goods. The policy covers common food, alcoholic and non-alcoholic drinks and extends to specialized food supplements.<br><br>Decision 1092/QD-TTg (*Approval of Vietnam Health Plan*), issued by the government in 2018, mandates the labelling of processed foods to include information on energy, salt, sugar, saturated fat, trans fat, and micronutrients. The decision also requires the inclusion of health warnings for consumers and emphasizes the importance of strict implementation of these labelling requirements.<br><br>Decision 2033/QD-BYT (*National plan for communication and advocacy to reduce salt in the diet for prevention and control of hypertension, stroke and other non-communicable diseases, period 2018-2025*), issued by MOH in 2018, requires that food labelling information must include: (i) the amount of added salt in the food, (ii) a warning label on foods with high salt content, (iii) a warning about the potential health risks associated with excessive salt intake, and (iv) a recommendation for the maximum daily salt intake.<br><br>Decision 376/QD-TTg (*Approval of the national strategy for preventing and controlling cancers, cardiovascular disease, diabetes, chronic obstructive pulmonary disease, bronchial asthma and other non-infectious diseases during the 2015-2025 period*), issued by MOH in 2015, recommends the strict regulation of food labelling for processed foods. Specifically, it mandates the declaration of energy, salt, sugar, saturated fat, trans fat, and micronutrients in food, as well as the inclusion of health warnings on food labels for consumers.		

		Joint circular 45/2015/TTLT-BNNPTNT-BKHCN (*Labeling of prepacked genetically modified foods*), issued by MARD and MOST in 2015, outlines mandatory requirements for the labeling of pre-packaged genetically modified foods.		
**IND3.2.2**<br>Evidence-based **regulations for reviewing and approving claims** on foods, so that consumers are protected against unsubstantiated and misleading nutrition and health claims.	Nutrition claims include references to the nutritional content of food (e.g., low in fat)<br><br>Health claims are claims that state, suggest or imply that a relationship exists between a food category, a food or one of its constituents and health. These include function claims, such as 'calcium strengthens bones' or 'good for your heart' and disease risk reduction claims, such as 'A healthy diet rich in a variety of vegetables and fruit may help reduce the risk of some types of cancer'.<br><br>Includes the use of a nutrient profiling system to classify food products into permitted/ not permitted to carry health claims and/or nutrition claims.<br><br>'Evidence-based' refers to regulations that are based on an extensive review of up-to-date research and expert input or a validated nutrient profiling model to inform decision-making about nutrition or health claims.	Law 16/2012/QH13 (Law on Advertising) and Document Consolidated Document 47/VBHN-VPQH of 2018 consolidate the Law on Advertising promulgated by the Office of the National Assembly to prohibit advertising that is incorrect or causes confusion regarding business viability. business, the ability to provide products, goods and services of organizations and individuals trading in products, goods and services, about quantity, quality, price, utility, design, packaging, brand, origin, type, service method, warranty period of registered or registered products, goods and services. to be announced<br><br>Decree 181/2013/ND-CP details the implementation of a number of articles of the Law on Advertising, with specific regulations on advertising content that must be consistent with the Certificate of Compliance Declaration or Certificate of Compliance. food safety regulations. At the same time, request confirmation of advertising content for special products, goods and services.<br><br>Decree 15/2018/ND-CP detailing the implementation of a number of articles of the Law on Food Safety, stipulating mandatory labelling content on food labels.<br><br>Decree 43/2017/ND-CP (Goods Labels) of the Government in 2017 stipulates products with ingredients or substances in the complex composition of goods of special types using preservatives that are not prescribed dosage and listed as irritating, toxic to humans, animals and the environment or products and components of irradiated goods, applying techniques to transform gene must clearly state the name of the preservative and the warning information attached to these ingredients.	*New Zealand/Australia*: New Standard 1.2.7 to regulate nutrition content claims and health claims was approved in January 2013 in NZ and Australia, including 200 pre-approved food-health relationships and 13 pre-approved food-health relationships for high level health claims; a nutrient profiling system used for health claims but not for nutrient content claims.<br><br>*Indonesia*: Any nutrition or health claim may only be used on processed foods or beverages if they do not exceed a certain level of fat, saturated fat, and sodium per serving.<br><br>*US:* Nutrient-content claims are generally limited to nutrients authorized by the Food and Drug Administration (FDA). Food packages displaying a nutrient content claim must include a disclosure statement if a serving of the food exceeds a certain level of fat, saturated fat, cholesterol, or sodium. Health claims are not permitted if those levels of nutrients of concern are exceeded.	**C**<br><br>There are regulations on a management system based on evidence review and approval of claims on food labels to protect consumers from misleading or unfounded nutritional and health claims. However, the existing regulations tend to focus on food safety rather than on the overall food quality and nutritional content.

		Circular 43/2014/TT-BYT (*Regulating the management of functional foods*), issued by MOH in 2014, introduces basic guidelines for food-related health claims on the packaging, but it only applies to functional foods. Other processed and ultra-processed food items are not subject to any specific regulations in that regard.<br><br>Decree 111/2021/ND-CP Amending and supplementing a number of articles Decree No. 43/2017/ND-CP dated April 14, 2017, of the Government on goods labels detailing a number of mandatory contents shown on goods labels, in which groups of food and food products are required to write warning information. However, warning information to ensure safety for health, property and the environment during transportation, storage, preservation, and use, excluding warnings about nutrition and health.		
**IND3.2.3** Policies to ensure that evidence-informed **front-of-pack supplementary nutrition information system**, which allow consumers to assess a product's healthiness, is applied to all packaged foods (e.g. Nutri-Score, Traffic light label).	Nutrition information systems include traffic light labelling (overall or for specific nutrients); warning labels; Nutriscore; star or points rating; percent daily intake.<br><br>Evidence-informed' refers to systems that utilize robust criteria (based on an extensive review of up-to-date research and expert input) or a validated nutrient profiling model to inform decision making about the product's healthiness	No relevant content found in the existing policy documents.	*New Zealand/Australia*: Voluntary Health Star Rating system.<br><br>*France/Belgium/Germany/Spain* five-color NutriScore front-of-pack labelling system with colors instead of stars.<br><br>*Chile*: Mandatory warning labels on foods. Foods and beverages exceeding certain limits for calories, sugar, saturated fat and sodium need to have a front-of-package stop sign warning label.	**D**<br><br>No relevant content found in the existing policy documents to acknowledge the need for, or establish, an evidence-informed front-of-pack supplementary nutrition information system.

**IND3.2.4** Policies to set a simple and clearly visible system of **labelling the menu boards** of all quick service restaurants (i.e. fast-food chains), which allows consumers to interpret the nutrient quality and energy content of foods and meals on sale.	Quick service restaurants: in Vietnam's context this definition includes fast food chains, local own snack bars (quán đồ ăn vặt), coffee, bakery. It may also include supermarkets where ready-to-eat foods are sold.<br><br>Definition Euromonitor: Fast food outlets offer limited menus that are prepared quickly. Customers order, pay and pick up their order from a counter. Outlets tend to specialize in one or two main entrees such as hamburgers, pizza, ice cream, or chicken, but they usually also provide salads, drinks, dessert etc. Food preparation is generally simple and involves one or two steps, allowing for kitchen staff generally consisting of younger, unskilled workers. Other key characteristics include: • A standardized and restricted menu; • Food for immediate consumption; • Tight individual portion control on all ingredients and on the finished product; • Individual packaging of each item; • Counter service; • A seating area, or close access to a shared seating area, such as in a shopping center food court • For chained fast food, chained and franchised operations which operate	No relevant content found in the existing policy documents.	*Australia*: Mandatory kJ menu board labelling for chain fast food outlets in New South Wales, South Australia and ACT.<br><br>*South Korea*: Since 2010, all chain restaurants with 100 or more establishments are required to display energy, total sugars, protein, saturated fat and sodium content on menus.<br><br>*Canada:* Ontario requires food service outlets part of a chain of 20 or more to display calories on labels, menus and display tags.<br><br>*Saudi Arabia:* Requires mandatory measures calorie labels on all menus in facilities that serve food.	**D**<br><br>No relevant content found in the existing policy documents to acknowledge the need for, or set, a simple and clearly visible system of labelling the menu boards of all quick service restaurants.

	under a uniform fascia and corporate identity. • Take out is generally present, as is drive-through in some markets.<br><br>Labelling systems: Includes any point-of-sale (POS) nutrition information such as total kilojoules; percent daily intake; traffic light labelling; star rating, or specific amounts of nutrients of concern, salt warning labels.<br><br>Includes endorsement schemes (e.g., accredited healthy choice symbol) on approved menu items			

Food Desirability

Indicators	Definitions and scope	Evidence of Vietnamese Government actions	International Best Practice Examples (Benchmarks)	Final Score and Justification
**IND4.1.1** Existence of clear and **evidenced-informed food based dietary guidelines** supporting nutrition information and communication.	Food-based dietary guidelines should be for both genders and key age groups including infants and pregnant women.<br><br>Evidence-informed includes extensive review of up-to-date research	Decision 641/QD-TTg (*General project for development of Vietnam's health and stature for 2011 – 2030),* issued by the Government in 2011, aims to strengthen care for reproductive health, maternal and newborn health, sharply reducing the rate of malnutrition to improve basic indicators of 5-year-old children and ensure physical fitness assessment criteria, physical stature of Vietnamese youth in adulthood. It proposes to continue the nutritional care program for pregnant mothers and children under 5 years old; develop standard daily nutritional menu, undertake interventions on nutritional care programs for children in kindergarten, primary, junior high and high school students, and develop and implement school milk program for kindergarten and primary school students.	*Brazil*: This country launched landmark new dietary guidelines in 2014 from a cultural, ethical and environmental aspect. They focus on reducing the consumption of ultra-processed food products. They also provide advice on planning, shopping and sharing meals, as well as warning people to be wary of food marketing and advertising.	**A**<br><br>Nutrition related policies are well established for the target groups such as infants, young children, and pregnant mothers. There are also nutritional regulations on meals

	and mechanisms to seek expert input.<br><br>Evidence includes ways the Food-based dietary guidelines have been used to develop/implement policies to improve diets	Circular 43/2014/TT-BYT (*Regulations on management of functional food*) issued by MOH in 2014, provides information on the recommended nutritional demand for Vietnamese by different groups of micronutrients, vitamin and minerals for different age groups including, infant less than 12 months, children under 9, male and female adolescents, male and female adults, pregnant women, and breastfeeding mothers.<br><br>Decision 712/QD-TTg (*National Action Program "Zero Hunger" in Vietnam by 2025*), issued by the Government in 2018, elaborates on the need to develop guidelines to help enhance availability and diversity of food at the household level, especially for poor households, and households with children under 2 years old to ensure food security and nutrition security, improving the nutritional status of ethnic minorities, disadvantaged areas and areas with high malnutrition rates. Decision 02/QD-TTg (*National nutritional strategy for 2021-2030 and vision to 2045*) issued by the Government in 2022, further recognizes the necessity for models of nutrition agriculture and guidelines to ensure food security and quality of meals at households.<br><br>Resolution 34/NQ-CP (*Ensuring National food security toward 2030*), issued by the Government in 2021, states the need to improve the effectiveness of communication and education on people's health by disseminating information about appropriate diets for each target group.<br><br>Decision 5924/QD-BYT (I*mplementation plan of the Vietnam health program in the 2021-2025 period*), issued by MOH in 2021, emphasizes the need to develop a recommended menu and nutritional diets guidelines containing local and indigenous ingredients and to provide regulations and guidelines on healthy diets for different groups including, the community, the elderly, women and children and people in the workforce.<br><br>Decision 1294/QD-BYT (A*ction plan to implement the national strategy on nutrition until 2025*), issued by MOH in 2022, recommends developing a range of nutritional guidelines to prevent overweight, obesity and non-communicable diseases. Guidelines on diets and nutritional practices for different target groups including	*The Netherlands*: The Dutch Health Council published the 'Guidelines Good Food' 2015. These guidelines advise to eat more plant-based and less animal based food and include advice on the intake of different food products.	for crew members, laborers, and ensuring food hygiene and food safety for collective kitchens.<br><br>Nutrition-related policies should also pay more attention to remote areas.

		working population, staff and patients at hospitals, army forces, etc. with different needs are also considered to be developed in different communication formats targeting different groups across media channels.<br><br>Decision 2195/QD-BGDDT (*Guidelines for organizing school meals in combination with increasing physical activity for children and students in preschool and primary schools*), issued by the Government in 2022, introduces guidelines for organizing schools meals for kindergartens and primary schools, providing detail information on nutrition requirements for different age groups, principles for meal planning, developing menu, food selections, preparation and serving, conditions for food processing areas and guideline for school's caterers, etc.<br><br>Decision 02/QD-TTg (*National nutritional strategy for 2021-2030 and vision to 2045*), issued by the Government in 2022, recognizes the necessity for models of nutrition agriculture and guidelines to ensure food security and quality of meals at households. The Zero Hunger Program issued in Decision 712/QD-TTg in 2018 further elaborates on the need to develop guidelines to help enhance availability and diversity of food at the household level, especially poor households, and households with children under 2 years old to ensure food security and nutrition security, improving the nutritional status of ethnic minorities, disadvantaged areas and areas with high malnutrition rates.<br><br>Decision 2879/QD-BYT 2006 of the Ministry of Health on hospital diet guidelines provides specific guidelines on the hospital diet, in which the levels of sugar, cholesterol and salts were specified in the structure of the diet.<br><br>In addition, the health sector also builds nutrition pyramids for different aged groups such as nutrition pyramids for the elderly, nutrition pyramids for adults, nutrition pyramids for children, etc. In which, groups containing high levels of sugar, salt, and fat are commonly placed at the top of the nutritional pyramid - these are among the nutrition of concern that should have limited consumption in the diet.		

		Circular 40/2017/TT-BYT, issued by the Ministry of Health in 2017, regulates hygiene and safety standards for food, drinking water and meal quantities for seafarers working on ships.<br><br>Decree 09/2016/ND-CP, issued by the Prime Ministers in 2016, provides regulations on fortification of micronutrients in food.		
**IND4.1.2 Policies supporting** the establishment of national communication campaign and **nutrition awareness activities**	Includes documented plans with specific actions and interventions (i.e. policies, programs, partnerships)<br><br>Plans should be current (i.e., maintain endorsement by the current government)<br><br>Includes priority policy and program strategies, media, and social media marketing for public awareness.	The documents below prioritize nutrition for children, with communication efforts focusing on the benefits of breastfeeding, proper nutritional care for children under 5 years of age, the importance of nutrition for comprehensive development of children's stature and intellect, and the role of proper nutrition in preventing malnutrition, stunting, overweight and obesity, and nutrition-related chronic non-communicable diseases:<br><br>Decision 641/QD-TTg (*General Project for Development of Vietnam's Health and Fitness for 2011 – 2030*), issued by the government in 2011.<br><br>Decision 226/QD-TTg (*National Strategy on Nutrition for 2011 - 2020 and Vision to 2030*), issued by the government in 2012.<br><br>Decision 100/2014/ND-CP (*Business and Use of Nutritional Products for Children, Bottles and Artificial Nuts*), issued by the government in 2014.<br><br>Decision 712/QD-TTg (*National Action Program "Zero Hunger" in Vietnam by 2025*), issued by the government in 2018.<br><br>Decision 41/QD-TTg (*Project "Ensuring Reasonable Nutrition and Enhancing Physical Activity for Children, Students, Students to Improve Health, Prevent Cancer, Cancer, Cancer and Diabetes and Bronchial Asthma Period 2018 - 2025"*), issued by the government in 2019.<br><br>Decision 1858/QD-BYT (*Instructions for Implementation of Interventions to Prevention of Low Baby Nutritionally in Children Below 5 Years Old for Implementation of the National Target Program for New Rural Building 2021-2025*), issued by the Ministry of Health in 2022.	*Brazil*: The Minister of Health in Brazil has supported new dietary guidelines very different to those in other countries and align with some of the most commonly cited recommendations for healthy eating.<br><br>*Ireland:* Healthy Ireland and Department of Health together have established 'Eat Well' campaign, which provide clear dietary guidelines using the Food pyramid on the maximum daily intake for foods and drinks high in fat, sugar and salt. Food guides have also been created to show people how to use the food pyramid on a daily basis.	**B**<br><br>Policies are not mandatory, mostly provide recommendations.<br><br>There remains a lack of policies to assist consumers to read and understand nutrition contents on food labels and gain general understanding about nutrition.

		Decision 2033/QD-BYT (*National plan communication activities to reduce salt in the diet for prevention and control of hypertension, stroke, and other non-communicable diseases, period 2018-2025*) issued by MOH in 2018, emphasizes the detrimental health effects of high salt consumption among the population and aims to increase awareness and promote behavior change in reducing the levels of salt intake in meals consumed at home and in food outlets. To achieve this goal, the plan incorporates various communication activities, including the distribution of leaflets, propaganda, posters, nutrition advice and guidance for patients, and mass media campaigns.<br><br>Decision 5924/QD-BYT (I*mplementation plan of the Vietnam health program in the 2021-2025 period*), issued by MOH in 2021, aims to promote the Vietnam Health Program by using health days and events of the year to disseminate the program's contents. The focus of the program is on ensuring proper nutrition to prevent non-communicable diseases and includes promoting the role of nutrition labels, encouraging the consumption of vegetables and fruits, reducing the consumption of sugary drinks, fats, and salt, and promoting the use of locally available foods in the Vietnamese eating style.<br><br>Decision 02/QD-TTg (*Nutritional strategy for 2021-2030 and vision to 2045*) issued by the Government on 05 January 2022, provides an orientation on strengthening communication activities through mass media that suitable for each region, each target group in order to improve understanding and practice of good nutrition and encourage the consumption of diverse, healthy and nutritious food among the population. The policy stresses the need to strengthen cooperation between schools, families, and society to form healthy lifestyles and consumption choices for proper nutrition. The policy further extends its target group for communication activities to policy making advocacy group in order to incorporate nutrition content into strategies, programs, schemes and implementation plans at all levels.		

		Decision 1294/QD-BYT (A*ction plan to implement the national strategy on nutrition until 2025*), issued by MOH in 2022 presents an action plan to implement the national nutrition strategy until 2025, with a focus on enhancing public awareness on healthy diets and nutrition for the prevention of non-communicable diseases. The policy outlines several communication activities to be undertaken to achieve this goal, including educating different populations on how to read and understand food labels, the use of food fortified with micronutrients, and the reduction of sugar, salt and trans-fat consumption. The plan advocates for the use of diverse communication tools (such as videos, TV programs, content marketing, and nutrition consulting software) and platforms (social media, TV, radio, training courses, events, etc.) to effectively disseminate information to various target groups, including children, adolescents, and the elderly. Additionally, the policy stresses the importance of improving communication capacity for health education and communication staff at the center level and supporting provincial Centers for Disease Control in enhancing nutrition communication capacity at the local level.		

Food prices and affordability

Food prices

**Indicators**	**Definitions and scope**	**Evidence of Vietnamese Government actions**	**International Best Practice Examples (Benchmarks)**	**Final Score and Justification**
**IND5.1.1 Taxes on healthy foods are minimized** to encourage healthy food	Includes exemptions from excise tax, ad valorem tax or import duty. Includes differential	Law 13/2008/QH12 (*Value added tax law*), issued by the National Assembly in 2008, allows tax exemption for agricultural cultivated products, livestock, and aquaculture products, which have not been processed into other products or have only undergone preliminary processing, are produced and sold by organizations or individuals themselves, and at the importing stage.	*Australia:* Goods and services tax (GST) exemption exists for basic foods (including fresh fruits and vegetables).<br><br>*Tonga:* In 2013, as part of a broader package of fiscal measures, import duties were lowered from 20% to 5% for	**A**<br><br>There are exemption policies on VAT for local produced products. VAT and

choices (e.g. low or no sales tax, value-added or import duties on fruit and vegetables).	application of excise tax, ad valorem tax or import duty. Excludes subsidies (see IND5.2.1).	Decision 376/QD-TTg (*Approval of the national strategy for preventing and controlling cancers, cardiovascular disease, diabetes, chronic obstructive pulmonary disease, bronchial asthma, and other non-infectious diseases during the 2015-2025 period*), issued by the government in 2016, suggests implementing financial policies to encourage the production and consumption of healthy products. No further documents were found related to these financial policies.<br><br>**The documents below could indirectly contribute to this, but they tend to focus on production incentives, including for safe fruits and vegetables.**<br><br>Decree 129/2022/ND-CP (Vietnam's special preferential import tariff schedule for the implementation of the Regional Comprehensive Economic Partnership Agreement for the period 2022 - 2027) signed between the Government and the Vietnam Trade Agreement trade (ACFTA, EVFTA, AKFTA, CPTPP ...) stipulates a preferential import tax of 0% for vegetable, tuber, and fruit products; some nuts and fresh animal meat (See import and export tariff 2023)<br>Decision 222/2006/QĐ-UBND (*Regulations on encouraging investment and trading in safe vegetables and clean food in Hanoi city*), issued by Hanoi People's Committee in 2006, provides investment incentives, in the form of corporate income tax deductions, for investments focused on producing, processing and preserving safe vegetables and clean food, as outlined in Decree 164/2003/ND-CP and its subsequent amendment (Decree 152/2004/ND-CP), which details the implementation of the Law on Corporate Income Tax.<br><br>The Law 106/2016/QH13 (*Amendments to some Articles of the Law on Value-added tax, the law on special excise duty, and the law on tax administration*), issued by the National Assembly in 2016 provides a tax deduction on value added tax for agricultural, livestock, and aquaculture products that are either unprocessed or have only undergone normal preliminary processing, and are produced and sold directly by individuals or organizations themselves.<br><br>Resolution 101/2023/QH15 dated June 24, 2023, of the National Assembly on the 5th session, 15th National Assembly. Accordingly, reducing the value added tax rate by 2% in 2023, applicable to groups of goods and services that are currently applying the value added tax rate of 10% to 8%, effective from July 1 to December 31, 2023.	imported fresh, tinned or frozen fish in order to increase affordability and promote healthier diets.<br>*Fiji*: Excise duties have been removed and import taxes reduced on imported fruit, vegetables and legumes.<br>*Poland*: The rate of GST is lower for unprocessed and minimally processed foods.	import tax exemptions are applied for fresh fruit and vegetables and meats imported to Vietnam as a result of a trade agreement to encourage availability and consumption of healthy products.
**IND5.1.2** Excise **taxes on unhealthy foods** (e.g.	Includes differential application of excise tax, ad	Decision 376/QD-TTg (*Approval of the national strategy for preventing and controlling cancers, cardiovascular disease, diabetes, chronic obstructive pulmonary disease, bronchial asthma and other non-communicable diseases during the 2015-2025 period*), issued by the government in 2016, recommends	*UK*: The Government introduced a sugar tax on the soft drinks industry in 2017. Drinks with a total sugar content above 5g/100ml are taxed at 18 pence per	**C**<br><br>The issue is acknowledged,

sugar-sweetened beverages, foods high in nutrients of concern) are in place and increase the retail prices of these foods to discourage unhealthy food choices.	valorem tax or import duty on high calorie foods or foods that are high in nutrients of concern	researching and proposing suitable tax rates for products that are considered unhealthy, to limit their use.<br>Decision 02/QD-TTg (National Strategy on Nutrition for the period 2021-2030 with a vision to 2045) issued in 2022 by the Prime Minister, recommending the imposition of excise tax on sugary drinks.<br>Decision 1092/QD-TTg (*Approval of Vietnam Health Plan*), issued by the government in 2018, and Decision 5924/QD-BYT (*Approving the implementation plan of the Vietnam health program in the 2021-2025 period*), issued by MOH in 2021, recommend appropriate tax increases to limit the consumption of sugary drinks, processed foods, food additives, and other products with health risks, particularly those marketed to children.<br><br>Decision 1294/QD-BYT (Issuing an a*ction plan to implement the national strategy on nutrition until 2025*), issued by MOH in 2022, and decision 02/QD-TTg (*Approving the national nutrition strategy for the 2021-2030 period with a vision toward 2045*), issued by the government in 2022, both recommend imposing special consumption tax on sugary drinks.<br><br>Decision 155/QD-TTg dated January 29, 2022, of the Prime Minister *approving the National Plan to prevent and combat NCDs and mental health disorders for the period 2022 - 2025*. Including the content of reviewing and completing Improve policies to ensure nutrition for people: "regulations on mandatory nutrition labelling on foods to disclose information on salt, sugar, fat and other related ingredients; tax policy on sugary drinks.	Liter and drinks with more than 8g/100ml will be taxed at 24 pence per Liter.<br>*Qatar:* A 50% ad valorem tax on carbonated drinks with added sugar, sweeteners or flavors. 100% tax on energy drinks.<br>*South Africa:* A sugary beverage levy of 2.1 cents per gram of sugar applies to sugary beverages that exceed 4g per 100mL.	with a few policy documents mentioning the need to impose a special tax on sugary drinks and unhealthy foods, but no clear regulations were found.

Food affordability

**Indicators**	**Definitions and scope**	**Evidence of Vietnamese Government actions**	**International Best Practice Examples (Benchmarks)**	**Final Score + Justification**
**IND5.2.1** The intent of existing **subsidies** on foods is to favour healthy rather than unhealthy foods	Includes agricultural input subsidies, such as free or subsidized costs for water, fertilizer, seeds, electricity or transport (e.g. freight) where those subsidies specifically target healthy foods.<br>Includes programs that ensure that farmers receive a certain price for their produce to encourage increased food production or business viability.	The Law 11/2012/QH13 (*Law on prices*), issued by the National Assembly in 2012, includes price stabilization measures for salt, milk, sugar and rice. It does not favor healthy food items over unhealthy ones.<br><br>Resolution 34/NQ-CP (*Ensuring National food security toward 2030*), issued by MARD in 2021, affirms the need to subsidize rice for ethnic minority and mountainous households and in	*Singapore*: Manufacturers can tap into non-health related government funding for productivity and innovation to improve logistics and efficiency in supply of healthier oils and healthier staples, with a view to making prices competitive.<br><br>*Ireland:* Scheme of Aid for Producer Organisations in the Fruit and	**D**<br><br>The issue is not acknowledged. Current subsidies do not have a specific focus on promoting the consumption of nutritious and healthy foods. In fact, some

	Includes grants or funding support for food producers (i.e. farmers, food manufacturers) to encourage innovation via research and development where that funding scheme specifically targets healthy food.<br>Includes funding support for wholesale market systems that support the supply of healthy foods.<br>Includes population level food subsidies at the consumer end (e.g. subsidizing staples such as rice or bread for flood impacted population).<br>Excludes incentives for the establishment of, or ongoing support for, retail outlets, including greengrocers, farmers' markets, food co-ops, etc.<br>Excludes subsidized training, courses or other forms of education for food producers - Excludes the redistribution of excess or second grade produce.<br>Excludes food subsidies related to welfare support.<br>Should be in line with population nutrition goals related to the prevention of obesity and diet related NCDs (e.g. reducing intake of nutrients of concern and should not be related to micronutrient deficiencies).	general for people in areas suffering from hunger due to the impact of natural disasters and epidemics.<br><br>Decision 01/2012/QD-TTg (on some policies supporting the application of the method of good agricultural practices to agriculture, silviculture and aquaculture) stipulates a number of support policies for individuals, organizations, and households that produce and process agricultural products applying VietGap, including 100% of the funding for basic surveys, topographic surveys, and sample analysis. country, 50% of total investment capital for construction and infrastructure improvement; One-time financial support for product safety certification.<br><br>Decree 109/2018/ND-CP (organic agriculture) regulate policies to encourage the development of organic agriculture, including priorities for applying issued policies; Support 100% of funding to identify regions and areas eligible for organic production; One-time support of 100% of the funding for issuance of Certificates of product compliance with TCVN on organic agriculture.<br><br>Circular 555/QD-BNN-TT (*scheme for restructuring Vietnam's rice industry by 2025 and 2030*), issued by MARD in 2021, continued to put focus on supporting rice production (food sovereignty and food security perspectives) and also plans to mobilize national reserves of rice to support people affected by natural disasters.	Vegetables Sector provides EU aid to producers grouping, on a voluntary basis, to form recognized Producer Organisations (POs) and implement approved operational programmes that improve the quality and efficiency of operations and achieve other objectives such as improving competitiveness and market orientation of the sector, reducing fluctuation in producers' incomes; increasing the consumption of fruit and vegetables in the community; maintain and protect the environment.	price stabilization measures have been observed to apply to products that are generally considered unhealthy, such as salt and sugar (among the nutrients of concern).

Food availability and accessibility

Food availability

Indicators	Definitions and scope	Evidence of Vietnamese Government actions	International Best Practice Examples (Benchmarks)	Final Score + Justification
**IND6.1.1** Existence of **policies** to encourage food stores to **promote the in-store availability** of healthy foods and to limit the in-store availability of unhealthy foods.	Food stores include supermarkets, convenience stores, (including 'general stores' or 'mom&pop stores'), greengrocers, wet markets, vending machines, and other specialty food retail outlets.<br>Support systems include guidelines, resources, or expert support.<br>Includes all settings with food retail stores, including but not exclusive to; train stations, venues, facilities or events frequented by the public etc.<br>Excludes settings owned or managed by the government.<br>Includes the strategic placement of foods and beverages in cabinets, fridges, on shelves or near the cashier - Includes the use of signage to highlight healthy options or endorsements (such as traffic lights or a recognized healthy symbol).<br>Includes offering healthier food and drink products or changing the menu or store layout to offer healthier options.<br>Includes decreasing the offer of unhealthy food and drink products.<br>Excludes reformulation and labelling in relation to nutrients of concern	Decision 222/2006/QĐ-UBND (*Regulations on encouraging investment and trading in safe vegetables and clean food in Hanoi city*), issued by Hanoi People's Committee in 2006, provides mechanisms for encouraging investment and trading in safe vegetables and clean food.<br><br>Decision 885/QD-TTg (*Organic Agriculture Development Project 2020 - 2030*), issued by the government in 2020, encourages supermarkets and convenience stores businesses to incorporate organic agricultural products into their distribution systems.<br><br>In the same lines, Decision 272/QĐ-BCT (*Approving the Project on commercial development planning in the Red River Delta until 2020, with a vision to 2030, issued by the Minister of Industry and Trade*), issued by MOIT in 2015, and Decision 9762/QĐ-BCT (*Commercial development planning in the Southeast region to 2020, orientation to 2030*), issued by MOIT in 2015, aim to facilitate and incentivize shopping centers, supermarkets, and food chain stores in urban areas to source directly from rural producing areas. These policies also encourage manufacturers and suppliers to sell their agricultural products to supermarkets. While the primary objective of these policies is not to improve in-store availability of fresh fruits and vegetables, promoting links between the urban retail sector and rural supply areas could indirectly enhance this aspect.<br><br>In parallel, Vietnamese authorities have been actively promoting the development of the food processing industry, which may not necessarily contribute to promoting the availability of fresh and healthy food options. For instance, Decision 1408/QD-TTg was issued to encourage the development of the seafood processing industry between 2021-2030 and consumptions of aquatic products across modern retail outlets including supermarket chains, convenience stores, clean food stores, domestic tourist attractions. While these policies aim to stimulate economic growth, they also run the risk of promoting the consumption of processed and packaged	*USA:* The Special Supplemental Nutrition Program for Women, Infants, and Children (WIC) requires WIC authorized stores to stock certain healthier products (e.g. wholegrain bread).<br>*The Netherlands*: The National Action plan for vegetables and Fruit is a cooperation of government, industry and civil society organisations. The Goal is to increase the consumption of vegetables and fruits in 3 years (2018-2020) by linking and strengthening existing initiatives. The plan stimulates consumers to eat more vegetables and fruit using the motto 'Go for Color'. As part of 'Go for Color' an in-store experiment has taken place promoting the in-store availability of vegetables and fruit.	**D**<br><br>The issue of encouraging food stores to promote in-store availability of healthy food is not acknowledged. No specific policies were found that directly encourage food stores to promote in-store availability of healthy foods.

		foods over fresh and whole foods, which can negatively impact public health.		
**IND6.1.2** Existence of **policies** to encourage the **promotion and availability** of healthy foods in **food service outlets** and to discourage the promotion and availability of unhealthy foods in food service outlets.	Food service outlets include for-profit quick service restaurants, eat-in or take-away restaurants, and street/sidewalk/casual food catering outlets.<br>Support systems include guidelines, resources, or expert support.<br>Includes settings such as train stations, venues, facilities, or events frequented by the public.<br>Excludes settings owned or managed by the government.<br>Includes the strategic placement of foods and beverages in cabinets, fridges, on shelves or near the cashier - Includes the use of signage to highlight healthy options or endorsements (such as traffic lights or a recognized healthy symbol) and vending machines.<br>Includes modifying ingredients to make foods and drinks healthier or changing the menu to offer more healthy options.<br>Excludes reformulation and labelling in relation to nutrients of concern.	Decision 3073/QĐ-BNN-QLCL (*Approving the project to build and develop a model of a safe agro-forestry-fishery food supply chain nationwide*), issued by MARD in 2013, recommends “the mobilization of distributors, restaurants, hotels, and collective kitchens to select agricultural, forestry, and fishery products from business establishments participating in the safe chain model”. Although not directly related to the indicator, this decision could have indirect positive effects on the availability of fresh and healthy foods in food service outlets.<br><br>Decree 98/2020/ND-CP (*Prescribing penalties for administrative violations against regulations on commerce, production and trade in counterfeit and prohibited goods, and protection of consumer rights*), issued by the government in 2020, prohibits the sale of alcohol and beer through vending machines and imposes a fine ranging between VND 3,000,000 and 5,000,000 for non-compliance with this regulation. Although not directly related to discouraging the promotion and availability of unhealthy foods in food service outlets, this example demonstrates the possibility of implementing laws that prohibit the sale of certain categories of food.	_Singapore_: The Healthier Hawker program in Singapore supports food vendors to offer healthier options and identifies these with ‘Healthier Choice Symbol Identifiers’.<br>_San Francisco (US):_ The city implemented the Health Food Incentives Ordinance, which bans restaurants and fast-food chains to give incentive items with children’s meals, unless the meals meet nutrition standards.<br><br>_France_: Unlimited offers of sweetened beverages have been banned in restaurants and facilities with children under 18 years.	**D**<br><br>The issue of encouraging food service outlets to promote in-store availability of healthy food is not acknowledged. No relevant content found in the existing policy documents about this topic, however there are some policy measures that might indirectly contribute to it.

Food accessibility

Indicators	Definitions and Scope	Evidence of Vietnamese Government actions	International Best Practice Examples (Benchmarks)	Final Score and Justification

**IND6.2.1 Zoning laws and policies** to place **limits on the density** or placement of quick serve restaurants or other outlets selling mainly unhealthy foods in communities and/or access to these outlets (e.g. opening hours).	Includes the consideration of public health in national and subnational plans that guide the policies, priorities, and objectives to be implemented at the local government level through their planning schemes.<br>Includes a national/sub-national guideline that sets the policy objective of considering public health when reviewing and approving fast food planning applications.<br>Includes limitations on access to unhealthy food outlets (i.e. opening hours).	No relevant content found in the existing policy documents directly addressing the indicator.<br><br>Directive 08/CT-UBND (*Strengthening management of food service and street food business activities in Hanoi*), issued by Hanoi People's Committee in 2016, aims to improve the management of food service and street food businesses in Hanoi by designating specific streets for these activities and ensuring that the infrastructure and public services meet food safety standards. It does not focus on improving the availability of healthy food, but rather on addressing food safety concerns.	*South Korea*: In 2010, the Special Act on Children's Dietary Life Safety Management established the creation of 'Green Food Zones' around schools, banning the sale of foods (fast food and soda) deemed unhealthy by the Food and Drug Administration of Korea within 200 meters of schools. In 2016, Green Food Zones existed at over 10,000 schools.<br>*UK*: Around 15 local authorities have developed "supplementary planning documents" on the development of hot food takeaways. The policies typically exclude hot food takeaways from a 400m zone around the target location. All policies include secondary schools, some policies also include primary schools, parks and youth centers.<br>*Detroit (USA)*: the zoning code prohibits the building of fast-food restaurants within 500ft. of all elementary, junior and senior high schools	**D**<br><br>The issue is not acknowledged. No relevant content found in the existing policy documents.
**IND6.2.2 Zoning laws and policies to encourage** the development of outlets selling **fresh fruit and vegetables** and/or to increase access to these outlets (e.g. opening hours, frequency i.e. for markets).	Outlets include supermarkets, produce markets, farmers' markets, greengrocers, food cooperatives.<br>Includes fixed or mobile outlets. Excludes community gardens, or backyard gardens.<br>Includes policies that support local governments to reduce license or permit requirements or fees to encourage the establishment of such outlets.<br>Includes National/sub-national level policies to streamline and standardize planning approval processes or reduce regulatory burdens for these outlets.<br>Includes actions to improve access to healthy food outlets (i.e. opening hours, frequency (for markets)).	No relevant content found in the existing policy documents directly addressing the matter of the indicator.<br><br>Decision 6481/QD-BCT (Master plan on development of the national market network up to 2025, with a vision to 2035) issued in 2015 by the Minister of Industry and Trade set out the goal of developing a wholesale market network to trade most of the agricultural products produced by farmers (specialty products, staples, specialized and concentrated products, etc.), and at the same time ensure the supply of agricultural products for the local retail network.<br><br>Decision No. 645/QD-TTg (Approving the National E-commerce Development Master Plan for the period 2021 - 2025) dated May 15, 2020, of the Prime Minister with the goal of supporting and promoting the application widely used e-commerce in businesses and communities. At the same time, it also	*US*: Congress 'Healthy Food Financing Initiative' provides grants for assistance to attract healthier retail outlets to undeserved areas.<br><br>*New York City (US)*: The 'Green Cart Permit' in New York reduces restrictions on zoning requirements for fresh fruit and vegetables vendors.<br><br>*Scotland*: A 'Healthy Living Neighborhood Shops' project worked with convenience stores to improve the availability of healthy items.	**D**<br><br>The issue of encouraging the development of outlets selling fresh fruit and vegetables or increasing their accessibility has not been acknowledged. There is only a policy on planning and developing a network of wholesale markets for agricultural products to ensure the supply of outlets selling fresh fruit and vegetables.

	Includes the provision of financial grants or subsidies to outlets	contributes to increasing consumer access to agricultural products and goods.<br><br>Decision 1034/QD-BTTTT (Plan to support bringing agricultural production households to the e-commerce platform, promoting agricultural and rural digital economic development) dated July 21, 2021, of the Ministry of Information and Communications with the goal of supporting production households to promote the consumption of agricultural products on e-commerce platforms, contributing to increasing access to agricultural goods through e-commerce platforms.		

6. Supp. Mat. #6: Scoring system for indicators

Score	Scoring criteria	**Theoretical example 1**<br>IND1.2.1 Salt content targets / standards / restrictions in industrially processed foods
A	The existing policy documents consist of a comprehensive legal framework that acknowledges the significance and necessity of addressing the indicator/issue; **and** provide full comprehensive guidance and specific measure on how to approach it; **and** include a focus on the implementation and enforcement of these policies, including clarifying the roles and responsibilities of the entities involved in policy implementation.	The issue of high salt content in processed food is acknowledged; **and** there are targets set to reduce salt content in processed food; **and** there are clear standards specifying the maximum salt content of processed food; **and** responsibilities for controlling and testing salt content in processed food are established, along with penalties in case of violations of the law.
B	The existing policy documents acknowledge the significance and necessity of addressing the indicator/issue; **and** provide guidance and specific measures on how to approach it; **however**, they do not comprehensively address all the dimensions related to the indicator/issue (i.e., there are still some gaps); **and/or** lack of a focus on the implementation and enforcement of these policies.	The issue of high salt content in processed food is acknowledged; **and** there are targets set to reduce salt content in processed food; **however**, there are no standards specifying the maximum salt content of processed food, **and** there is no clarity regarding who is controlling salt content in processed food.
C	The existing policy documents acknowledge the significance and necessity of addressing the indicator/issue; **however,** they lack guidance and specific measures on how to approach it.	The issue of high salt content in processed food is acknowledged; **however**, there are no specific targets, standards, or restrictions for limiting salt content in processed food.
D	The existing policy documents do not acknowledge the significance and necessity of addressing the indicator/issue.	The issue of high salt content in processed food is not recognized as a concern in any of the policy documents.

Score	**Theoretical example 2**<br>IND3.1.1 Policies to restrict exposure and power of promotion of unhealthy foods through broadcast media (TV, radio) and online/social media.	**Theoretical example 3**<br>IND6.2.2 Zoning laws and policies to encourage the development of outlets selling fresh fruit and vegetables and/or to increase access to these outlets (e.g., opening hours, frequency, i.e., for markets).
A	The issue of restricting the promotion of unhealthy foods through broadcast media and online/social media is acknowledged; **and**, there are regulations in place, to limit such promotions; **and**, there is a strict surveillance and control system in place, ensuring a healthier media environment.	Existing policies acknowledge the need for zoning laws to encourage the development of outlets selling fresh fruit and vegetables in underserved areas; **and**, there are specific regulations to facilitate the setup of healthy outlets in designated food priority areas; **and** an entity has been established within the Ministry of Health to monitor and evaluate the progression of healthy food outlet in underserved areas.
B	The issue of restricting the promotion of unhealthy foods through broadcast media and online/social media is acknowledged; **and**, there are regulations in place, to limit such promotions; **however**, there is a limited surveillance and control, allowing for some promotion of unhealthy foods to persist.	Existing policies acknowledge the need for zoning laws to encourage the development of outlets selling fresh fruit and vegetables in underserved areas; **and**, there are specific regulations to facilitate the setup of healthy outlets in designated food priority areas; **however**, there is no system and tools to monitor the changes and evaluate the progression of healthy outlets in underserved areas.
C	The issue of restricting the promotion of unhealthy foods through broadcast media and online/social media is acknowledged; **however**, there are no regulations in place, resulting in limited restrictions on the exposure and power of such promotions.	Existing policies acknowledge the need for zoning laws to encourage the development of outlets selling fresh fruit and vegetables in underserved areas. **However**, they do not clearly define what is a healthy outlet, **and** provisions for facilitating the setup of healthy outlets are still incomplete.
D	The issue of restricting the promotion of unhealthy foods through broadcast media and online/social media is not recognized as a concern in any of the policy documents.	Existing policy documents do not recognize the importance of zoning laws and policies to promote outlets selling fresh fruit and vegetables in order to increase access to healthy foods.

7. Supp. Mat. #7: List of policy recommendations

No.	Recommendation (full name)	Short name
1	Develop guidelines and training programs on food safety that align with internationally recognized standards and the needs of organizations and individuals in the food system.	**Guidelines and training programs for food safety**
2	Develop processes and plans for food safety inspections at the district and commune/ward levels and allocate appropriate financial resources for implementation.	**Local-level plans for food safety inspections**
3	Establish a unified food safety management organization that consolidates expertise from the health, agriculture, and industry and trade sectors.	**Unified organization for food safety management**
4	Establish a legal framework and mandatory timeline for implementing Good Manufacturing Practice (GMP), Good Agricultural Practice (GAP), Good Hygiene Practice (GHP), Hazard Analysis and Critical Control Points (HACCP), and other advanced food safety management systems equivalent to international standards for food producers, processors, and businesses.	**Legal framework for advanced food safety standards**
5	Allocate appropriate state budget and mobilize additional external resources for investment in safe, sustainable food production activities, prioritizing investment in organic and ecological agriculture.	**Investment in safe and sustainable food production**
6	Establish specific standards for the maximum content of concerning nutrients (salt, sugar, trans fats, saturated fats) in processed foods, in accordance with FAO-WHO and CODEX ALIMENTARIUS standards.	**Nutrient content standards for processed foods**
7	Develop, clarify, and promote definitions of healthy and unhealthy diets, as well as foods that are beneficial and not beneficial to health, in the context of Vietnam.	**Definitions for healthy and unhealthy diets**
8	Update and revise nutritional standards for supplementary foods for children under 36 months old, incorporating recommendations from WHO, UNICEF, and ASEAN.	**Updated nutritional standards for children's foods**
9	Establish principles, processes, and clearly define responsibilities for systematic inspection and control of nutritional content (especially concerning nutrients) in processed foods.	**Processes for inspection and control of nutritional content**
10	Establish principles, processes, and clearly define responsibilities for systematic inspection and control of nutritional content, and issue Vietnamese standards specifying the recommended daily limits for sugar, salt, and fat.	**Recommended daily limits for sugar, salt, and fat**
11	Develop policies to support food retailers that provide fresh and safe food at affordable prices.	**Support mechanisms for fresh and safe food retailers**
12	Develop recommendations and guidelines for food retailers to encourage them to reduce salt, sugar, and fat in prepared foods.	**Guidelines to reduce salt, sugar, and fat in prepared foods**
13	Develop national criteria and standards to classify, certify, and evaluate food retailers based on dietary and health guidelines.	**Standards for certifying and evaluating food retailers**
14	Develop nutritional standards, minimum and maximum nutritional limits for institutional establishments providing regular meals (e.g., elderly care facilities, children's care/protection centers, schools, canteens, and catering companies).	**Nutritional standards for meals in institutional settings**
15	Develop and issue mandatory regulations to encourage the selection of health-promoting foods in public schools and other public institutions to limit the provision and sale of industrial and ultra-processed foods.	**Mandatory regulations promoting healthy foods in schools**
16	Develop specific regulations on marketing and trading unhealthy foods in school canteens and other public facilities.	**Marketing restrictions on unhealthy foods in schools**
17	Develop and implement appropriate training programs for management personnel, staff in charge of institutions, educators, healthcare professionals, canteen managers, and communicators to ensure the effective implementation of regulations for selecting health-promoting foods.	**Training programs for health-promoting food interventions**
18	Develop policies to encourage businesses to provide healthy, nutritious diets in the workplace.	**Policy incentives for nutritious diets in workplaces**

19	Develop policies to restrict and/or ban the advertising and marketing of unhealthy foods to children under 15 years old across all media and in child-centered environments.	**Restrictions on advertising unhealthy foods to children**
20	Develop regulations to ban the advertising and marketing of breast milk substitutes for children under 36 months old.	**Prohibition of advertising for breast milk substitutes**
21	Develop regulations to prohibit individuals and organizations from using imagery that appeals to children under 15 years old on the packaging of unhealthy food products.	**Ban on child-targeted packaging for unhealthy foods**
22	Develop additional food labeling regulations to distinguish between health-promoting and non-health-promoting foods on product packaging, with a specific focus on salt, sugar, trans fats, and saturated fats.	**Food labeling regulations focusing on the nutrients of concern**
23	Design a national front-of-pack nutrition label for pre-packaged foods to mandate the disclosure of calorie content and key nutrient levels, including the maximum daily recommended intake.	**National front-of-pack nutrition labels**
24	Develop a comprehensive communication strategy to promote and effectively implement Circular No. 29/2023/TT-BYT issued by the Ministry of Health, providing guidance on labeling ingredients and nutritional values on food labels.	**Communication strategies for food labeling regulations**
25	Develop guidelines to help consumers understand and use nutritional information on food labels, enabling consumers to make informed decisions toward choosing healthier foods.	**Guidelines for interpreting nutritional information on labels**
26	Develop national rules and standards for regulating nutrition and health claims on food packaging, including a penalty system for false or unfounded claims.	**Regulations for nutrition and health claims on packaging**
27	Establish a regulatory body responsible for overseeing, evaluating, and enforcing compliance with food label claims.	**Oversight body for enforcing food label claims**
28	Develop policies to encourage food service establishments to display nutritional information such as energy content, total salt, sugar, trans fats, and saturated fats on their menus.	**Display of nutritional information in food service menus**
29	Establish guidelines for selecting nutritious foods and reducing choices of unhealthy foods in daily meals (based on a list of health-promoting foods recommended for consumption and unhealthy foods that should be consumed in moderation according to individual age groups).	**Guidelines for selecting nutritious foods**
30	Intensify communication campaigns to raise awareness about choosing, preparing, and consuming health-promoting foods.	**Public communication campaigns on health-promoting foods**
31	Develop policies to encourage food retailers to raise consumer awareness and promote the consumption of health-promoting foods through effective communication strategies and materials.	**Strategies for promoting healthy foods by retailers**
32	Propose implementing an excise tax on sugary drinks according to recommended thresholds, and apply different tax rates based on sugar content.	**Excise tax policies on sugary drinks**
33	Propose implementing an excise tax on processed and ultra-processed foods that exceed recommended thresholds for concerning nutrients.	**Excise tax on processed and ultra-processed foods**
34	Develop policies to encourage the inclusion of fresh and health-promoting foods in subsidy programs for national and local emergency situations.	**Policies for including fresh foods in emergency programs**
35	Develop policies to encourage food retailers to provide health-promoting foods at affordable prices.	**Pricing strategies to ensure availability of healthy foods**
36	Develop policies to encourage food retailers to adopt signage systems to guide consumers in choosing health-promoting foods.	**Signage systems for promoting healthy food choices**
37	Integrate public health issues into national and local plans, including infrastructure development and providing financial incentives for the establishment and development of health-promoting food retailers, and prioritize investment in upgrading infrastructure for food storage and preservation.	**Integration of public health priorities in development plans**
38	Establish "green food zones" around schools to limit the sale of unhealthy foods.	**Establishment of green food zones around schools**

8. Supp. Mat. #8: Ranking system for policy recommendations

A: Relevance to the Vietnamese context. These following criteria assess the policy recommendations' relevance and appropriateness to the Vietnam's socioeconomic, cultural, and institutional context. They consider the alignment of the specific recommendations with national priorities, economic dynamics, and socio-cultural norms, and examines whether the policies are acceptable across societal sectors.
Specific criteria to be scored:

- **A1. Institutional acceptability** (at the national and sub-national levels).
  - Definition: Evaluates the extent to which a policy recommendation's objectives and design respond to the country and institutional orientation, agenda, and strategic directions at both national and sub-national levels and continue to do so if circumstances change. Relevance assessment involves looking at differences and trade-offs between different priorities or needs. It requires analysing any changes in the context to assess the extent to which the policies can be implemented/adapted to remain relevant.
  - Question: To what extent does the proposed policy align with and reflect the country and institutional strategic goals, policies, and priorities?

  Note: "respond to" means that objectives and design of the policy are sensitive to the economic, environmental, equity, political economy, and capacity conditions in which it takes place.

Example:
Score 0 = A policy recommendation that is in direct conflict with national or sub-national strategic goals, policies, and priorities, showing no compatibility with current institutional directions or legal frameworks.
Scores 1-3 = a policy recommendation that, although aligning slightly with some government goals, largely conflicts with key priorities or face significant institutional resistance.
Score 4-6 = a policy recommendation that aligns fairly well with government priorities but has notable issues that limit its institutional viability.
Scores 7-9 = a policy recommendation that aligns closely with government priorities, albeit with minor reservations or needed adjustments.
Score 10 = a policy recommendation fully aligned with government strategies, having widespread political support and alignment with the existing legal framework.

- **A2. Socio-cultural and economic acceptability**.
  - Definition: Assesses the alignment of policy recommendations with local economy and market dynamics as well as local customs, practices, and social norms that characterize the Vietnamese society.
  - Question: To what extent does the proposed policy align with local economic and socio-cultural dynamics of Vietnam?

Example:
Score 0 = a policy recommendation that entirely disregards or conflicts with Vietnamese customs and social norms, rendering it ineffective and culturally insensitive across the country.

Scores 1-3 = a policy recommendation that resonates with specific cultural practices or social norms of particular regions or communities in Vietnam but is inapplicable or potentially controversial in others due to significant cultural mismatches or insensitivities.
Score 4-6 = a policy recommendation that demonstrates an average level of sensitivity towards Vietnamese socio-cultural norms and has the potential to be accepted in several regions, albeit facing considerable challenges in broader application.
Scores 7-9 = a policy recommendation that shows a strong alignment with the socio-cultural context of most Vietnamese communities, suggesting it can be effectively integrated nationwide with minor modifications to address local cultural nuances and ensure broader acceptability.
Score 10 = a policy recommendation that is deeply rooted in an understanding of and respect for Vietnamese cultural practices and social norms, ensuring its effectiveness and widespread acceptability across all regions of the country without necessitating significant adjustments.

B: Feasibility of implementation. The following criteria evaluate the practicality and feasibility of implementing and executing the recommended policies across Vietnam, from national down to provincial levels. It considers the legal and financial aspects necessary for successful policy adoption and widespread application.
Specific criteria to be scored:

- **B1. Compatibility with existing policies and legal framework**.
    - Definition: Assesses whether the policy can be integrated within existing legal and regulatory frameworks without necessitating significant changes or rendering adverse impacts on the current framework.
    - Question: To what extent does the proposed policy align with existing legal and regulatory frameworks, and how easily can it be integrated in it?

Example:
Score 0: a policy recommendation that is fundamentally incompatible with existing policies and legal framework, making its application impractical and potentially disruptive.
Scores 1-3: a policy recommendation that necessitates substantial modifications to an effective current policy or could adversely affect the functionality of existing legal frameworks, thus complicating its implementation..
Score 4-6: a policy recommendation that generally aligns with existing legislation but requires changes that may affect the smooth functioning or the outcomes of current policies, thereby presenting challenges to its implementation and anticipated effectiveness.
Score 7-9: a policy recommendation that is mostly compatible with the existing legal framework, indicating it can be integrated with relative ease. However, its applicability might be limited to specific sectors or conditions, requiring targeted adjustments to fully realize its benefits without undermining the coherence of the existing legal system.
Score 10: a policy recommendation that is fully aligned with the existing and coming changes of policies and legislations at both national and subnational levels in Vietnam, indicating a high degree of feasibility for implementation without necessitating significant changes to the existing legal framework.

- **B2. Resources needs/ requirements for implementation.**

- Definition: Assesses the need of human, economic, and financial resources that the policy's implementation would demand, including both initial investments and ongoing operational costs.
- Question: To what extent does the proposed policy require significant human, economic, and financial resources for successful implementation compared to other alternatives or within the context of available budgets and other relevant resources?

Example:
Score 0: a policy recommendation deemed impractical due to overwhelming initial investment and ongoing operational costs.
Score 1-3: a policy recommendation that requires significant investments and resources – funding, time, expertise, etc. - to be implemented, challenging its viability.
Score 4-6: a policy recommendation that is applicable with relatively low investment, but with expected high operational costs (or with relatively high investments, but low operational costs) that are likely no to be covered by public funds.
Score 7-9: a policy recommendation with relatively low investments and operational costs, likely fitting within existing budgets but with some constraints..
Score 10: a policy recommendation that is effortlessly adoptable, well within financial limits and resource availability.

C: Potential for achieving nutritional and health outcomes and impact: This category considers the expected health and nutritional outcomes of the policy, evaluating its potential for significantly improving public health.

- Definition: Assesses the extent to which the recommended policy potentially generates significant positive or negative, intended or unintended, higher-level effects on health and nutrition to the population in Vietnam.
- Question: To what extent does the proposed policy hold potential for generating positive outcomes and impacts and addressing the current dietary and health challenges faced by the Vietnamese population?

Example:
Score 0: a policy recommendation that, based on evidence, either fails to enhance health outcomes or risks detrimental health impacts.
Score 1-3: a policy recommendation with limited evidence suggesting modest improvements in specific health and nutritional challenges, potentially affecting only a narrow segment of the population or having possible unintended negative consequences.
Score 4-6: a policy recommendation supported by evidence for having a positive impact on a range of health and nutritional issues, benefiting a specific group but with possible unintended effects.
Score 7-9: a policy recommendation evidenced to significantly improve a wide array of nutritional and health outcomes, benefiting a broad section of the population, with careful consideration of and measures to mitigate any unintended negative impacts.
Score 10: a policy recommendation that fully addresses the current and potential dietary and health challenges, significantly contributing to improve nutrition and health outcomes for all, with minimal to no unintended adverse effects.

D: Urgency of implementation: This criterion focuses on the need for timely intervention, ensuring that policies are prioritized based on the urgency of corresponding health issues within the Vietnamese context.

- Definition: Evaluates the criticality and time-sensitivity of implementing the policy recommendation, considering the current public health challenges in Vietnam.
- Question: To what extent is the proposed policy urgent in addressing current health and dietary challenges faced by the Vietnamese population?

Example:
Score 0: a policy recommendation that does not address an immediate or pressing health need, or may even exacerbate current health and dietary challenges.
Score 1-3: a policy recommendation that addresses a moderate health or dietary issue but is not critical to immediate public health strategies.
Score 4-6: a policy recommendation that is timely and aims to address a significant health challenge that has potential to worsen if not addressed soon, though it is not yet at a critical stage. Implementation can be planned within a mid-term horizon without immediate repercussions.
Score 7-9: a policy recommendation targeting a rapidly emerging or worsening public health issue that is significant, and soon to be critical. Implementation within the short to medium term could prevent more severe health outcomes and contain the issue more effectively.
Score 10: a policy recommendation addressing a critical and immediate health challenge that, if not implemented promptly, could lead to severe public health crises. This policy is crucial for immediate action to mitigate a rapidly escalating health threat affecting a large portion of the population.

Additional information on the ranking system:

Numeric scoring system (0-10 Scale): each criterion is scored on a scale from 0 to 10, where 0 represents the lowest score and 10 the highest. This 0-10 scale allows a nuanced analysis of each policy recommendation, enabling detailed differentiation based on the degree to which they meet each criterion.

Composite score index: For each policy recommendation, a composite score index is calculated. This index computes the scores of the various criteria into a single, aggregated metric. These composite scores allow a consistent orderly ranking of all policy recommendations, making the prioritization easier.

9. Supp. Mat. #9: Detailed scores for all criteria across all recommendations

	FE domain	Recommendation short name	# Reco	Average aggregated score ALL CRITERIA	Average aggregated score RELEVANCE	Average aggregated score FEASIBILITY	Average aggregated score IMPACT	Average aggregated score URGENCY
First tercile	#4 Food desirability	Public communication campaigns on health-promoting foods	R30	7.6	7.8	7.3	7.6	7.6
	#1 Food product properties	Definitions for healthy and unhealthy diets	R7	7.5	7.8	7.3	8.2	6.9
	#3 Food marketing and labeling	Communication strategies for food labeling regulations	R24	7.5	7.6	7.3	7.4	7.8
	#3 Food marketing and labeling	Guidelines for interpreting nutritional information on labels	R25	7.4	7.6	6.9	7.5	7.7
	#2 Food outlet properties	Policy incentives for nutritious diets in workplaces	R18	7.3	7.3	7.4	7.2	7.2
	#2 Food outlet properties	Training programs for health-promoting food interventions	R17	7.2	7.5	7.0	7.2	7.1
	#1 Food product properties	Updated nutritional standards for children’s foods	R8	7.1	7.6	6.9	7.0	6.6
	#1 Food product properties	Guidelines and training programs for food safety	R1	7.0	6.9	7.0	7.2	7.1
	#3 Food marketing and labeling	National front-of-pack nutrition labels	R23	7.0	7.3	6.8	6.6	7.2
	#3 Food marketing and labeling	Regulations for nutrition and health claims on packaging	R26	6.9	7.2	6.5	7.1	7.2
	#1 Food product properties	Legal framework for advanced food safety standards	R4	6.8	6.7	6.3	7.5	7.4
	#4 Food desirability	Guidelines for selecting nutritious foods	R29	6.8	7.0	6.6	6.8	6.8
	#1 Food product properties	Nutrient content standards for processed foods	R6	6.8	6.4	6.7	7.7	6.9
Second tercile	#3 Food marketing and labeling	Food labeling regulations focusing on the nutrients of concern	R22	6.8	6.6	6.5	7.2	7.3
	#1 Food product properties	Processes for inspection and control of nutritional content	R9	6.8	6.7	6.7	7.2	6.6
	#2 Food outlet properties	Marketing restrictions on unhealthy foods in schools	R16	6.7	7.0	5.9	7.1	7.0
	#3 Food marketing and labeling	Display of nutritional information in food service menus	R28	6.7	6.8	6.1	7.0	7.1
	#2 Food outlet properties	Nutritional standards for meals in institutional settings	R14	6.6	6.7	6.3	6.9	6.9
	#2 Food outlet properties	Mandatory regulations promoting healthy foods in schools	R15	6.6	6.6	6.4	7.1	6.7
	#6 Food availability and accessibility	Establishment of green food zones around schools	R38	6.6	6.6	6.6	7.2	7.0
	#1 Food product properties	Unified organization for food safety management	R3	6.5	6.5	6.2	7.1	6.7
	#1 Food product properties	Investment in safe and sustainable food production	R5	6.5	6.4	6.3	7.0	6.7
	#5 Food prices and affordability	Excise tax policies on sugary drinks	R32	6.5	6.1	6.3	7.1	7.2
	#1 Food product properties	Local-level plans for food safety inspections	R2	6.4	6.3	6.2	6.8	6.7

	#4 Food desirability	Strategies for promoting healthy foods by retailers	R31	6.4	6.9	5.9	6.6	6.3
Third tercile	#6 Food availability and accessibility	Signage systems for promoting healthy food choices	R36	6.4	6.6	5.9	6.6	6.6
	#1 Food product properties	Recommended daily limits for sugar, salt, and fat	R10	6.4	6.7	6.0	6.5	6.2
	#3 Food marketing and labeling	Restrictions on advertising unhealthy foods to children	R19	6.3	6.3	5.8	6.8	7.1
	#6 Food availability and accessibility	Pricing strategies to ensure affordable healthy foods	R35	6.3	6.5	5.8	6.8	6.5
	#3 Food marketing and labeling	Prohibition of advertising for breast milk substitutes	R20	6.3	6.3	6.2	6.3	6.2
	#3 Food marketing and labeling	Ban on child-targeted packaging for unhealthy foods	R21	6.2	6.2	5.8	6.5	6.5
	#5 Food prices and affordability	Excise tax on processed and ultra-processed foods	R33	6.1	6.0	5.7	6.9	6.4
	#2 Food outlet properties	Guidelines to reduce salt, sugar, and fat in prepared foods	R12	6.0	6.0	5.7	6.6	6.4
	#3 Food marketing and labeling	Oversight body for enforcing food label claims	R27	6.0	5.9	5.6	6.5	6.6
	#6 Food availability and accessibility	Integration of public health priorities in development plans	R37	6.0	6.4	5.4	6.4	5.9
	#2 Food outlet properties	Standards for certifying and evaluating food retailers	R13	5.8	5.6	5.4	6.5	6.2
	#5 Food prices and affordability	Policies for including fresh foods in emergency programs	R34	5.8	5.6	5.7	5.9	5.9
	#2 Food outlet properties	Support mechanisms for fresh and safe food retailers	R11	5.8	5.6	5.6	6.5	5.8

10. <u>Supp. Mat. #10: Definitions of the food environments domains</u>

1. **Food products properties**. This domain refers to the intrinsic attributes of food products, including their nutritional "*quality, safety, level of processing, and shelf-life"* (Turner et al., 2018). These attributes are fundamental as they play a pivotal role in aligning the food supply with public health objectives. Indeed, food product properties determine the range and quality of the foods made available to the consumers and therefore have a potentially significant effect on dietary patterns and eventually on the nutritional intake and health outcomes of populations.
2. **Food outlet properties**. This domain encompasses the characteristics of food retail and service outlets, including their type (e.g., supermarket, convenience store, wet market), operating hours, location, and the variety of services and foods offered. These attributes are crucial because they directly affect the purchasing patterns of consumers, influencing where and what they can purchase (Glanz et al., 2007; Shaw et al., 2023). Food outlet properties also determine some aspects of the convenience (cf. domain 4) and accessibility (cf. domain 6) of food, shaping consumer behaviors by determining the ease of access to either healthier or less nutritious food options (Turner et al., 2018).
3. **Food marketing**. This domain refers to the promotion and labeling of food products, encompassing "*advertising, branding, sponsorship, promotional activities, labeling*" (Turner et al., 2018), and packaging. Food marketing exerts a powerful influence on consumer knowledge, preferences, and behaviors, shaping food desirability and ultimately guiding food choices (Harris et al., 2009; Cairns et al., 2013). Depending on the nature of the marketed products, food marketing can either support or undermine public health objectives (McGinnis et al., 2006); for instance, food marketing has been pointed out for specifically targeting children and being skewed towards promoting ultra-processed food high in sugar, fat, and salt (Wilks, 2009; Boyland et al., 2022).
4. **Food desirability**. This domain refers to the subjective appeal of food items (Vermeulen et al., 2020), involving both acceptability and convenience. Acceptability includes factors related to food knowledge, preferences, taste, cultural relevance, and health perceptions. Convenience refers to the ease of obtaining, preparing, and consuming food (Herforth and Ahmed, 2015; Downs et al., 2020). Convenience is strongly linked to other multiple domains of the food environment: the food product itself (e.g., ease of preparation, quick consumption, etc.), the food outlet (e.g., quick service, possibility to eat on-site, etc.), or the physical access (e.g., location along a regular commute, close from home or work, etc.) (Bogard et al., 2024). We decided to include convenience under the desirability domain because it captures the consumer's perception of ease and satisfaction in food choice, which is inherently linked to, among others, personal expectations, time constraints, and lifestyle, that play a pivotal role in shaping food desirability.
5. **Food prices and affordability**. This domain combines two interrelated aspects: food prices, which pertain to the supply side, and affordability, which refers to the consumer's purchasing power and subsequent financial ability to access food. Food prices are notably determined by market forces, production costs, and retail practices (and often out of control for consumers -except in informal settings where price 'negotiations' may be possible), while affordability is shaped by the consumer's economic circumstances (Herforth et al., 2020). Together, these factors influence food

choices by determining whether consumers can consistently purchase sufficient, safe, and nutritious food (Hirvonen et al., 2019; Vermeulen et al., 2020).

6. **Food availability and accessibility**. This domain refers to both the physical presence of food (availability) and the ease with which consumers can obtain it (physical accessibility). As noted by Herforth and Ahmed, p. (2015, p. 508) "*availability is the most basic level of the food environment that affects dietary choices. […] as food cannot be consumed if it is not available*". Turner et al., p. (2018, p. 95) further clarify that "*availability precedes accessibility*", meaning that the presence of food is a prerequisite for consumers to access it. Accessibility, here, refers to the physical means through which consumers can reach and acquire food, including considerations such as proximity and transportation.

11. Supp. Mat. #11: Affiliations and expertise of panel members

Expert	Affiliation	Expertise
Expert 1	National Institute of Nutrition, Ministry of Health	Food and nutrition policies, public health
Expert 2	Vietnamese Academy of Agricultural Sciences, Ministry of Agriculture and Rural Development	Agricultural development, food systems
Expert 3	Alive & Thrive	Food security, nutrition interventions
Expert 4	Global Health Advocacy Incubator	Public health, food policies
Expert 5	National Agro-Forestry-Fisheries Quality Assurance Department, Ministry of Agriculture and Rural Development	Food safety standards, quality control
Expert 6	Institute of Policy and Strategy for Agricultural and Rural Development, Ministry of Agriculture and Rural Development	Market analysis, agricultural policies
Expert 7	Department of Market and Industry Research, Ministry of Agriculture and Rural Development	Food trade, market policies
Expert 8	Fruit and Vegetable Research Institute, Vietnamese Academy of Agricultural Sciences	Crop research, sustainable agriculture
Expert 9	Ministry of Health	Public health policies, food regulation
Expert 10	Food Safety and Biotechnology Department, Ministry of Industry and Trade	Food safety, trade regulations
Expert 11	Health Bridge	Public health, nutrition advocacy
Expert 12	Vietnamese National University of Agriculture, Ministry of Agriculture and Rural Development	Agricultural innovation, sustainable farming
Expert 13	Department of Science and Technology, Ministry of Industry and Trade	Food safety, market policies
Expert 14	Food safety Department, Ministry of Health	Public health
Expert 15	Non-Communicable Disease Control Department, Ministry of Health	Public health, nutrition policy
Expert 16	Center for Agrarian Systems Research and Development, Vietnamese Academy of Agricultural Sciences	Rural development, food systems
Expert 17	Vietnam National University	Food governance, policy analysis, food safety
Expert 18	Department of Cooperatives and Rural Development,, Ministry of Agriculture and Rural Development	Social protection, food security

12. Supp. Mat. #12: Detailed findings on policy strengths and weaknesses

Food products properties

Food nutritional quality. As shown in figure 3, indicators related to salt, sugar, and trans-fat content in both processed foods and meals in food outlets generally received low scores. Viet Nam has implemented a limited number of policies concerning the nutritional quality of processed foods, with a primary focus on salt reduction. These efforts (e.g., *Decision 376/QD-TTg* and *Decision 2033/QD-BYT*) include setting salt reduction objectives for the food industry. However, these objectives remain vague, with no precise targets, no standards specifying the maximum salt content of processed food, and no clarity regarding which entity is controlling salt content in processed food. Standards for salt content have also been established in school meals but these standards are mainly guidance documents and are not mandatory.

The regulation of the other nutrients of concern, sugar and trans-fat, is even less developed. While there is an acknowledgment of the need to control sugar content in processed foods and meals (*Decision 376/QD-TTg*), the absence of precise targets and standards remains a significant shortcoming. The issue of trans-fat content in processed food and food service outlets is not addressed, with no formal recognition or regulations in place.

Food safety. In response to persistent food safety challenges (Nguyen-Viet et al., 2017), the Vietnamese government has introduced a series of structural reforms, with the establishment of a national food control system as a main measure. The enactment of the *Food Safety Law 55/2010/QH12* marked a shift towards "modernized" governance in food safety, with decentralized public agencies, standardized procedures, and systematic food safety controls (Pham and Dinh, 2020). This legislation established clearer responsibilities for regulatory bodies involved in overseeing food production, trading, and import/export processes, along with standards and technical regulations. Despite these advancements, significant challenges persist, particularly in the enforcement of food safety regulations. Issues such as limited specialized food safety inspection resources at lower administrative levels and an inconsistent implementation of communication and training initiatives for stakeholders weaken the overall impact of these controls. Strengthening regulatory enforcement and expanding educational outreach on food safety remain essential to address these gaps effectively.

Food outlet properties

Supporting and restricting healthy/unhealthy food retail outlets. The current policy framework does not differentiate between retailers offering healthy and unhealthy food options. Retailers are typically categorized into modern or traditional channels, with national policies generally favoring the development of modern retail outlets (e.g., *Decision 5078/QD-BCT* and *Decision 1163/QD-TTg*). Some of these modern channels (e.g., convenience stores, vending machines) are often more likely to sell unhealthy food products, yet national policies lack specific regulations that would mitigate this issue.

Likewise, policies aimed at supporting and upgrading traditional markets receive little attention, and in some cases, informal traditional retail channels are even restricted. For instance, the *Joint Circular 08/2008/TTLT-BYT-BGDĐT* regarding food safety in educational institutions introduced measures to prohibit street food vending around schools, particularly when vendors do not meet hygiene standards. In Hanoi, this approach was further tightened by the *2022-2030 Plan 266/KH-UBND*, which extended the prohibition to all street vendors around schools, irrespective of their compliance with hygiene conditions.

Food procurement and food provision. Current policies on food procurement reflect a collaborative effort among various ministries, including the Ministry of health (MOH) and the Ministry of Education and Training (MOET), with a particular focus on school-settings. Several policy documents, such as *Decision 712/QD-TTg* (National Action Program on Child Protection), *Decision 2195/QD-BGDĐT* (Guidelines for Organizing School Meals), and *Directive 584/BGDĐT-GDTC* (Strengthening Nutrition Efforts), provide guidance on school meals and include considerations to ban the sale of unhealthy foods within school canteens. However, these measures are largely non-mandatory, which might lead to inconsistent implementation across educational institutions and limit their effectiveness.

In contrast to substantial political commitment to promoting healthy foods at school, there remains a significant policy gap regarding the regulation of food service activities in other public and private sectors. For instance, while *Decision 2879/QD-BYT* (Guidelines for Diets in Hospitals) sets criteria for meals provided in hospitals, similar comprehensive policies are absent in other administrations and public sector settings. Additionally, in the private sector, policies like *Resolution 07c/NQ-BCH* (Quality of Meals for Employees) encourage healthy food provision in workplaces but do not mandate it, making their impact limited and unlikely to drive significant change.

Food marketing

Food promotion. Vietnamese policies acknowledge the need to address the advertising of unhealthy foods, particularly around schools, as evidenced by various policy documents. However, there are still significant gaps in the regulation of the marketing and sale of highly processed foods and beverages. The current *Advertising Law 16/2012/QH13* mandates that food products must have hygiene, safety, and quality registration certificate, but does not specify restrictions on the types of food and beverage products that can be advertised. *Decree 38/2021/NĐ-CP* (Regulations on Advertising) imposes fines for publishing advertisements that may negatively affect children's well-being but does neither explicitly restrict the promotion of unhealthy foods, nor refer to food in general. Additionally, while *Decision 1294/QD-BYT* (National Strategy on Nutrition until 2025) emphasizes the need to strengthen regulations on food advertising to children, specific and enforceable measures remain underdeveloped. Advertising restrictions in Viet Nam primarily target breast milk substitutes (e.g., *Decree 181/2013/ND-CP*), leaving the broader influence of advertisements for other unhealthy food and beverage products largely unaddressed.

Food labeling. Vietnamese food labeling regulations require packaged food products to include ingredient lists and nutritional information, in line with international standards. This is found in *Decree 43/2017/ND-CP* (Goods Labelling), which specifies the mandatory contents that must be displayed on

food labels. However, regulations remain incomplete due to the lack of clear guidelines and mechanisms for the review and approval of food labels[3]. For example, *Decision 1092/QD-TTg* (Approval of Viet Nam Health Plan) emphasizes the need for labeling processed foods with information on energy, salt, sugar, saturated fat, and trans-fat, yet there is no clear mechanism for reviewing and approving food labels, which opens the door for potentially misleading labeling by manufacturers. In addition, food-related health claims are poorly regulated. *Circular 43/2014/TT-BYT* (Regulating the Management of Functional Foods) mandates that food-related health claims on packaging must be regulated, but it only applies to functional foods (i.e. food with properties that support health and/or prevent diseases, beyond its basic nutritional role), leaving other processed foods without specific regulations regarding health claims.

Furthermore, there are no regulations on front-of-pack supplementary nutrition information systems, which could help consumers assess the healthiness of food products at the point of sale. *Decision 5924/QD-BYT* (Implementation Plan of the Viet Nam Health Program 2021-2025) acknowledges the importance of front-of-pack labeling yet does not provide clear and mandatory guidelines for its implementation. Similarly, there are no regulations requiring menu boards in restaurants to display nutritional information.

Food desirability

Viet Nam's nutritional policies prioritize both dietary guidelines and public awareness to enhance food and nutrition security. To promote these, policies such as *Decision 1294/QD-BYT* (Action Plan for National Strategy on Nutrition until 2025), *Decision 02/QD-TTg* (National Nutrition Strategy for 2021-2030, and vision to 2045), and *Decision 5924/QD-BYT* (Implementation Plan of the Viet Nam Health Program 2021-2025), emphasize the importance of national communication campaigns and nutrition education through mass media campaigns, the use of social networks and online platforms, and targeted communication to specific demographic groups. The focus is on raising public awareness about healthy diets, preventing NCDs, and incorporate nutrition education into existing public health and education initiatives. It also highlights the need to strengthen communication skills among health education and communication staff at both central and local levels. Most of the existing policies in this domain remain non-mandatory.

Food prices and affordability

Food prices. The government has implemented several tax exemptions on food items, including an exoneration of value added tax for food products that have not been (or minimally) processed (*Law 13/2008/QH12*), and no import tax for fresh fruits, vegetables and tubers (*Decree 129/2022/ND-CP*).

---

[3] Subsequent to our data collection (which included updates up to 2022), Viet Nam's Ministry of Health issued Circular 29/2023/TT-BYT. This Circular provides guidelines for labeling nutritional components and values on prepackaged foods, stipulating that by December 31, 2025, producers, traders, and importers must include nutritional composition on food labels. This represents a significant step forward in Vietnam's food labeling regulations.

Various policies have also introduced investment incentives and tax deductions to support the production of safe fruits and vegetables. However, there is no direct financial policies explicitly designed to incentivize healthy dietary choices.

Regarding the taxation of unhealthy foods, several policy documents have acknowledged the necessity of such measures. For instance, *Decision 376/QD-TTg* (National Strategy for Preventing […] Diseases) and *Decision 1092/QD-TTg* (Viet Nam Health Plan) underscore the importance of designing and implementing suitable tax rates to limit the consumption of sugary drinks, processed foods, food additives, and other food products posing health risks. More recently, *Decision 1294/QD-BYT* (National Strategy on Nutrition until 2025) and Decision *02/QD-TTg* (National Nutrition Strategy for 2021-2030, and vision to 2045), issued by MOH, suggest introducing a special consumption tax on sugary beverages. However, these recommendations do not extend to processed foods, indicating a potential shift away from earlier considerations of broader food categories posing health risks. Despite these recent developments, to date there is no excise tax neither on sugary drinks nor on processed foods.

Food affordability. Viet Nam's subsidy policies have traditionally prioritized the stabilization of prices for essential staples like rice, salt, and sugar, as codified in *Law 11/2012/QH13* (Law on Prices). While these measures are crucial for ensuring food security, particularly for vulnerable groups and regions impacted by natural disasters, they do not necessarily contribute to promoting healthier food options. Policies such as *Resolution 34/NQ-CP* (Ensuring National food security toward 2030), or *Decision 01/2012/QD-TTg* (Application of Good Agricultural Practices), provide targeted subsidies aimed at sustaining rice production and encouraging good agricultural practices, yet these initiatives focus on food availability and food safety, rather than on improving affordability of healthier food choices.

Food availability and accessibility

Food availability. We found no evidence of specific policies focused on encouraging food stores and food service outlets to actively promote the availability of healthy foods and limit unhealthy options.

Food accessibility. We found no evidence of zoning laws and policies aimed at regulating the density of unhealthy food outlets or encouraging the development of outlets selling more healthy foods such as fresh fruits and vegetables.
